# Expanding the Chemical Space of Transforming Growth Factor-β (TGFβ) Receptor Type II Degraders with 3,4-Disubstituted Indole Derivatives

**DOI:** 10.1021/acsptsci.3c00371

**Published:** 2024-03-21

**Authors:** Daniel Längle, Stephanie Wojtowicz-Piotrowski, Till Priegann, Niklas Keller, Fabian Wesseler, Elena S. Reckzeh, Karsten Steffens, Christoph Grathwol, Jana Lemke, Maren Flasshoff, Christian Näther, Anna C. Jonson, Andreas Link, Oliver Koch, Gianni M. Di Guglielmo, Dennis Schade

**Affiliations:** † Department of Pharmaceutical & Medicinal Chemistry, Christian-Albrechts-University of Kiel, Gutenbergstrasse 76, 24118 Kiel, Germany; ‡ Department of Physiology and Pharmacology, Schulich School of Medicine and Dentistry, Western University, London N6A 5C1, ON, Canada; § Faculty of Chemistry and Chemical Biology, Technical University Dortmund, Otto-Hahn-Strasse 6, 44227 Dortmund, Germany; ∥ Institute of Pharmacy, University of Greifswald, Friedrich-Ludwig-Jahn-Strasse 17, 17489 Greifswald, Germany; ⊥ Institute of Inorganic Chemistry, Christian-Albrechts-University of Kiel, Max-Eyth-Straße 2, 24118 Kiel, Germany; # Early Chemical Development, Pharmaceutical Sciences, IMED Biotech Unit, AstraZeneca Gothenburg, Mölndal SE-43183, Sweden; ∇ Institute of Pharmaceutical and Medicinal Chemistry and German Center of Infection Research, Münster 48149, Germany; ○ Partner Site Kiel, DZHK, German Center for Cardiovascular Research, 24105 Kiel, Germany

**Keywords:** transforming growth factor-β inhibitor, protein degradation, virtual screen, scaffold hopping, chemical probes, anticancer

## Abstract

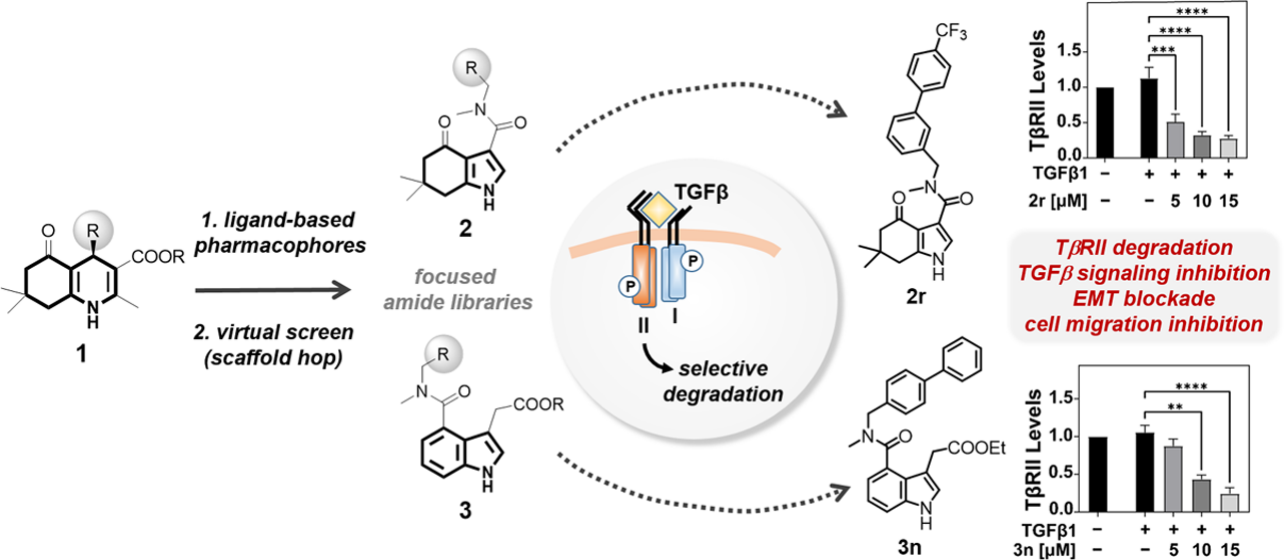

The TGFβ type II receptor (TβRII) is a central player in *all* TGFβ signaling downstream events, has been linked to cancer progression, and thus, has emerged as an auspicious anti-TGFβ strategy. Especially its targeted degradation presents an excellent goal for effective TGFβ pathway inhibition. Here, cellular structure–activity relationship (SAR) data from the TβRII degrader chemotype **1** was successfully transformed into predictive ligand-based pharmacophore models that allowed scaffold hopping. Two distinct 3,4-disubstituted indoles were identified from virtual screening: tetrahydro-4-oxo-indole **2** and indole-3-acetate **3**. Design, synthesis, and screening of focused amide libraries confirmed **2r** and **3n** as potent TGFβ inhibitors. They were validated to fully recapitulate the ability of **1** to selectively degrade TβRII, without affecting TβRI. Consequently, **2r** and **3n** efficiently blocked endothelial-to-mesenchymal transition and cell migration in different cancer cell lines while not perturbing the microtubule network. Hence, **2** and **3** present novel TβRII degrader chemotypes that will (1) aid target deconvolution efforts and (2) accelerate proof-of-concept studies for small-molecule-driven TβRII degradation *in vivo*.

The family of transforming growth factor-β (TGFβ) ligands modulate a plethora of cellular processes in a tissue context-specific manner.^1^ Aberrant regulation of these pathways is associated with a number of diseases, most notably cancer and fibrosis.^2−4^ However, several challenges hamper translation of current anti-TGFβ strategies into broad clinical practice including an acceptable benefit–risk ratio, which certainly depends on the medical indication. Although TGFβ exhibits diverse, oftentimes opposing, roles in tumor pathology, pharmacological targeting of this pathway holds particular promise in anticancer therapy. For the treatment of solid tumors, current concepts in clinical trials aim at reducing excessive levels of TGFβ ligands (e.g., neutralizing antibodies and inhibitors of ligand sequestration as ligand traps) or blocking type I TGFβ receptor (TβRI) kinase activities.^5−7^ The latter encompass classic small molecules, such as the clinical candidates vactosertib (EW-7197),^8^ galunisertib,^9−11^ LY3200882,^12^ GW-788388,^13^ and SD-208^14^ to name a few. A prime goal for designing next-generation TβR inhibitors is limiting off-target toxicities due to a lack of receptor/kinase selectivity. In addition, on-target liabilities—such as cardiac side effects^15^—still present a safety concern raising the question as to how pathological TGFβ should be inhibited. Therefore, it is critical to further explore novel druggable targets and mechanisms for this pathway. With this background knowledge in mind, the specific shutdown of the TGFβ receptor type II (TβRII) has emerged as an attractive option.

Even though the TβRII-TβRI complex initiates signal transduction through canonical (e.g., SMAD-dependent) and noncanonical (e.g., MAPK-, PI3K/Akt-, or Par6/αPKC-dependent)^16−19^ pathways, each receptor has specific functions. Signal activation relies on TGFβ ligand engagement of TβRII, which phosphorylates and activates TβRI. Following receptor complex formation, the kinase activity of both receptors is necessary for epithelial-to-mesenchymal transition (EMT), but the specific TβRII-dependent Par6/αPKC pathway also stimulates RhoA degradation, a precursor event for cell migration.^18−20^ It is not clear whether TβRII confers downstream events exclusively via its kinase activity but bears additional pathology-relevant moonlighting functions. TβRII evidently behaves different from TβRI in terms of localization, trafficking, biosynthesis, and degradation.^21,22^ It is believed that much of TGFβ signaling outputs are regulated through constant shuttling and cycling of TβRII, whereas TβRI is largely recruited upon exogenous ligand-mediated TβRII binding.

Importantly, mislocalization of receptors has been associated with several human diseases.^21^ Increased levels of TβRII in the cytoplasm have been associated with poor prognosis in breast cancer patients.^23^ Mutant TβRII (E221 V/N238I) was identified in human oral squamous cell carcinoma and showed impaired receptor endocytosis with increased TGFβ signal activity.^24^ Moreover, a recent study in breast cancer patients revealed that the levels of TβRII-containing circulating extracellular vesicles appear to correlate with tumor burden, metastasis, and patient survival.^25^

Hence, interfering with TβRII presents a highly attractive antitumor approach but only few strategies have been developed thus far. The TβRII-specific neutralizing antibody LY3022859 (MT-1) demonstrated efficacy against primary tumor growth and metastasis.^26,27^ Only one potent small-molecule TβRII kinase inhibitor has been disclosed very recently.^28^ However, in view of the specific subcellular fates and functions of TβRII—compared to TβRI—with possible activity beyond phosphorylation events, its targeted degradation is an attractive option. Contemporary proximity-induced small-molecule degraders (e.g., PROTACs) have not been described yet. However, physiological lysosomal and proteasomal degradation mechanisms play roles in TβR signaling and homeostasis. Both of these degradation pathways could already be harnessed by TβR degrader modalities:

Pentachloropseudilin (**PClP**) and pentabromopseudilin (**PBrP**) present reversible allosteric inhibitors of nonconventional myosins (Figure 1),^29^ a mechanism that has been postulated to disrupt TβRII trafficking, leading to receptor accumulation in late endosomes followed by its lysosomal degradation.^30,31^ Yet, targeting motor protein-dependent cellular processes that globally impair cytoskeleton integrity and endosomal trafficking raises safety concerns.

**Figure 1:**
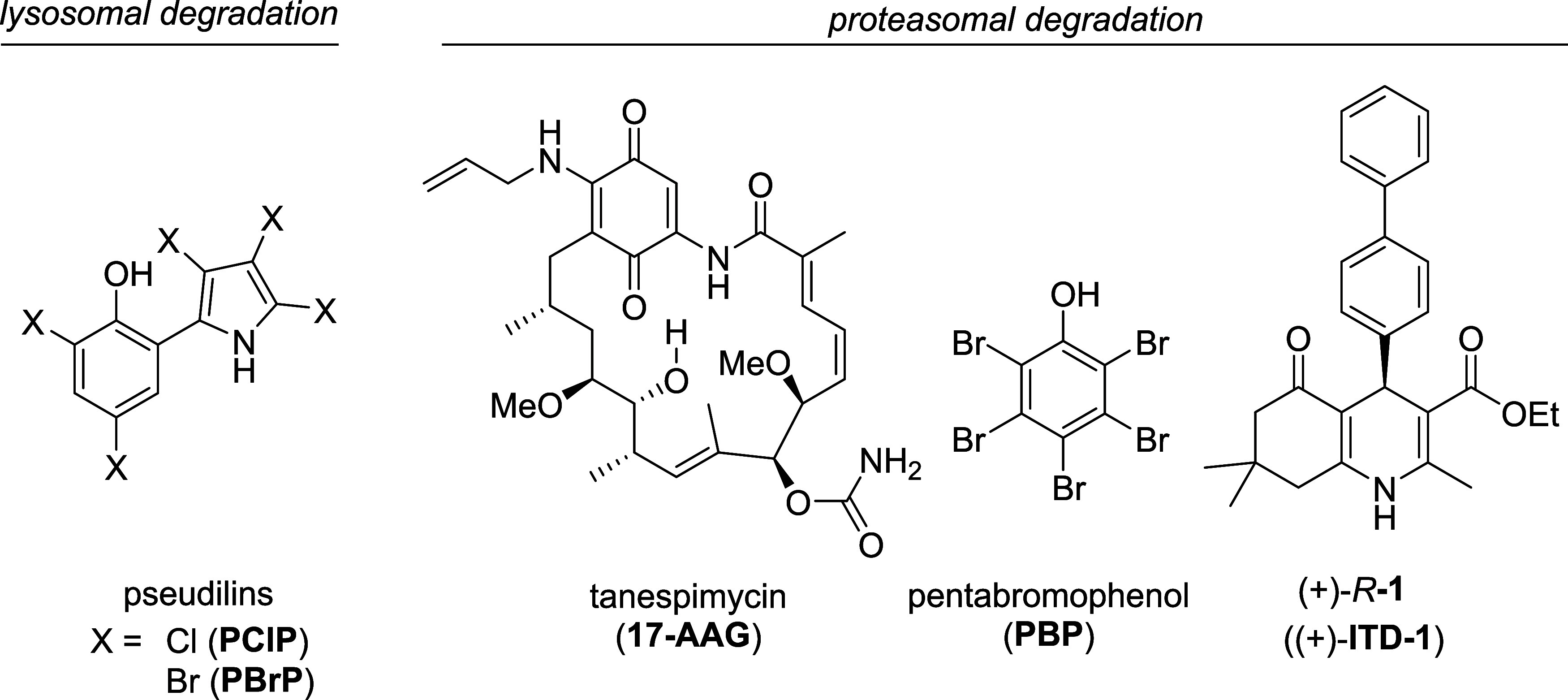
Chemical structures of small-molecule TβR degraders. Compounds are depicted that target lysosomal degradation of TβRII (**PClP**, **PBrP**),^29^ proteasomal degradation of TβRI and TβRII (**17-AAG**),^32^ and TβRII selectively (**PBP**, (+)-**ITD-1**).^33,34^

Early studies described equal proteasomal degradation of both TβRI and TβRII via Smurf-2 E3 ubiquitin ligase.^35^ In this regard, HSP90 functions as a chaperone for type I and II TβRs and was postulated to prevent their membrane raft-mediated proteasomal fate.^32^ HSP90 inhibitors such as tanespimycin (**17-AAG**, Figure 1) were indeed shown to induce the proteasomal degradation of both receptors to the same extent. Pentabromophenol (**PBP**, Figure 1) was reported to induce TβRII clearance via the proteasome.^33^ However, its structurally simple and highly lipophilic nature raises the question whether an attractive druggable target conveys this activity.

We previously discovered a class of *b-*annulated 1,4-dihydropyridines (i.e., (+)-**ITD-1**, Figure 1) and characterized them as unique TGFβ inhibitors as they drive the clearance of TβRII from the plasma membrane as opposed to perturbation of receptor biosynthesis.^34^ Mechanistically, TβRII proteasomal degradation is triggered after 6–8 h while the type I receptor is not affected. Comprehensive structure–activity relationship (SAR) studies provided potent derivatives with cellular IC_50_ values in the submicromolar range and reinforced a specific mode of action that is largely independent of intrinsic compound properties such as lipophilicity.^36−38^ A high-quality set of chemical probes, including photoaffinity-labeling probes,^39^ could be devised with active (+)-enantiomers (*R*-configured)^37^ and inactive (−)-enantiomers that serve as optimal controls for mechanistic studies.

Among the TβRII degraders described to date, the **1**-based chemotype potentially implicates the greatest chance to unravel and harness a high-quality, druggable target for rational drug development. Our current chemoproteomic approaches employ photoaffinity-labeling derivatives from **1** and underline that the compounds do not directly engage with the TβRII but confer an indirect mechanism that culminates in its proteasomal degradation (unpublished data). While target deconvolution efforts are ongoing, we built on the profound cellular SAR data to construct a ligand-based pharmacophore model for virtual screening. The aim was to spark ideas for scaffold hopping toward promising alternative TβRII degrader chemotypes that would share the same mode of action as **1**. The herein presented results highlight the feasibility of this approach. We identified and functionally validated two distinct 3,4-disubstituted indole scaffolds (i.e., tetrahydro-4-oxo-indole **2** and indole-3-acetate **3**) as novel degraders of the TGFβ type II receptor that efficiently blocked EMT and migration of cancer cells.

## Results and Discussion

### Pharmacophore Model-Informed Virtual Screen Proposes Putative TβRII Degrader Scaffolds

Phenotypic drug discovery (PDD) offers high chances to identify and develop first-in-class drug candidates.^40,41^ However, an intrinsic pitfall presents the tedious and resource-intensive deconvolution of the underlying mode of action and involved target(s) after hit validation.^42^ Such efforts hamper the generation of a series of chemotypes that induce desired phenotypes while sharing the exact same mode of action on the molecular level. However, the timely availability of distinct chemotypes for novel (patho)physiology is oftentimes critical for successful translation to advanced proof-of-concept models in vivo.

We have previously employed an unbiased phenotypic screen in stem cells that ultimately furnished a unique class of TGFβ inhibitors.^34,36^ These *b-*annulated 1,4-dihydropyridines (e.g., **ITD-1**, **1a**, Figure 1) inhibit TGFβ signaling via the selective down-regulation of the type II TGFβ receptors by proteasomal degradation. Several SAR studies with >200 derivatives provided potent derivatives with cellular IC_50_ in the submicromolar range.^36,38,43^ Key SAR features included that the TGFβ inhibiting (+)-enantiomers are *R*-configured, *b*-annulation is essential, the *N*1-hydrogen cannot be substituted, the size of the 3-ester groups is restricted and the 4-position should be an electron-deficient biaryl structure.

**Figure 2:**
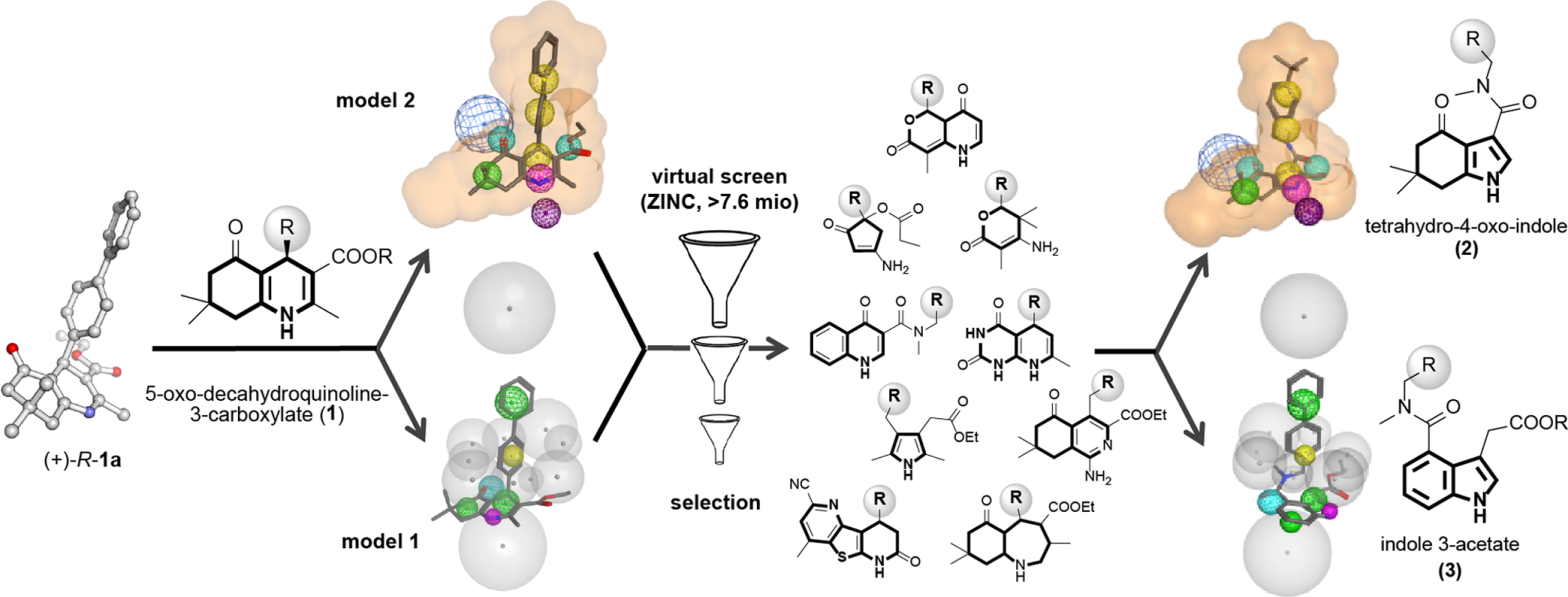
Construction of pharmacophore models and virtual screening suggested indole scaffolds **2** and **3** as putative TβRII degrader chemotypes. Models 1 and 2 were built using MOE and >7.6 million compounds screened with 500 conformers per structure. Model descriptors: Excluded space/volume (gray), ligand volume (orange), hydrophobic space (green), hydrophobic/aromatic (yellow), H-bond acceptor (cyan), H-bond acceptor of the binding partner (blue), H-bond donor (magenta), H-bond acceptor of the binding partner (dark purple).

Building on this knowledge, we herein constructed two ligand-based pharmacophore models for virtual screening (Figure 2). The molecule geometry was obtained from the single-crystal X-ray structure of (+)-*R*-**1a**, which exhibits a characteristic flattened boat conformation with a dihedral torsion angle of 118.9° for the 4-biaryl substituent relative to the 1,4-dihydropyridine core. Model 1 considered this geometry by placing restrictive spheres (gray) above and below (+)-*R*-**1a** as well as surrounding the 4-biaryl substituent. Additional SAR-derived features were defined, including hydrophobic properties for the *b*-annulated ring (green sphere), an H-acceptor for the 5-ketone (cyan sphere), and an H-donor function at *N*1 (magenta sphere). Model 2 was similarly designed but considered the directionality of the H-bonds at the 5- (blue sphere) and *N*1-position (dark purple sphere). In addition, model 2 defined the ligand space more strictly (orange volume).

Models 1 and 2 were used to search the ZINC-lead-like database for potential ligand conformers that fit into one of the models with root-mean-square deviation (RMSD) < 0.83 Å. We excluded all hits containing a dihydropyridine and those lacking a cyclic scaffold. Representative examples of hits are depicted in Figure 2. Additional selection criteria were the number of stereocenters (≤1), synthetic feasibility, and the presence of positions for easy diversification (e.g., by amide coupling) in order to generate focused compound libraries with lead-like qualities.

The most promising hits from virtual screening and filtering possessed an indole core structure with a specific 3,4-disubstitution pattern: Tetrahydro-4-oxo-indole (**2**) closely resembles the *b*-annulated 1,4-dihydropyridine **1** (i.e., decahydro-5-oxo-quinoline). A benzylic amide substituent in 3-position mimics the perpendicular oriented 4-biaryl substituent (relative to the heterocyclic core) of **1a**, while meeting key H-bond interaction requirements. This was also the case for indole-3-acetate (**3**) that carried a benzylic amide in 4-position. In addition, its ethyl ester in 3-position seemed to nicely copy the crucial 3-ester moiety of **1a**.

Together, 3,4-disubstituted indole derivatives **2** and **3** were nominated for the design and synthesis of small amide libraries and comprehensive biological evaluation as TβRII degraders.

### Chemistry

The flattened boat geometry of **1a** with its perpendicular 4-biaryl substituent is not straightforward to fully recapitulate with a distinct core scaffold. Therefore, we reasoned to generate a focused library of distinct amides in 3-position for **2** and 4-position for **3** that covered a broad range of steric and electronic substituents.

**Scheme 1:**
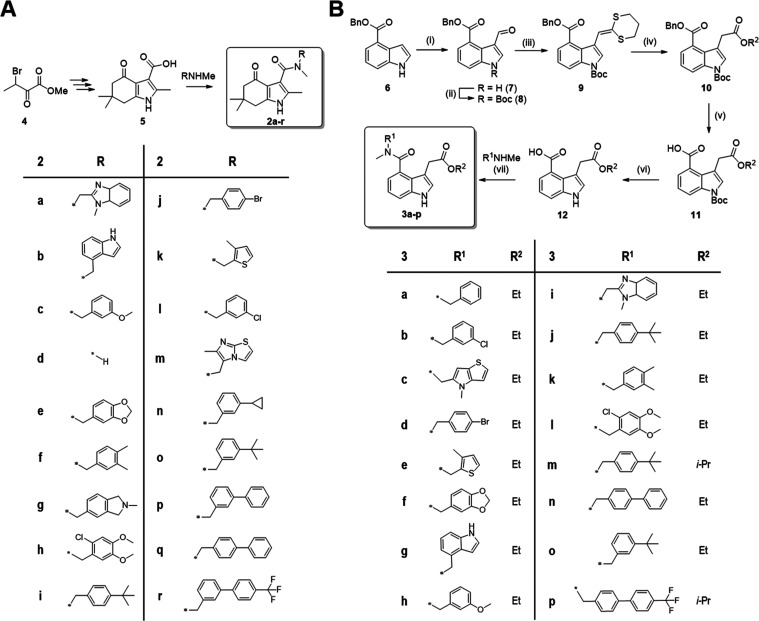
Synthetic Routes to Tetrahydro-4-oxo-indole (**2**) and Indole-3-acetate (**3**) Amide Libraries (A) Carboxylic acid **5** is accessible in 4-steps from brominated **4** (yield: 30%), followed by amide coupling using a hexafluorophosphate azabenzotriazole tetramethyluronium (HATU)/*N*,*N*-diisopropylethylamine (DIPEA) protocol.^44^ Yields for amides **2a**–**r** vary between 15 and 96%. (B) Reaction conditions: (i) POCl_3_, *N*,*N*-dimethylformamide (DMF), 0 °C to room temperature, 3 h, 83%; (ii) Boc_2_O, 4-dimethylaminopyridine (DMAP), CH_3_CN, RT, 16 h, 87%; (iii) (1) 1,3-Dithiane-2-diethylphosphonate,^45^*n*-BuLi, tetrahydrofuran (THF), −78 °C, 1 h, (2) **9** in THF, −78 °C to RT, 90 min, 82%; (iv) For the ethyl ester: AgNO_3_, EtOH, 60 °C, 4 h, 97%; For the *i*-propyl ester: HgCl_2_, *i*-PrOH, 60 °C, sonication, 12 h, 29%; (v) NH_4_HCOO, Pd (10%/C), EtOH, 100 °C, 8 h, 79%; (vi) trifluoroacetic acid (TFA), dichloromethane (DCM), RT, 3 h, 75%; (vii) HATU, DIPEA, DMF, RT, 16 h, 30–98%; Note: Amides **3m,p** were prepared as *i*-Pr esters, when using *i*-PrOH during ketene dithioacetal cleavage (see the Supporting Information (SI)).

We previously disclosed the synthesis of an amide library for tetrahydro-4-oxo-indole (**2**) as multipurpose screening compounds.^44^ Briefly, brominated oxo ester **4** was prepared from commercially available methyl 2-oxobutanoate and was converted in 4-steps toward 3-carboxylic acid (**5**) as the key building block for amide coupling with a series of 18 different *N*-methyl benzylamines to furnish **2a**–**r** (Scheme 1A).

Preparation of indole scaffold **3** turned out a bit more challenging. Notably, 3,4-substitution patterns are underrepresented within the vast chemical space of >6.2 million literature-reported indoles (i.e., <1% are exactly 3,4-disubstituted, SciFinder search). One of the reasons might be that the classic Fisher indole synthesis favors a 3,6- instead of 3,4-disubstituted indole product from the required *meta*-substituted arylhydrazines. Moreover, we found that indoles with a 4-carboxylic acid and methylene-bridged substituent at the 3-position are scarcely reported. To date, only one symmetrically 3,4-disubstituted indole has been described, harboring a 4-carboxylic acid and 3-acetic acid substituent, as well as its corresponding *bis*-methyl ester.^46^ However, their preparation requires a multistep route from 4-cyano-indole under harsh conditions that do not permit orthogonal functionalization of the 3,4-substituents.

By contrast, we aimed for a synthetic route that would allow orthogonal decoration of the two carboxylic acids in 3- and 4-position and deemed commercially available, inexpensive benzyl indole-4-carboxylate (**6**) a suitable starting material (Scheme 1B). Diverse attempts for direct alkylation of **6** at the 3-position proved unsuccessful. Hence, we introduced a 3-formyl group under Vilsmeier conditions in 83% yields, subsequently Boc-protected *N*1 (87%) and built up the 3-acetic acid moiety via homologization. This was achieved by HWE reaction of aldehyde **8** with 1,3-dithiane-2-diethylphosphonate to form ketene dithioacetal **9**, followed by direct conversion to the desired ethyl ester **11** using silver(I)nitrate in good yields (80%, 2 steps). After removal of the Bn- and Boc-protecting groups, we obtained the key building block **12** for the synthesis of the desired amide library. Amide coupling with 16 different *N*-methyl benzylamines was successful using a HATU/DIPEA protocol and furnished **3a**–**p** (yields 30–98%). It should be noted that the newly established route also allowed access to other carboxylic esters. We have tested this option by employing *i*-PrOH as the solvent during ketene dithioacetal cleavage, albeit HgCl_2_ had to be used instead of AgNO_3_ in this step (see the SI). The remaining reaction sequence toward amides **3m,p** worked similarly well compared to the ethyl ester series (**3a**–**l,n,p**).

### Screening and Functional Validation of Scaffolds **2** and **3** as Novel TβR-II Degraders

**Figure 3:**
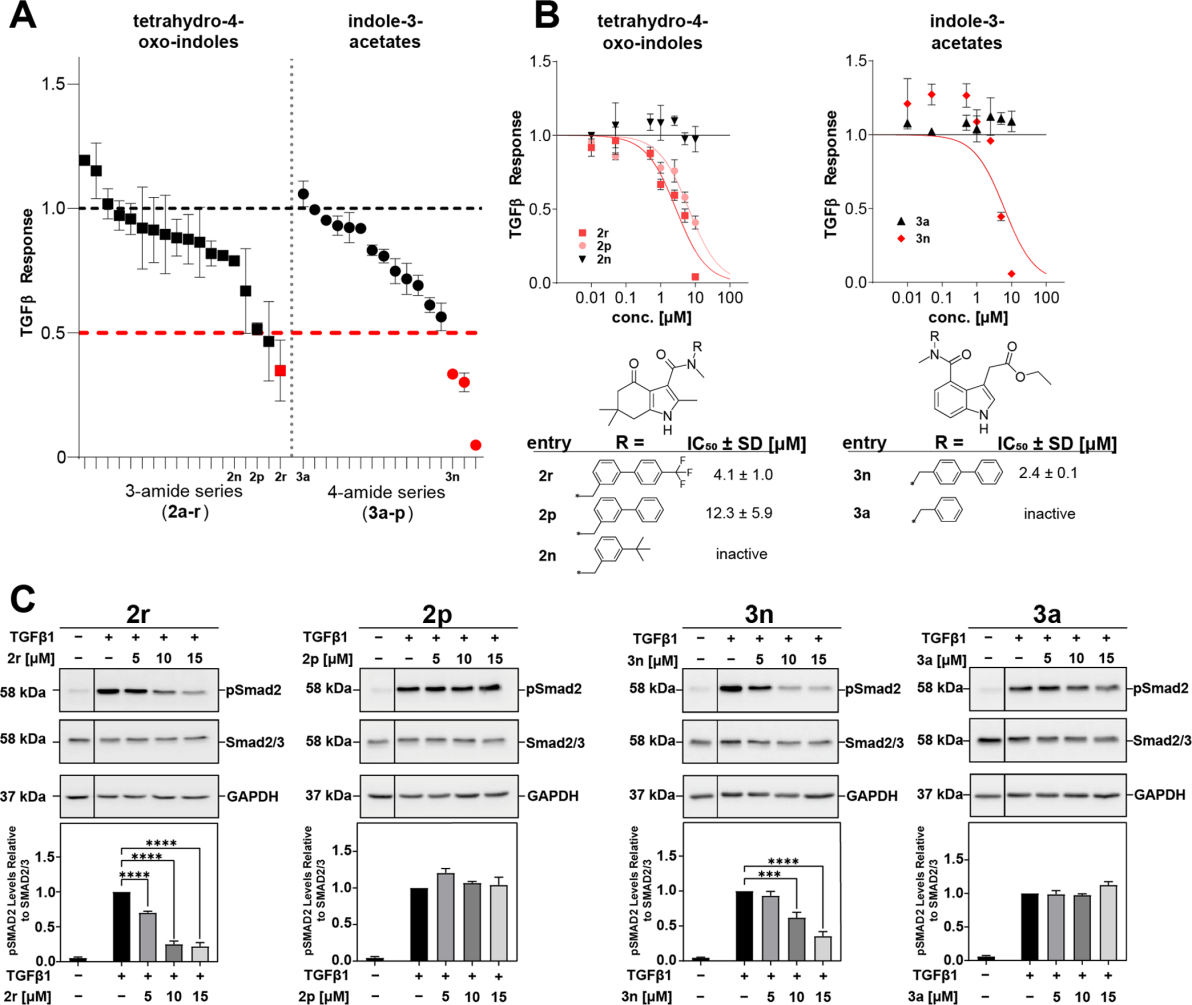
TGFβ inhibition screen of focused amide libraries of indole scaffolds **2** and **3**. TGFβ inhibition activity of compounds **2a**–**r** and **3a**–**p** at 5 μM (A) and dose–response profiles (IC_50_) of selected compounds. (B) Data from an SBE4-*luc* assay in HEK293T cells, *n* = 2–3 independent experiments (mean ± standard deviation (SD), normalized to dimethyl sulfoxide (DMSO) = 1). (C) A549 cells were serum-starved and treated with increasing concentrations of **2r, 2p, 3a**, and **3n** for 24 h, followed by 1 h of TGFβ1 treatment (100 pM). Protein lysates were subjected to Western blotting for pSmad2, Smad2/3, and glyceraldehyde 3-phosphate dehydrogenase (GAPDH). Relative levels of the protein of interest were quantitated using QuantityOne software and graphed as the ratio of pSmad2/Smad2/3; *n* = 3 independent experiments (mean ± standard error of the mean (SEM), normalized to TGFβ1/DMSO = 1. *** *p* ≤ 0.005, **** *p* ≤ 0.0001).

A total of 18 tetrahydro-4-oxo-indoles (**2a**–**r**) and 16 indole-3-acetates (**3a**–**p**) were subjected to screening in a TGFβ/Smad reporter gene assay in HEK293T cells and hits selected for follow-up when the TGFβ response was suppressed <50% at 5 μM (Figures 3A and S1). One tetrahydro-4-oxo-indole (**2r**) exhibited noticeable pathway inhibition, which was confirmed to be dose-dependent with an IC_50_ of 4.1 μM (±1.0 μM) (Figure 3A,B). Three indole-3-acetates inhibited TGFβ responses <50% (i.e., **3n**–**p**), but only **3n** could be confirmed with an IC_50_ of 2.4 μM (±0.1 μM). **3o,p** were excluded due to cell toxicity that appeared to obscure reporter gene assay activity. Results from the reporter gene assay were further validated by assessing downstream phosphorylation of Smad2 (= pSmad2) in the absence (see Figure S2) or presence of TGFβ in A549 cells (Figure 3C). As expected, TGFβ treatment significantly increased pSmad2 levels compared to the control. However, **2r** and **3n** significantly inhibited pSmad2 levels in a dose-dependent manner while **2p** and **3a** treatments resulted in no change, regardless of compound concentration. In the absence of TGFβ stimulation, none of the compounds had an effect on basal Smad2,3 protein levels or phosphorylated Smad2,3 (Figure S3).

Although a thorough SAR analysis would certainly ask for a larger set of derivatives, we could make a few interesting observations already from this data: It seems that indole scaffold **3** more closely mimics the linear 4-biaryl substituent of the original dihydropyridine **1** as it also carries a *para*-biphenyl substituent (= **3n**, Figure 3B). In contrast, the biaryl substituent of tetrahydro-4-oxo-indole **2** needs to be branched (*meta*-substituted) for TGFβ inhibition (= **2r**, Figure 3B). The corresponding linear, *para*-substituted derivative **2q** was inactive (Figure S1). Moreover, the CF_3_ substituent in **2r** was critical for activity when compared to **2p** (Figure 3B,C). This is in agreement with our previous work on **1** that underlined the benefit of an electron-withdrawing group in this position (e.g., Cl in **1b** or CF_3_ in **ITD-ts**).^34,43^ Again, this effect appears to be opposite for the indole **3** scaffold.

Taken together, the design of the two pharmacophore models has successfully furnished two distinct indole-based scaffolds **2** and **3** that share similarities but clearly differ in the geometrical requirements to recapitulate the 4-substituent of the original dihydropyridine **1**.

**Figure 4:**
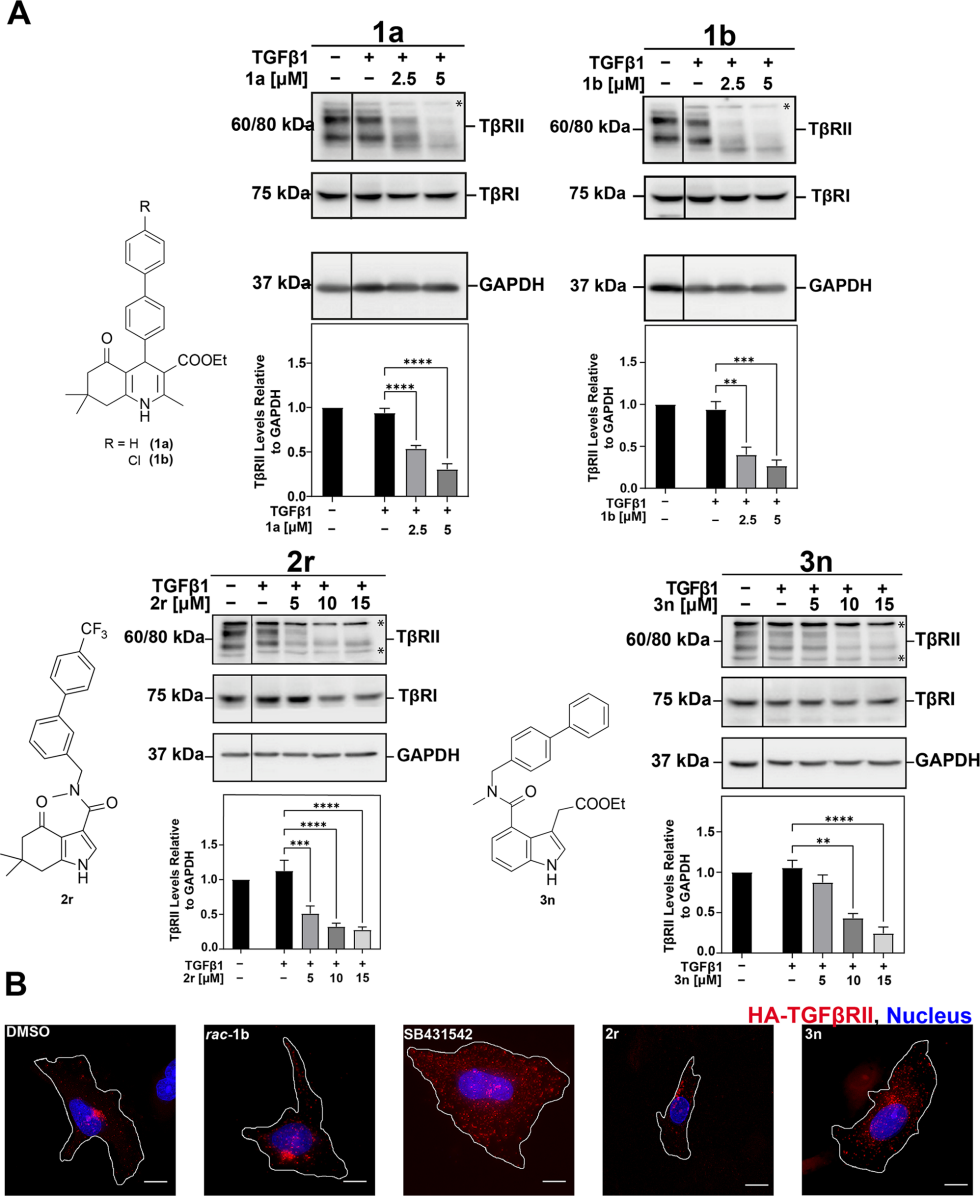
**2r, 3n**, and **1a,b** act through TβRII degradation. (A) A549 cells were serum-starved, then treated with the indicated compounds for 24 h, followed by TGFβ1 (100 pM, 1 h). Protein lysates were subjected to Western blotting for TβRI, TβRII, and GAPDH. Note: Asterisks in the blots represent nonspecific bands above and/or below the glycosylated (upper) and core (lower) TβRII bands. Relative levels of the protein of interest were quantitated using QuantityOne software and graphed as the ratio of TβRII/GAPDH. *n* = 3 independent experiments (mean ± SEM, normalized to DMSO = 1, ** *p* ≤ 0.01, *** *p* ≤ 0.001; **** *p* ≤ 0.0001). (B) Mv1Lu cells expressing extracellularly HA-tagged TβRII were treated with the indicated compounds (10 μM) for 22 h, then incubated with anti-HA antibody (at 4 °C) to tag the extracellular domain of TβRII, followed by 1 h incubation at 37 °C to allow for receptor internalization and co-staining with AF568 (red). Nuclei were stained with 4′,6-diamidino-2-phenylindole (DAPI) (blue), and cells were visualized using an Olympus CKX53 microscope (40×, scale bar = 10 μm).

Next, **2r** and **3n** were taken further for functional validation as actual TβRII degraders and compared side-by-side with dihydropyridines **1a** and **1b** (Figure 4A). Consistent with data from Figure 3C, the Western blot results revealed that **1a,b**, **2r**, and **3n** significantly decreased TβRII steady-state levels in a dose-dependent manner, both in the presence and absence of TGFβ (Figures 4A and S3A). Importantly, TGFβ type I receptor levels were not affected by any of the compounds, which has been previously characterized as a unique feature of **1** as the “first-in-class” small-molecule TβR degrader that selectively induced type II but not type I receptor degradation.^34^ Another characteristic criterion of the new TβRII degraders was the receptor kinase-independent action on the pathway. Classic TβR kinase inhibitors such as **SB431542** entirely block early internalization of the receptors (Figure 4B).^34^ However, TβRII degraders such as **1** do not interfere with this instant TGFβ-dependent endocytosis process and the new scaffolds **2** and **3** share this activity as shown in Figure 4B.

To this end, **2r** and **3n** could be validated to share the same mode of action as TβRII degraders as **1**. They inhibit TGFβ/SMAD signaling via TβRII degradation without significantly affecting TβRI levels or impeding early receptor internalization.

We next sought to test whether this also holds true in a more complex, disease-relevant context. A hallmark of many advanced tumors involves the loss of an epithelial phenotype, characterized by apical and basolateral membrane domains, and the gain of a mesenchymal phenotype, in which cells lose cell–cell adhesion and become more motile and invasive.^47^ Termed EMT, the process involves the loss of cell junctions, and results in the progression to an invasive phenotype.^48^ The addition of TGFβ to epithelial cells, including non-small-cell lung cancer (NSCLC) cells, induces EMT and increases cell migration.^49^ After TGFβ-dependent EMT occurs, polarity complex proteins are confined to the leading edge of migrating cells.^50,51^ We observed that TGFβ receptors colocalize with members of the polarity complex of such cells and regulate both canonical and noncanonical signaling pathways.^17,18,20,52^ Thus, targeting all of the downstream signaling pathways from the TGFβ receptor complex would be necessary to fully inhibit EMT.

Based on the integral role of the TGFβ signaling pathway in tumor progression and metastasis, we wanted to study if the TβRII degrader chemotypes **1**, **2**, and **3** all comparably affected TGFβ-dependent EMT. Given that EMT inhibition by **1** has not been investigated before, and to use high-quality controls for these experiments, we separated the enantiomers of **1b** by supercritical fluid chromatography (SFC) (Figure S6). The absolute configuration of the inactive (−)-enantiomer (= *S*-configured) was determined by single X-ray structure analysis (Figure S6), which is in agreement with the previously determined *R*-configuration of bioactive (+)-**1a.**^37^

**Figure 5:**
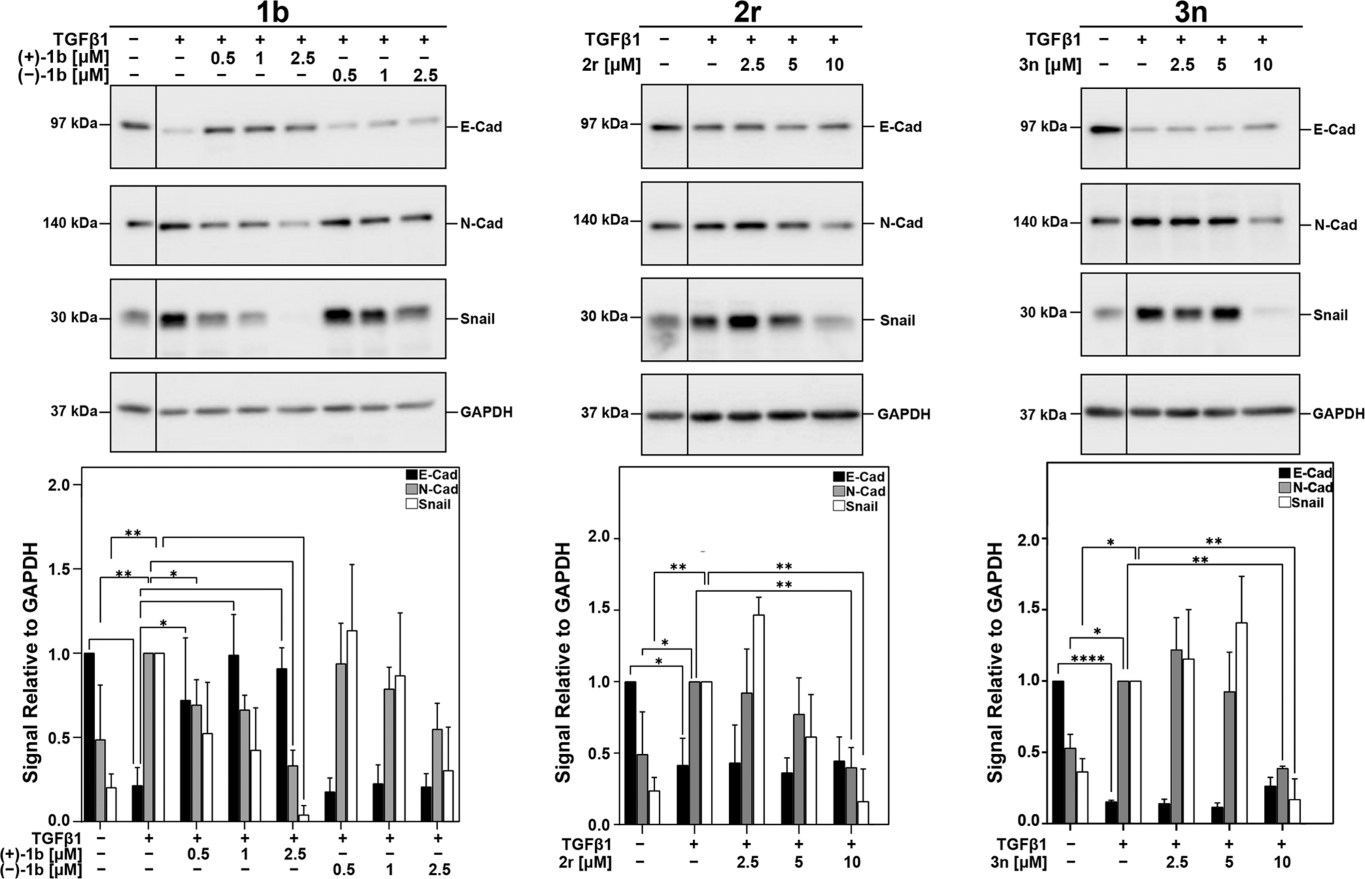
**2r, 3n**, and **1b** block epithelial-to-mesenchymal transition. A549 cells were serum-starved, then treated with increasing concentrations of (+)-**1b**, (−)-**1b, 2r**, or **3n** and 100 pM TGFβ1 (48 h). Protein lysates were subjected to Western blotting for E-cadherin (E-cad), N-cadherin (N-cad), and Snail levels. Relative levels of the protein of interest were quantitated using QuantityOne software and graphed as the ratio of protein of interest/GAPDH. *n* = 3 independent experiments (mean ± SEM, normalized to DMSO for E-Cad and normalized to TGFβ1/DMSO for N-Cad and Snail, * *p* ≤ 0.05, ** *p* ≤ 0.01, *** *p* ≤ 0.001; **** *p* ≤ 0.0001).

The best way to study the EMT program is through the visualization of epithelial (e.g., E-Cadherin) and mesenchymal (e.g., Snail and N-Cadherin) markers. We observed that after 48 h of treatment, TGFβ induced a significant E-cadherin loss and Snail and N-cadherin gain compared to untreated cells (Figure 5). However, in combination with the active (+)-**1b** enantiomer, TGFβ-dependent E-cadherin loss is prevented, returning almost to control levels. Furthermore, (+)-**1b** inhibited the TGFβ-dependent increase in Snail and N-cadherin levels. No effect on these proteins was observed when cells were treated with the inactive enantiomer, (−)-**1b**. Strikingly, both **2r** and **3n** exhibited the same efficacy on Snail and N-Cadherin reduction as (+)-**1b**. In contrast to (+)-**1b**, a rescue of TGFβ-induced E-Cadherin was not observed for **2r** and **3n**, which might be due to their overall lower potencies in this setup (ca. 5- to 10-fold).

Nevertheless, the herein observed strong inhibition of the TGFβ-dependent EMT program by pharmacological TβRII degradation is promising, as EMT occurs in epithelial tumor cells undergoing cytoskeletal rearrangement.^53^ All TβRII degrader chemotypes **1**, **2**, and **3** efficiently blocked the critical formation of a mesenchymal phenotype as a key requirement for metastasis.

### TβRII Degraders Modulate Stress Fiber Formation and Migration of Different Cancer Cell Lines

**Figure 6:**
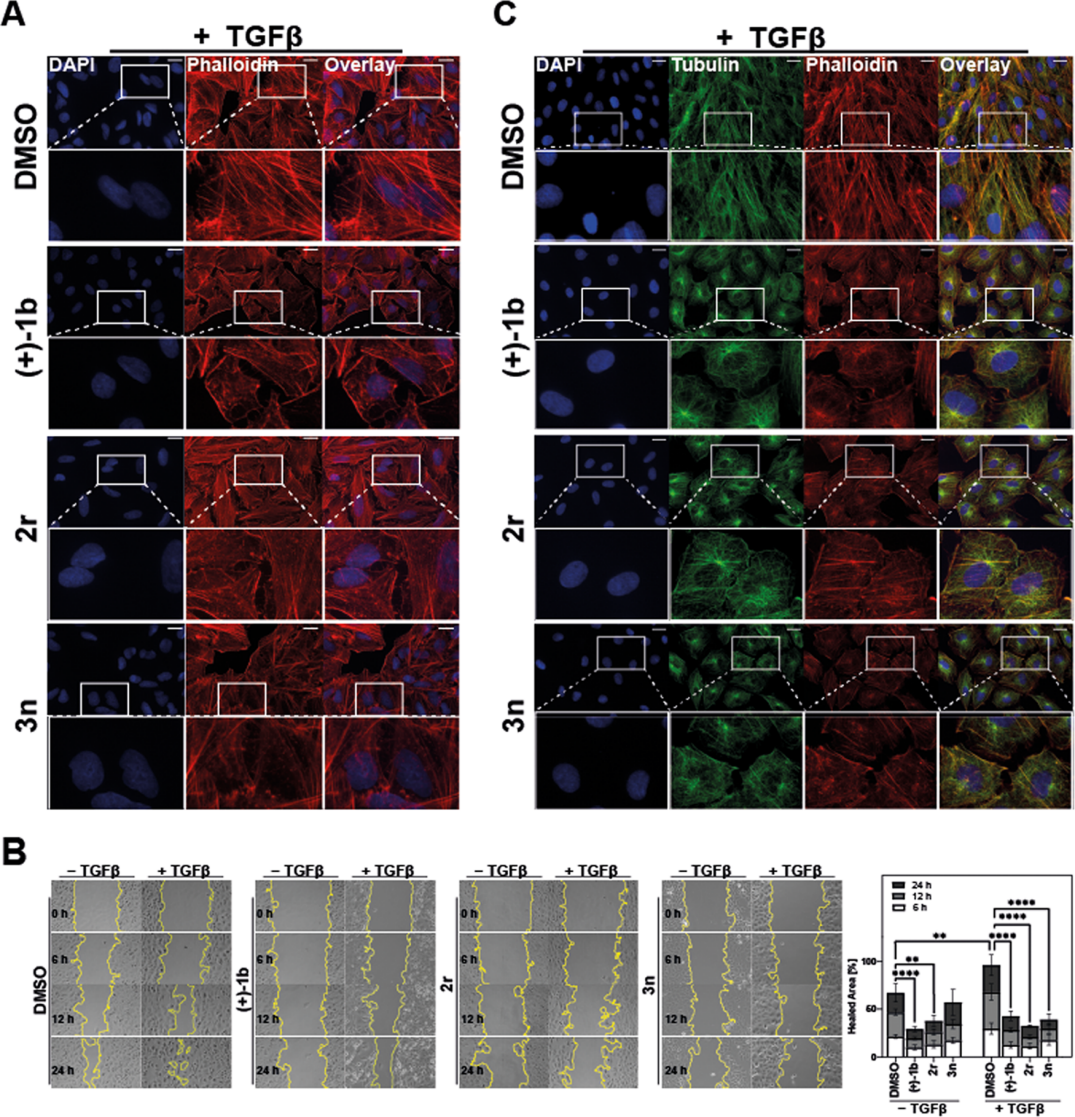
TβRII degrader chemotypes **1, 2**, and **3** modulate stress fiber formation and block cell migration. (A) A549 cells were serum-starved, then treated with the indicated compounds (1 μM **1b**, 5 μM **2r** and **3n**) and 100 pM TGFβ1 for 48 h. Cells were stained with DAPI (nuclei, blue) and AF555-Phalloidin (red) to visualize cortical actin in control cells or stress fibers in TGFβ-treated cells (Olympus IX 81 microscope, 40×, scale bar = 20 μm). (B) H1299 cells were serum-starved and treated with the indicated compounds (1 μM **1b**, 5 μM **2r** and **3n**) and 100 pM TGFβ1 for 24 h. After scraping, cells were immediately imaged (= 0 h), and compound/TGFβ treatment continued for another 24 h with images taken at 6, 12, and 24 h (Leica DMI6000 B microscope, 10×). Scrape area was quantified using the wound healing size tool plugin in ImageJ. Percentage of migration was calculated and plotted as a bar graph. *n* = 3 independent experiments (mean ± SEM, normalized to TGFβ1/DMSO = 100%, ** *p* ≤ 0.01, **** *p* ≤ 0.0001). (C) Rat2 fibroblasts were serum-starved and treated with the indicated compounds (1 μM **1b**, 5 μM **2r** and **3n**) and TGFβ for 48 h. Cells were immunostained against tubulin (green) and co-stained with AF555-Phalloidin (F-actin, red), and DAPI (nuclei, blue) (Olympus IX 81 microscope, 40×, scale bar = 20 μm).

To further analyze the effect of the new TβRII degraders on TGFβ-dependent functional outcomes, A549 NSCLC cells were plated on glass coverslips and treated with **1b**, **2r**, or **3n**, in the presence or absence of TGFβ ligand. We processed the cells for fluorescence microscopy and stained filamentous actin (F-actin) with AF555-labeled phalloidin. In control cells, F-actin is cortical and the cells are in close proximity to each other (Figure S4). However, in TGFβ-treated cells, there is an observable change in morphology: they are more elongated, and they form actin stress fibers (Figure 6A). When cells were treated with (+)-**1b** or **3n** in the presence of TGFβ, we observed a clear decrease in stress fiber formation. However, **2r** differed from the other compounds as it seemed to induce the formation of actin stress fibers even in the absence of TGFβ (Figure S4). This finding does not question its performance as a TGFβ inhibitor but rather points at an off-target activity that is not shared by the other TβRII degrader chemotypes and should be kept in mind for further development of the tetrahydro-4-oxo-indole scaffold **2**.

Stress fibers are contractile, actomyosin bundles that aid in cell migration.^54^ Therefore, the next step was to analyze whether the TβRII degraders inhibit cell migration using a wound scrape assay. When H1299 NSCLC cells were treated with the TβRII degraders, we observed that cell migration, both in the absence and presence of TGFβ, was significantly inhibited by all three chemotypes **1**, **2**, and **3** (Figure 6B). Notably, upon co-treatment with TGFβ, all chemotypes inhibited cell migration to the same extent over time despite their different cellular potencies. This finding underlines the overall efficacy of targeting TβRII for degradation (i.e., efficacy versus potency).

Since we observed inhibition of migration in both the absence and presence of TGFβ and the compounds, we wanted to ensure that this was not due to global perturbation of microtubules. Such activity would not only be a safety concern but compromise a specific mode of action for TβRII degradation. For example, this is an issue for the myosin-inhibiting pseudilins (Figure 1) as lysosomal TβRII degraders.^30,31^ Our previous work showed that the synthetic triterpenoid 2-cyano-3,12-dioxooleana-1,9-dien-28-oic acid (CDDO) prolonged TGFβ signaling by delaying the degradation of ligand-engaged cell surface receptors in Rat2 fibroblasts.^55^ CDDO affected both the microtubule and actin cytoskeletons by targeting microtubule-capping proteins or Arp2/35.^56^ Hence, Rat2 fibroblasts serve as an excellent model system to assess the microtubule network.

However, when we treated Rat2 fibroblasts with (+)-**1b**, **2r**, and **3n** in the absence or presence of TGFβ, and immunostained cells against tubulin, we did not observe any differences in microtubule structure compared to the control (Figure 6C). Interestingly, the microtubules of TGFβ-treated cells were visibly affected, and (+)-**1b**, **2r**, and **3n** treatment in combination with TGFβ largely reverted microtubule morphology to that of TGFβ-untreated cells (Figure S5), further confirming that these compounds do indeed inhibit TGFβ-driven changes.

Taken together, our results show that the TβRII degrader chemotypes **1**, **2**, and **3** inhibit TGFβ signaling leading to inhibition of TGFβ-dependent EMT and actin stress fiber formation, thereby efficiently blocking cell migration, but without affecting the microtubule network. Thus, **2** and **3** seem to conclusively phenocopy the TβRII degrading mechanism of **1** in several cancer cell line models.

## Conclusions

Pharmacological targeting of the TGFβ signaling pathway is well recognized for disease management, most notably for fibrosis and cancer. In advanced and metastatic cancer, TGFβ expression correlates with shortened duration of progression-free and overall survival.^57,58^ Although type I and type II TGFβ receptors act in a complex to stimulate signal transduction, each receptor subtype accesses different signaling effectors.^17,20^ Targeting TβRI would not inhibit the entirety of downstream signaling pathways. However, TβRII is the common factor in all TGFβ signaling, and we found that it is susceptible to pharmacological and genetic perturbations that inhibit TGFβ-dependent EMT of NSCLC cells.^59,60^ Several lines of evidence suggest extraordinary roles of TβRII in cancer progression and aggressiveness.^23−25^

Hence, the development of novel specific, anti-TβRII small molecules holds great promise to inhibit TGFβ signaling events that are linked to metastasis such as in NSCLC. In view of the hardly investigated, complex roles of TβRII in tumor biology, its targeted degradation appears particularly attractive compared to classic kinase inhibition. *b*-Annulated 1,4-dihydropyridine **1** presented a “first-in-class”, selective TβRII degrader with a unique mode of action that does not affect the type I receptor.^34^ Notably, these compounds do not seem to directly engage with TβRII for proximity-induced degradation. We herein aimed at devising alternative TβRII degrader chemotypes that would share the same mode of action as **1**.

Building on comprehensive SAR data for **1**, ligand-based pharmacophore models were designed and used for virtual screening. As a result, two distinct 3,4-disubstituted indole scaffolds were identified as putatively new TβRII degraders, i.e., tetrahydro-4-oxo-indole **2** and indole-3-acetate **3**. Focused amide libraries were generated for each scaffold, screened for TGFβ inhibition, and confirmed **2r** and **3n** as the most potent derivatives from each series. Importantly, chemotypes **2** and **3** could be validated to fully recapitulate the ability of **1** to efficiently and selectively degrade the TGFβ type II receptor, thereby blocking the critical formation of a mesenchymal phenotype during EMT in different lung cancer cell lines. Consequently, **1**–**3** strongly inhibited TGFβ-stimulated migration of cancer cells, while perturbation of the microtubule network was excluded as an undesired, TβR-unspecific mode of action.

To the best of our knowledge, the presented study is a rare example for successfully transforming cellular SAR data into a predictive ligand-based pharmacophore model allowing scaffold hopping for further development. However, in view of the desirable criteria for high-quality chemical probes, the new chemotypes must be optimized toward submicromolar cellular potency in the future to fully harness their utility as alternate chemical probes for target validation, on/off-target, and safety profiling. Although **2r** and **3n** are (yet) less potent than the SAR-optimized eutomer (+)-**1b**, they present high-quality starting points for future optimization. Elucidation of the responsible target and precise molecular mechanism will certainly accelerate these efforts, which is an ongoing quest in our groups. In this regard, the availability of **2r**, **3n** (and closely related inactive analogues thereof) as additional chemical probes (besides **1**) will aid in these endeavors. Furthermore, the availability of a distinct set of chemotypes might accelerate in vivo proof of concept and efficacy studies for small-molecule-mediated TβRII degradation. It will be interesting to see whether selective, nonkinase activity targeting of TβRII might overcome on- and off-target liabilities of established TβRI inhibitors.

## Experimental Section

### Chemistry

#### General

Unless otherwise stated, all reagents were obtained from commercially available sources and were used without additional purification. The reaction progress was monitored via thin-layer chromatography (TLC) with silica gel plates (thickness 250 mm, F-254) under UV light. Flash column chromatography was performed on one of the following systems: CombiFlash Rf200 (Axel Semrau GmbH & Co KG, Sprockhövel, Germany), CombiFlash Nextgen 300 (Axel Semrau GmbH & Co KG, Sprockhövel, Germany), or PuriFlash4250 (Interchim, Montluçon, France) using prepacked silica gel columns (normal phase and reversed phase, particle size 0.015–0.050 mm) provided by Interchim (Montluçon, France) or Teledyne Isco (Axel Semrau GmbH & Co KG, Sprockhövel, Germany). NMR spectra of compounds were recorded on a Bruker Avance III 400 spectrometer (400 MHz, software: Bruker TopSpin 3.6.0). Chemical shifts were reported as ppm (δ) relative to the solvent (CDCl_3_ at 7.26 ppm (^1^H) and 77.0 ppm (^13^C), DMSO-*d*_6_ at 2.50 ppm (^1^H) and 39.5 ppm (^13^C), or tetramethylsilane (TMS) (0.0(0) ppm)) as internal standard. Coupling constants (*J*) are expressed in hertz (Hz). Multiplicities are abbreviated as s = singlet, d = doublet, t = triplet, quart = quartet, dd = doublet of doublet, m = multiplet, br. = broad. Low-resolution mass spectra were recorded on a Bruker amaZon SL ion trap mass spectrometer (Bruker Daltonik, Bremen, Germany) with positive, negative, or alternating polarity. Samples were introduced into the mass spectrometer after separation using Agilent Poroshell 120 EC-C18 column (50 mm × 3.0 mm 2.7 μm particle size) with gradient elution, starting with 97% 0.01% acetic acid and finishing 97% acetonitrile using an Agilent 1260 Infinity high-performance liquid chromatography (HPLC) system (Waldbronn, Germany). High-resolution mass spectra were recorded on an Orbitrap spectrometer (Thermo Fisher Scientific, Waltham) via direct injection of the sample or on a Shimadzu LCMS-IT-TOF by using electron spray ionization. Chemical yields refer to isolated pure substances unless otherwise noted. The purity of the synthesized compounds was determined by HPLC via calculating the percentage of the product peak integral relative to the sum of all observed peak integrals at 254 nm on a Waters Alliance 2695 HPLC system (Waters Cooperation, Milford) using a Phenomenex Gemini C18 (250 mm × 4.6 mm 5 μm particle size) or on a Shimadzu HPLC system using a LiChroCART (250 mm × 4 mm) with LiChrospher 100 RP-18e (5 μm) with gradient elution, starting with 95% aqua bidist. and finishing with 100% acetonitrile. The purity for all synthesized compounds used for biological testing was >96%, unless otherwise noted. Optical rotation values of single enantiomers were determined by using a polarimeter Model No. SN 4180150223 (A. KRÜSS Optronic GmbH, Hamburg Germany). Additional physical/spectroscopic data can be found in the extended experimental section in the Supporting Information.

#### Ethyl 4-[4′-(4″-Chlorophenyl)phenyl]-2,7,7-trimethyl-5-oxo-1,4,5,6,7,8-hexahydroquinoline-3-carboxylate (**1b**)

The title compound was obtained according to Längle et al. as a racemic, colorless solid (76% yield). All spectroscopy data were found to be in accordance with the literature.^43^^1^H NMR (400 MHz, DMSO-*d*_6_): δ = 0.87 (s, 3H), 1.04 (s, 3H), 1.15 (t, 3H, *J* = 7.3 Hz, 1H), 1.97–2.01 (m, 1H), 2.15–2.19 (m, 1H), 2.30 (s, 3H), 2.30–2.35 (m, 1H), 2.40–2.43 (m, 1H), 3.99 (q, 2H, *J* = 7.3 Hz), 4.90 (s, 1H), 7.23–7.26 (m, 2H), 7.44–7.48 (m, 2H), 7.49–7.54 (m, 2H), 7.60–7.65 (m, 2H), 9.13 ppm (s, 1H). ^13^C NMR (101 MHz, DMSO-*d*_6_): δ = 14.4, 19.7, 27.5, 29.5, 30.1, 32.9, 36.5, 41.4, 50.8, 60.0, 106.2, 112.3, 126.6, 128.3, 128.7, 128.9 133.0, 137.7, 139.9, 143.4, 146.6, 147.8, 167.7, 195.5 ppm. LRMS (ESI) *m*/*z* = 450 [M + H]^+^.

#### Enantiomeric Resolution of **1b**

The enantiomeric resolution was accomplished by supercritical fluid chromatography (SFC) on a Supersep 150 system with a YMC Chiral ART Amylose-SA column (YMC Europe GmbH, Dienslaken, Germany, 250 mm × 30 mm, 5 μm). The mobile phase consisted of an isocratic mixture of 16% ethanol (EtOH) and diethylamine (DEA) (100/20 mM) in carbon dioxide (CO_2_), flowing at a rate of 130 mL/min with a pressure of 130 bar at 30 °C. The enantiomers were detected using a PDA detector at 254 nm. To verify the enantiomeric purity, a YMC Chiral ART Amylose-SA column (YMC Europe GmbH, Dienslaken, Germany, 150 mm × 4.6 mm, 3 μm) was used. The mobile phase consisted of 16% EtOH/DEA (100/20 mM) in CO_2_, flowing at a rate of 3.5 mL/min under a pressure of 120 bar at 40 °C. The enantiomers were detected using a PDA detector at a wavelength of 220 nm. Chiral purity was determined to be >99.9%*ee* ((+)-enantiomer) and 99.2%*ee* ((*−*)-enantiomer) (see SI, Figure S6). The optical rotations of the enantiomers were measured in chloroform (*c* = 0.4) and were found to be [*a*]_D_^19^ = +65.5 ((+)-enantiomer) and [*a*]_D_^19^ = −59.5 ((*−*)-enantiomer).

#### 4-Oxo-4,5,6,7-tetrahydro-1*H*-indole-3-carboxamides (**2a**–**r**)

The synthesis of the 3-amide series has been previously described by our groups.^44^ The synthetic protocol for compound **2r** was adapted as follows:

#### *N*,2,6,6-Tetramethyl-4-oxo-*N*-[(4′-trifluoromethyl-1,1′-biphenyl-3yl)methyl]-4,5,6,7-tetrahydro-1*H*-indole-3-carboxamide (**2r**)

2,6,6-Trimethyl-4-oxo-4,5,6,7-tetrahydro-1*H*-indole-3-carboxylic acid^44^ (63 mg, 0.29 mmol), HATU [O-(7-Azabenzotriazol-1-yl)-*N,N,N′,N*′-tetramethyluronium-hexafluorphosphat] (220 mg, 0.58 mmol), and *N*-methyl-1-(4′-(trifluoromethyl)-[1,1′-biphenyl]-3-yl)methanamine^44^ (115 mg, 0.43 mmol) were dissolved in dried DMF (3 mL). To the stirred solution, DIPEA (252 μL, 1.45 mmol) was added. The reaction mixture was covered with inert argon gas and stirred for 24 h at 60 °C. After completion, the reaction mixture was poured into cold water (10 mL) and extracted with ethyl acetate (3 × 50 mL). The combined organic layers were washed with brine (50 mL), dried over Na_2_SO_4_, and the solvent was evaporated. After flash chromatography (EtOAc), the desired product was obtained as a colorless solid (114 mg, 0.24 mmol, 84%). All spectroscopy data were found to be in accordance with the literature.^44^^1^H NMR (400 MHz, DMSO-*d*_6_): δ = 1.05 (s, 6H minor isomer), 1.06 (s, 6H major isomer), 1.99 (s, 3H minor isomer), 2.11 (s, 2H minor isomer), 2.13 (s, 3H major isomer), 2.24 (s, 2H major isomer), 2.63 (s, 2H major isomer), 2.74 (s, 3H major isomer, 2H minor isomer), 2.88 (s, 3H minor isomer), 4.32 (d, 1H, *J* = 15.4 Hz, minor isomer) 4.57 (d, 1H, *J* = 15.4 Hz, major isomer, 1H, *J* = 15.4 Hz, minor isomer), 4.90 (d, 1H, *J* = 15.4 Hz, major isomer), 7.16 (d, 1H, *J* = 7.8 Hz, minor isomer), 7.39–7.47 (m, 1H major isomer, 1H minor isomer), 7.48–7.52 (m, 2H major isomer), 7.59–7.65 (m, 1H major isomer, 2H minor isomer), 7.71–7.85 (m, 2H major isomer, 2H minor isomer), 7.94–8.00 (m, 2H major isomer, 2H minor isomer), 11.34 (br s, 1H minor isomer) 11.37 ppm (br s, 1H major isomer). ^13^C NMR (101 MHz, DMSO-*d*_6_): δ = 9.3, 9.4, 11.4, 11.4, 15.0, 15.0, 28.1, 28.3, 28.7, 32.7, 35.1, 35.3, 35.8, 35.8, 36.2, 49.8, 49.8, 52.3, 52.3, 113.2, 113.2, 116.4, 166.4, 125.8, 126.0, 126.2, 126.2, 126.4, 127.4, 128.5, 128.6, 129.3, 129.6, 139.1, 139.2, 139.2, 139.3, 141.5, 141.5, 167.7, 167.7, 191.7, 191.8 ppm; LRMS (ESI) *m*/*z* = 469.1 [M + H]^+^.

#### Benzyl 1*H*-Indole-4-carboxylate (**6**)

1*H*-Indole-4-carboxylate (1.00 g, 6.21 mmol) and NaHCO_3_ (1.30 g, 15.51 mmol) were ground together and dried in a high vacuum for 30 min. Then, dry dimethylformamide (10 mL) was added, followed by benzyl bromide (1.47 mL, 2.12 g, 12.42 mmol) at room temperature and the suspension was stirred for 48 h. After completion of the reaction, the reaction mixture was poured into a saturated NaHCO_3_ solution (20 mL). After extraction with ethyl acetate (3 × 50 mL), the combined organic layers were washed with water (5 × 50 mL) and brine (50 mL), dried over Na_2_SO_4_, and the solvent was evaporated. After flash chromatography (cyclohexane/ethyl acetate 9:1), the desired product was obtained as a pale-yellow oil, which crystallized as a colorless solid upon storage at 4 °C (1.23 g, 4.90 mmol, 79%). All spectroscopy data were found to be in accordance with the literature.^61^*R_f_* = 0.26 (cyclohexane/ethyl acetate 3:1). ^1^H NMR (400 MHz, DMSO-*d*_6_): δ = 11.50 (s, 1H), 7.79 (dd, 1H, *J* = 7.5, 1.0 Hz), 7.71 (dt, 1H, *J* = 8.1, 0.9 Hz), 7.44–7.54 (m, 3H), 7.31–7.44 (m, 3H) 7.21 (t, 1H, *J* = 7.8 Hz), 6.92–6.94 (m, 1H), 5.41 (s, 2H). ^13^C NMR (101 MHz, DMSO-*d*_6_): δ = 166.7, 136.8, 136.6, 128.6, 128.1, 128.1, 128.0, 127.0, 122.4, 120.3, 120.2, 116.9, 102.1, 65.8. LRMS (ESI) *m*/*z* = 252 [M + H]^+^.

#### Benzyl 3-Formyl-1*H*-indole-4-carboxylate (**7**)

A solution of benzyl 1*H*-indole-4-carboxylate (**6**) (1.40 g, 5.57 mmol) in dry dimethylformamide (10 mL) was cooled to 0 °C and phosphorus oxychloride (1.53 mL, 16.71 mmol) was added dropwise over a period of 10 min. The reaction mixture was warmed to room temperature and stirring was continued for 3 h. Then, the solution was poured on ice (30 g) and the pH was neutralized with a saturated NaHCO_3_ solution. After extraction with ethyl acetate (3 × 50 mL), the combined organic layers were washed with water (2 × 50 mL) and brine (50 mL), dried over Na_2_SO_4_, and the solvent was evaporated. After flash chromatography (cyclohexane/ethyl acetate 3:2), the desired product was obtained as a pale-yellow oil, which crystallized as a colorless solid upon storage at 4 °C (1.29 g, 4.62 mmol, 83%). For large-scale reactions (>5g of benzyl 1*H*-indole-4-carboxylate), the workup was adapted for easier handling: After hydrolysis of the reaction mixture, the suspension was stirred overnight, filtered, washed with distilled water, and dried in high vacuum to yield (**7**) in sufficient purity for further reactions. *R_f_* = 0.28 (cyclohexane/ethyl acetate 1:1). ^1^H NMR (400 MHz, DMSO-*d*_6_): δ = 12.51 (s, 1H), 10.22 (s, 1H), 8.36 (s, 1H), 7.75 (dd, 1H, *J* = 8.1, 1.0 Hz), 7.63 (dd, 1H, *J* = 7.5, 1.0 Hz), 7.43–7.46 (m, 2H), 7.31–7.40 (m, 4H), 5.37 (s, 2H). ^13^C NMR (101 MHz, DMSO-*d*_6_): δ = 186.3, 168.1, 137.9, 136.9, 136.1, 128.5, 128.3, 128.1, 124.6, 123.3, 122.6, 121.4, 118.2, 116.6, 66.4. LRMS (ESI) *m*/*z* = 280 [M + H]^+^.

#### 4-Benzyl 1-(*tert*-Butyl)-3-formyl-1*H*-indole-1,4-dicarboxylate (**8**)

To a solution of (**7**) (1.00 g, 3.58 mmol) in acetonitrile (50 mL), di-*tert*-butyldicarbonate (1.17 g, 5.37 mmol) and 4-dimethylaminopyridine (8.75 mg, 0.072 mmol) were added subsequently and the mixture was stirred for 48 h at room temperature. The solvent was evaporated, and the crude product was purified by flash chromatography (cyclohexane/ethyl acetate 4:1) to give a pale-yellow oil which crystallized as a colorless solid upon storage at 4 °C (1.18 g, 3.11 mmol, 87%). *R_f_* = 0.32 (cyclohexane/ethyl acetate 4:1). ^1^H NMR (400 MHz, CDCl_3_): δ = 10.47 (s, 1H), 8.50 (dd, 1H, *J* = 8.4, 0.9 Hz), 8.38 (s, 1H), 7.93 (dd, 1H, *J* = 7.6, 1.0 Hz), 7.33–7.49 (m, 6H), 5.45 (s, 2H), 1.70 (s, 9H). ^13^C NMR (101 MHz, CDCl_3_): δ = 188.5, 167.6, 148.4, 136.9, 135.6, 134.0, 128.6, 128.5, 128.3, 126.3, 124.8, 124.6, 124.6, 121.5, 119.6, 86.0, 67.2, 28.0. LRMS (ESI) *m*/*z* = 380 [M + H]^+^.

#### 4-Benzyl 1-(*tert*-Butyl)-3-[(1,3-dithian-2-ylidene)methyl]-1*H*-indole-1,4-dicarboxylate (**9**)

A solution of 1,3-dithiane-2-diethylphosphonate [10.1021/ol025665f] (676 mg, 2.64 mmol) in dry tetrahydrofuran (30 mL) was cooled to −78 °C and a 1.6 M solution of *n*-butyllithium in hexane (1.65 mL, 2.64 mmol) was added dropwise over a period of 15 min. After stirring for 1 h at this temperature, a solution of (**8**) (1.00 g, 2.64 mmol) in dry tetrahydrofuran (5 mL) was added slowly within 10 min. The mixture was warmed to room temperature and stirring was continued for 90 min. Next, water (5 mL) was added carefully, and the resulting mixture was extracted with ethyl acetate (3 × 20 mL). The combined organic layers were washed with brine (2 × 50 mL), dried over Na_2_SO_4_, and the solvent was evaporated. After flash chromatography (cyclohexane/ethyl acetate 9:1), the desired product was obtained as a yellow oil (1.04 g, 2.16 mmol, 82%). *R_f_* = 0.41 (cyclohexane/ethyl acetate 4:1). ^1^H NMR (400 MHz, DMSO-*d*_6_): δ = 8.33 (dd, 1H, *J* = 8.4, 0.8 Hz), 7.91 (d, 1H, *J* = 1.0 Hz), 7.64 (dd, 1H, *J* = 7.5, 1.0 Hz), 7.33–7.50 (m, 6H), 6.98 (d, 1H, *J* = 1.0 Hz), 5.39 (s, 2H), 2.92–2.95 (m, 4H), 2.06–2.13 (m, 2H), 1.63 (s, 9H). ^13^C NMR (101 MHz, DMSO-*d*_6_): δ = 167.2, 148.5, 135.7, 135.0, 129.5, 128.6, 128.3, 128.3, 127.3, 125.9, 124.9, 124.6, 124.3, 119.7, 118.2, 115.1, 84.8, 66.7, 29.2, 28.7, 27.6, 23.8. LRMS (ESI) *m*/*z* = 482 [M + H]^+^.

#### 4-Benzyl 1-(*tert*-Butyl)-3-(2-ethoxy-2-oxoethyl)-1*H*-indole-1,4-dicarboxylate (**10**)

To a suspension of (**9**) (500.00 mg, 1.04 mmol) in dry ethanol (40 mL) silver(I)nitrate (389 mg, 2.29 mmol) was added and the resulting mixture was heated at 60 °C for 4 h. After cooling to room temperature, the suspension was filtered over Celite and the solid was washed with ethyl acetate (100 mL). The filtrate and the combined wash solutions were transferred to a separating funnel and washed with water (50 mL) and brine (50 mL), dried over Na_2_SO_4_, and the solvent was evaporated. After flash chromatography (cyclohexane/ethyl acetate 9:1), the desired product was obtained as a pale-yellow oil (442 mg, 1.01 mmol, 97%). *R_f_* = 0.49 (cyclohexane/ethyl acetate 4:1); ^1^H NMR (400 MHz, DMSO-*d*_6_): δ = 8.39 (dd, 1H, *J* = 8.3, 0.8 Hz), 7.81 (s, 1H), 7.74 (dd, 1H, *J* = 7.6, 1.0 Hz), 7.33–7.48 (m, 6H), 5.29 (s, 2H), 3.98 (quart, 2H, *J* = 7.1 Hz), 3.95 (s, 2H), 1.62 (s, 9H), 1.13 (t, 3H, *J* = 7.1 Hz). ^13^C NMR (101 MHz, DMSO-*d*_6_): δ = 171.2, 166.6, 148.6, 136.2, 135.9, 128.6, 128.3, 128.2, 128.1, 127.8, 125.0, 124.5, 123.7, 118.8, 113.7, 84.3, 66.4, 60.1, 32.8, 27.6, 14.1. LRMS (ESI) *m*/*z* = 438 [M + H]^+^.

#### 1-(*tert*-Butoxycarbonyl)-3-(2-ethoxy-2-oxoethyl)-1*H*-indole-4-carboxylic Acid (**11**)

To a solution of (**10**) (300 mg, 0.69 mmol) in dry ethanol (15 mL), ammonium formate (865 mg, 13.72 mmol) and Pd(0) (10% on C, 22.60 mg, 0.021 mmol) were added and positive pressure was applied by fitting a nitrogen-filled balloon on the apparatus. The resulting mixture was heated to reflux for 3 h. After cooling to room temperature, the suspension was filtered over Celite and the solid was washed with ethyl acetate (100 mL). The volatile components of the filtrate and the combined washing solutions were evaporat*e*d *in vacuo*, and the residue was redissolved in ethyl acetate (100 mL) and washed with water (50 mL) and brine (50 mL) subsequently, dried over Na_2_SO_4_, and the solvent was evaporated. After flash chromatography (cyclohexane/ethyl acetate 7:3), the desired product was obtained as a colorless solid (188 mg, 0.54 mmol, 79%). *R_f_* = 0.57 (cyclohexane/ethyl acetate 1:1). ^1^H NMR (400 MHz, DMSO-*d*_6_): δ = 12.96 (s, 1H), 8.35 (dd, 1H, *J* = 8.3, 0.8 Hz), 7.77 (s, 1H), 7.70 (dd, 1H, *J* = 7.6, 1.0 Hz), 7.39 (t, 1H, *J* = 8.0 Hz), 4.01 (quart, 2H, *J* = 7.1 Hz), 3.96 (s, 2H), 1.63 (s, 9H), 1.15 (t, 3H, *J* = 7.1 Hz). ^13^C NMR (101 MHz, DMSO-*d*_6_): δ = 171.3, 168.5, 148.7, 136.2, 128.1, 127.9, 125.9, 124.9, 123.6, 118.3, 114.0, 84.2, 60.0, 33.0, 27.7, 14.1. LRMS (ESI) *m*/*z* = 348 [M + H]^+^.

#### 3-(2-Ethoxy-2-oxoethyl)-1*H*-indole-4-carboxylic Acid (**12**)

To a solution of (**11**) (419 mg, 1.21 mmol) in dry dichloromethane (12 mL), trifluoroacetic acid (5.00 mL, 7.40 g, 64.9 mmol) was added and the resulting mixture was stirred for 3 h at room temperature. Then, the mixture was poured into ice-cold water (100 mL) carefully and the resulting solution was extracted with ethyl acetate (100 mL) which was washed with water (50 mL) and brine (50 mL) subsequently, dried over Na_2_SO_4_, and the solvent was evaporated. After flash chromatography (cyclohexane/ethyl acetate 3:2), the desired product was obtained as an off-white solid (225.00 mg, 0.91 mmol, 75%). *R_f_* = 0.41 (cyclohexane/ethyl acetate 1:1). ^1^H NMR (400 MHz, DMSO-*d*_6_): δ = 12.54 (br. s, 1H), 11.29 (s, 1H), 7.54–7.58 (m, 2H), 7.36 (d, 1H, *J* = 2.2 Hz), 7.12 (t, 1H, *J* = 7.7 Hz), 4.00 (quart, 2H, *J* = 7.1 Hz), 3.95 (s, 2H), 1.15 (t, 3H, *J* = 7.1 Hz). ^13^C NMR (101 MHz, DMSO-*d*_6_): δ = 172.3, 169.6, 137.9, 127.7, 124.8, 122.0, 119.9, 115.7, 108.2, 59.6, 33.3, 14.2. HRMS (ESI) *m*/*z* calc. for C_13_H_14_NO_4_ [M + H]^+^: 248.09173, found: 248.09158.

#### General Procedure for Coupling Reactions to *N*-Methyl Amides (**3a**–**p**)

To a stirred solution of the carboxylic acid (**12**) (50.00 mg, 0.20 mmol) in DMF (3 mL), HATU (152 mg, 0.40 mmol), the *N*-methylamine component (0.24 mmol), and DIPEA (102.04 μL, 77.5 mg, 0.60 mmol) were added and the resulting mixture was stirred for 18 h at room temperature. Then, the reaction mixture was poured into water (20 mL) and the aqueous phase was extracted with ethyl acetate (3 × 10 mL), which was afterward washed with 1 M HCl solution (20 mL), saturated NaHCO_3_ solution (20 mL) and brine (20 mL) subsequently, dried over Na_2_SO_4_, and the solvent was evaporated. After flash chromatography (cyclohexane/ethyl acetate, linear gradient from 100:0 to 40:70 within 15 min) followed by reversed-phase flash chromatography (water/acetonitrile, linear gradient 65:35 to 3:97 within 12 min), the products were obtained in sufficiently high purity. NMR analyses showed that the products **3a**–**p** occur as two rotamers.

#### Ethyl 2-{4-[Benzyl(methyl)carbamoyl]-1*H*-indol-3-yl}acetate (**3a**)

The title compound was produced by following the general procedure using *N*-methyl-1-phenylmethanamine (29 mg) to yield **3a** as a yellow oil (63.5 mg, 0.18 mmol, 91%). *R*_f_ = 0.28 (cyclohexane/ethyl acetate 1:1). ^1^H NMR (400 MHz, DMSO-*d*_6_): δ = 11.23 (s, 1H minor isomer), 11.22 (s, 1H major isomer), 7.44–7.11 (m, 7H minor isomer, 8H major isomer), 7.04–7.00 (m, 1H minor isomer), 6.91–6.89 (m, 1H minor isomer, 1H major isomer), 4.67 (br. s, 2H major isomer), 4.36 (br. s, 1H minor isomer), 4.06–4.36 (m, 4H minor isomer), 4.02 (quart, 2H, *J* = 7.0 Hz, major isomer), 3.69 (s, 2H minor isomer), 3.66 (s, 2H major isomer), 2.90 (s, 3H minor isomer), 2.69 (s, 3H major isomer), 1.21 (t, 3H, *J* = 7.0 Hz, minor isomer), 1.18 (t, 3H, *J* = 7.1 Hz, major isomer). ^13^C NMR (101 MHz, DMSO-*d*_6_): δ = 171.7, 171.7, 170.7, 170.4, 137.5, 137.0, 136.9, 136.8, 128.7, 128.6, 127.9, 127.7, 127.3, 127.2, 126.8, 126.2, 125.9, 123.1, 120.5, 120.4, 116.9, 116.3, 112.5, 112.4, 106.9, 106.8, 60.1, 60.0, 49.5, 36.7, 32.2, 30.9, 30.8, 14.2, 14,2. HRMS (ESI) *m*/*z* calc. for C_27_H_27_N_2_O_3_ [M + H]^+^: 351.17032, found: 351.16995.

#### Ethyl 2-{4-[(1,1′-Biphenyl-4-ylmethyl)(methyl)carbamoyl]-1*H*-indol-3-yl}acetate (**3n**)

The title compound was produced by following the general procedure using 1-(1,1′-biphenyl-4-yl)-*N*-methylmethanamine (47.35 mg) to yield **3n** as a yellow oil (36.7 mg, 0.086 mmol, 43%). *R_f_* = 0.27 (cyclohexane/ethyl acetate 1:1). ^1^H NMR (400 MHz, DMSO-*d*_6_): δ = 11.26 (s, 1H minor isomer), 11.23 (s, 1H major isomer), 7.64–7.70 (m, 4H minor isomer, 4H major isomer), 7.23–7.51 (m, 7H minor isomer, 7H major isomer), 7.12–7.16 (m, 1H major isomer), 7.03–7.05 (m, 1H minor isomer), 6.92–6.95 (m, 1H major isomer, 1H minor isomer), 4.72 (br. s, 2H major isomer), 4.42 (br. s, 1H minor isomer), 4.18 (br. s, 1H minor isomer), 4.11 (quart, 2H, *J* = 7.1 Hz, minor isomer), 4.01 (quart, 2H, *J* = 7.1 Hz, major isomer), 3.71 (s, 2H minor isomer), 3.67 (s, 2H major isomer), 2.94 (s, 3H minor isomer), 2.74 (s, 3H major isomer), 1.22 (t, 3H, *J* = 7.1 Hz, minor isomer), 1.15 (t, 3H, *J* = 7.1 Hz, major isomer). ^13^C NMR (101 MHz, DMSO-*d*_6_): δ = 171.7, 171.7, 170.8, 170.4, 139.9, 139.7, 139.2, 139.1, 136.9, 136.8, 136.8, 136.2, 129.0, 128.6, 127.9, 127.7, 127.5, 127.4, 127.0, 126.9, 126.6, 126.2, 125.9, 123.1, 123.1, 120.5, 120.4, 116.9, 116.3, 112.5, 112.4, 107.0, 106.8, 60.1, 60.0, 53.9, 49.3, 36.8, 32.2, 30.9, 30.9, 14.2, 14.1. HRMS (ESI) *m*/*z* calc. for C_27_H_27_N_2_O_3_ [M + H]^+^: 427.20162, found: 427.20150.

### Pharmacophore Models and Virtual Screening

The pharmacophore models were initially built based on the crystal structure of the 4-phenyl (IUCr reference: XU5576)^62^ instead of 4-biphenyl-substituted **ITD-1** (**1a**) analogue in MOE (Molecular Operating Environment 2012, 2010.10, Chemical Computing Group, Inc., Montreal, Canada), which was extracted from the Cambridge Structural Database (ConQuest V1.16.0).^63^ The physicochemical properties of the pharmacophore models (hydrophobic or aromatic region, H-bond donor or acceptor) were placed according to our SAR knowledge at the time within either the ligand or resembling the binding partner counterpart to define, *e.g*., the directionality of H-bonds. For virtual screening, we generated a conformer database of the ZINC lead-like Database containing 7.61 mio. substances.^64^ To do this, we restricted the number of conformers to 500 with a maximal free energy of 4.5 kcal/mol. Hits were selected with an RMSD < 0.83 Å. The validness of the pharmacophore model could be confirmed with the later obtained crystal structure of ITD-1.^37^

### Biology

#### Cell Lines and Cell Culture

All cell lines were purchased from American Type Culture Collection (ATCC). Media was supplemented with 10% fetal bovine serum (FBS) and maintained at 37 °C in a 5% CO_2_ atmosphere. A549 cells were maintained in F12K media (Gibco, Waltham), H1299 NSCLC cells were maintained in RPMI-1640 media (Corning, New York), Rat2 and HEK293T cells were maintained in Dulbecco’s modified Eagle’s media (DMEM) (Gibco, Waltham), and Mv1Lu cells were maintained in MEM media (Gibco, Waltham).

#### SBE4*-luc* Reporter Gene Assay

The assay was performed as previously described.^36^ Briefly, HEK293T cells were batch-transfected with a SBE4-*luc* plasmid (Promega, Madison) using Lipofectamine 2000 (Invitrogen, Waltham) in 2% DMEM high-glucose medium, incubated for 12–14 h, and subsequently plated on a 96-well plate (25000/well) for 2 h in 1% DMEM high-glucose medium before treatment with TGFβ2 (10 ng/mL, Peprotech Germany, Hamburg, Germany) and inhibitors (0.001–10 μM) or DMSO for 20–22 h. Cells were lysed using Promega cell lysis buffer, and luminescence readouts were performed using the Dual Luciferase Assay Kit (Promega, Madison) on a TECAN Spark plate reader (Tecan Group, Männedorf, Switzerland).

#### Immunoblotting

Cells were washed with 1× phosphate-buffered saline (PBS), lysed in lysis buffer (50 mM Tris-HCl, pH 7.5, 150 mM NaCl, 1 mM ethylenediamine tetraacetic acid (EDTA), 0.5% Triton X-100, and a 1:1 mixture of 1 mM phenylmethylsulfonyl fluoride (PMSF) and 1 mM Pepstatin A solution), and centrifuged (21 000*g*, 4 °C, 10 min). Protein concentrations were measured using the DC protein assay kit (Bio-Rad Laboratories, Inc., Hercules, 5000112). Lysates were prepared and separated in 12% sodium dodecyl sulfate polyacrylamide gel electrophoresis (SDS-PAGE) gels. Proteins were transferred to nitrocellulose membranes and blocked with 5% skim milk at room temperature for 1 h. Membranes were exposed to the following primary antibodies overnight, at 4 °C: anti-pSmad2 (Cell Signaling, 3108L), anti-Smad2,3 (BD Biosciences, 610843), anti-GAPDH (Cell Signaling, 2118S), anti-E-cadherin (BD Biosciences, 610182), anti-N-cadherin (BD Biosciences, 610921), anti-Snail (Cell Signaling, 3879S), anti-TβRI (Invitrogen, PA5-78198), and anti-TβRII (Santa Cruz Biotechnology, sc-17799, 1:500). All primary antibodies were used at 1:1000 dilution, unless specified otherwise. The next day, membranes were washed with Tris-buffered saline with added Tween-20 (TBST) and exposed to either goat-antirabbit or goat-antimouse HRP-conjugated secondary antibodies for 1 h at room temperature. Membranes were washed with TBST and imaged via Clarity Max Western ECL Substrate (Bio-Rad, Hercules, 1705062) and a Versa-doc Imager (Bio-Rad, Hercules). Densitometry was performed using QuantityOne 1-D analysis software. To image TGFβ receptors, cells were treated with inhibitors for 24 h and then TGFβ1 for 1 h. To follow the expression of epithelial or mesenchymal markers, the cells were treated with inhibitors and TGFβ1 for 48 h.

#### Receptor Internalization Assay

Mv1Lu cells constitutively expressing extracellularly HA-tagged TβRII were plated on coverslips and treated with inhibitors or DMSO for 22 h in 0.2% FBS media. For antibody labeling, the treated cells were stored on ice for 10 min, washed with ice-cold 1× PBS twice, and incubated with an anti-HA antibody (Santa Cruz Biotechnology, SC-57592) for 2 h at 4 °C. Next, the cells were washed 3× with cold PBS to reduce background noise, followed by an incubation with Alexa Fluor 568 (Invitrogen, A**-**21134) for 1 h at 4 °C. After antibody labeling, all samples were washed 3× with cold PBS. Internalization was done by incubating the cells with fresh media containing 10% FBS for 1 h at 37 °C. After the internalization, the cells were washed 5× with 37 °C PBS and fixed using 4% PFA with added Hoechst33342 (1:1000) for 10 min. All samples were mounted on glass slides and imaged using an Olympus CKX53 fluorescence microscope (Olympus K.K., Shinjuku, Japan) at 40× magnification.

#### Stress Fiber Formation Assay

Rat2 fibroblasts or A549 NSCLC cells were plated on glass coverslips and fixed with 4% PFA for 10 min, permeabilized using 0.25% Triton X-100 for 5 min, and then blocked (1 h, RT) with 10% FBS in PBS. A549 cells were stained with Alexa Fluor-555 Phalloidin (1:400) for 1 h (RT) to visualize filamentous actin. Rat2 cells were additionally stained with primary anti-Tubulin (1:200) overnight at 4 °C to visualize microtubules and co-stained with an Alexa Fluor-488 secondary antibody (1:350) at RT for 1 h. DAPI was used to stain the nuclei of both cell lines. Coverslips were mounted onto glass slides using Immuno-Mount solution (Thermo Scientific, Waltham, 9990402), and cells were imaged using an Olympus IX 81 inverted fluorescence microscope (Olympus K.K., Shinjuku, Japan) at 40×.

#### Wound Healing (Scrape) Assay

H1299 NSCLC cells were serum-starved overnight and then treated with 1 μM **1a**, 5 μM **2r**, or 5 μM **3n**, in the absence or presence of 100 pM TGFβ1 for 24 h. The monolayers of cells were then scraped with a pipet tip, and cells were washed with 1× PBS twice to remove any debris or floating cells from the scrape. Fresh media was added back to the cells, and using a Leica DMI6000 B microscope (Leica Microsystems, Wetzlar, Germany), images were taken immediately after, which represented the 0 h time point. Compounds and TGFβ1 were added back into the media in respective wells and were incubated with the cells for an additional 24 h. Images of the scrape were taken at 6, 12, and 24 h time points in the same location using the Mark & Find function in the Leica Application Suite X (Las X, v. 3.3.3.16958) program. The MRI wound healing size tool plugin for ImageJ (Volker Bäcker) was used to analyze the area of the scratch. Wound closure percentage was calculated by the following formula: ((*A*_*t*=0_ – *A*_*t*=Δ*t*_)/*A*_*t*=0_) × 100%.

#### Statistical Analysis

Two-way analysis of variance (ANOVA) followed by a post hoc Bonferroni test was used to analyze the significance of results. Analysis was carried out using GraphPad Prism (v. 10.0.2) and *p*-values ≤0.05 were deemed statistically significant. All experiments are composed of at least three individual repetitions.

## Supporting Information Available

TGFβ inhibition screen (Figure S1), Western blotting data in absence of TGFβ for (phospho)Smad2 (Figure S2), TβRI and II (Figure S3A) and EMT-marker (Figure S3B); stress fiber formation for A549 cells (Figure S4) and Rat2 fibroblasts, the latter including microtubule network assessment (Figure S5); separation of (+)- and (−)-enantiomers of **1b** (Figure S6) and single-crystal X-ray structure of (−)-**1b** (Figures S6 and S7, Table S1); extended experiments for the synthesis of **3a**–**p**; spectroscopic data for all synthesized compounds, including ^1^H and ^13^C NMR spectra; and HPLC traces of all final test compounds (PDF)

## Abbreviations

AF555: Alexa Fluor 555
AF568: Alexa Fluor 568
Akt: protein kinase b
α PKC: protein kinase c α
DAPI: 4′,6-diamidino-2-phenylindole
DMEM: Dulbecco’s modified Eagle’s medium
EMT: epithelial–mesenchymal transition
F12K: Ham’s F-12 Kaighn’s medium
F-actin: filamentous actin
FBS: fetal bovine serum
GAPDH: glyceraldehyde 3-phosphate dehydrogenase
HATU: hexafluorophosphate azabenzotriazole tetramethyluronium
HRP: horse radish peroxidase
HSP90: heat shock protein 90
HWE: Horner–Wadsworth–Emmons
MAPK: mitogen-activated protein kinase
NSCLC: non-small-cell lung cancer
Par6: partitioning defective 6 homologue
PDD: phenotypic drug discovery
PFA: paraformaldehyde
PI3K: phosphoinositol-3 kinase
PMSF: phenylmethylsulfonyl fluoride
RhoA: ras homologue family member A
RPMI-1640: Roswell Park Memorial Institute 1640
SBE4-*luc*: Smad binding element 4-firefly luciferase
Smad: combination of small body size and mothers against decapentaplegic
Smurf-2: Smad specific E3 ubiquitin protein ligase 2
TβRI: transforming growth factor-β receptor type I
TβRII: transforming growth factor-β receptor type II
TBST: Tris-buffered saline with Tween 20
TGFβ: transforming growth factor-β
