# Supplementary material for: Expanding the Chemical Space of Transforming Growth Factor-β (TGFβ) Receptor Type II Degraders with 3,4-Disubstituted Indole Derivatives
Source: ACS Pharmacol Transl Sci. 2024 Mar 21;7(4):1069–85. doi: 10.1021/acsptsci.3c00371 (PMC11020067; doi:10.1021/acsptsci.3c00371)
Supplement: Supplementary file 1 — pt3c00371_si_001.pdf [file pt3c00371_si_001.pdf]

ACS Pharmacology & Translational Science

Supporting Information

Expanding the chemical space of Transforming growth factor β (TGFβ) receptor type II degraders with 3,4-disubstituted indole derivatives

Daniel Längle,[1,#] Stephanie Wojtowicz-Piotrowski,[2,#] Till Priegann,[1,#] Niklas Keller,[1] Fabian Wesseler,[1,3] Elena S. Reckzeh,[3,§] Karsten Steffens,[1] Christoph Grathwol,[4] Jana Lemke,[4] Maren Flasshoff,[3] Christian Näther,[5] Anna C. Jonson,[6] Andreas Link,[4] Oliver Koch,[3,7] Gianni M. Di Guglielmo,[2*] Dennis Schade[1,8*]

[1] Department of Pharmaceutical & Medicinal Chemistry, Christian-Albrechts-University of Kiel, Gutenbergstrasse 76, 24118 Kiel, Germany;
[2] Department of Physiology and Pharmacology, Schulich School of Medicine and Dentistry, Western University, London, ON, Canada;
[3] Faculty of Chemistry and Chemical Biology, Technical University Dortmund, Otto-Hahn-Strasse 6, 44227 Dortmund, Germany;
[4] Institute of Pharmacy, University of Greifswald, Friedrich-Ludwig-Jahn-Strasse 17, 17489 Greifswald, Germany;
[5] Institute of Inorganic Chemistry, Christian-Albrechts-University of Kiel, Max-Eyth-Straße 2, 24118 Kiel, Germany
[6] Early Product Development, Pharmaceutical Sciences, IMED Biotech Unit, AstraZeneca Gothenburg, Mölndal SE-43183, Sweden
[7] Institute of Pharmaceutical and Medicinal Chemistry and German Center of Infection Research, Münster, Germany.
[8] Partner Site Kiel, DZHK, German Center for Cardiovascular Research, 24105 Kiel, Germany
[#] equal contribution
[§] Current address: University of Bonn, TRA „Life and Health“, LIMES Institute, Department Organoids & Chemical Biology, Carl-Troll-Str. 31, 53115 Bonn, Germany
** Lead contacts: schade@pharmazie.uni-kiel.de, john.diguglielmo@schulich.uwo.ca*

Table of Contents

Supplemental Figures 2
- Figure S1 2
- Figure S2 3
- Figure S3 4
- Figure S4 5
- Figure S5 8
- Table S1 9

Extended Chemistry 10
- Synthetic procedures for indol-3-acetates (**3**) 10
- ^{1}H and ^{13}C NMR spectra of newly synthesized compounds 15
- HPLC chromatograms of **2p,r** and **3a-p** 37
- Single crystal structure analysis of **(−)-1b** 43

Supplemental References 43

Supplemental Figures

Figure S1

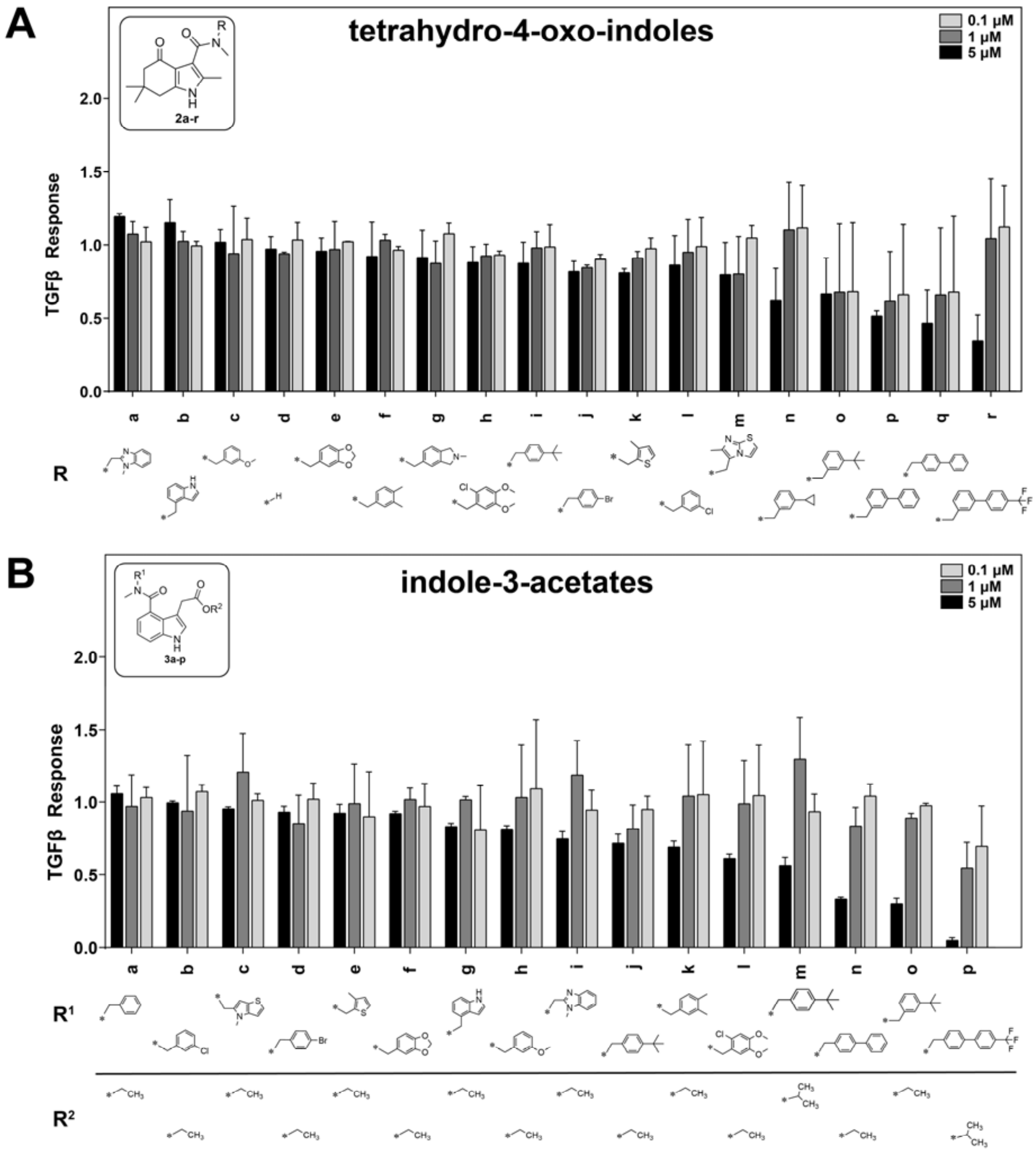

Figure S1 │TGFβ inhibition screen of amide libraries 2a-r (A) and 3a-p (B). Data from a SBE4-*luc* assay in HEK293T cells, *n* = 2-3 independent experiments (mean ± SD, normalized to DMSO = 1).

Figure S2

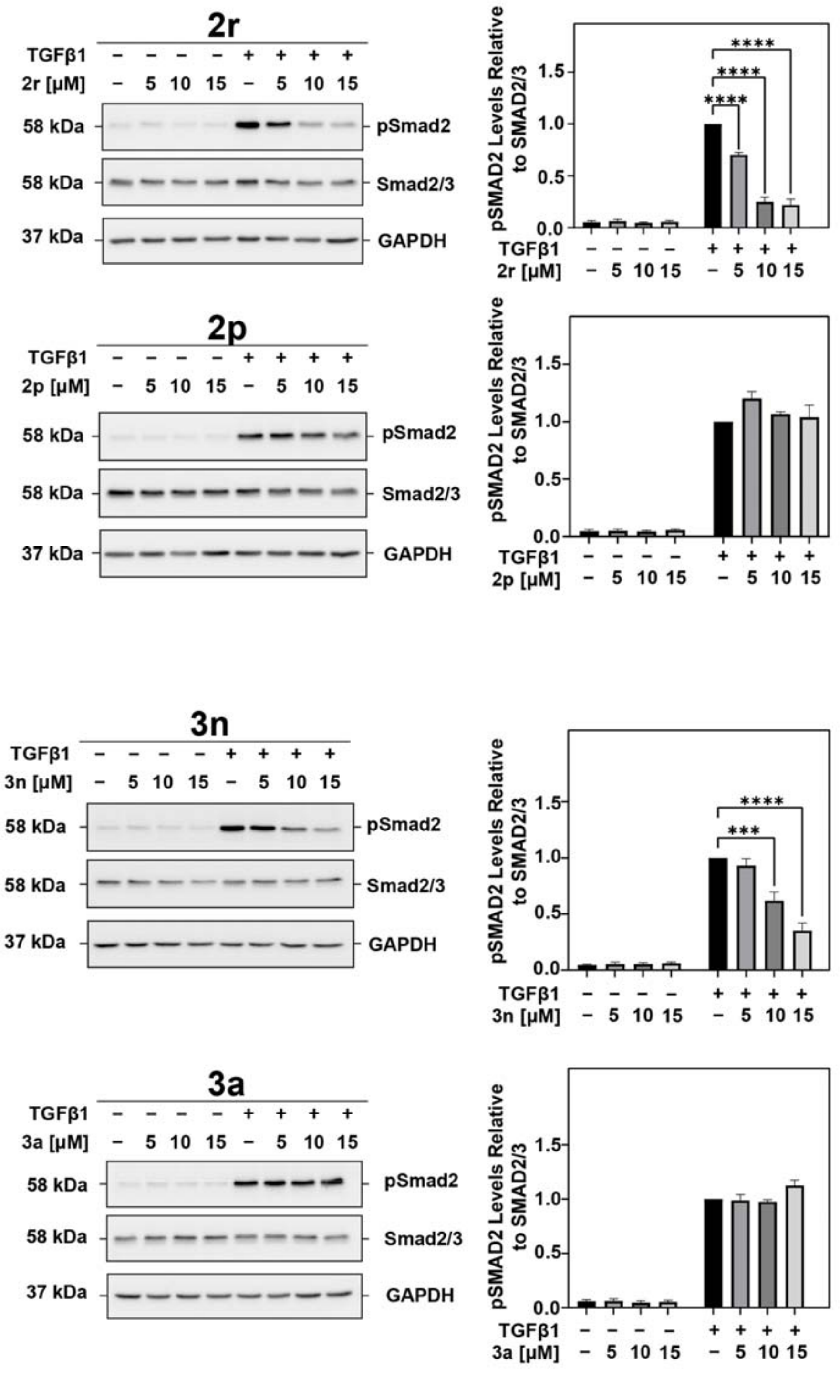

Figure S2 │**Supplementary Western Blotting data for (phospho)Smad2 in presence and absence of TGFβ.** A549 cells were serum-starved and treated with increasing concentrations of **2r, 2p, 3a,** and **3n** for 24 hours, followed by TGFβ1 treatment (100 pM, 1 h). Protein lysates were subjected to Western blotting for pSmad2, Smad2/3, and GAPDH. Relative levels of the protein of interest were quantitated using QuantityOne software and graphed as the ratio of pSmad2/ Smad2/3; *n* = 3 independent experiments (mean ± SEM, normalized to TGFβ1/DMSO = 1. *** $p \leq 0.005$, **** $p \leq 0.0001$)**.**

Figure S3

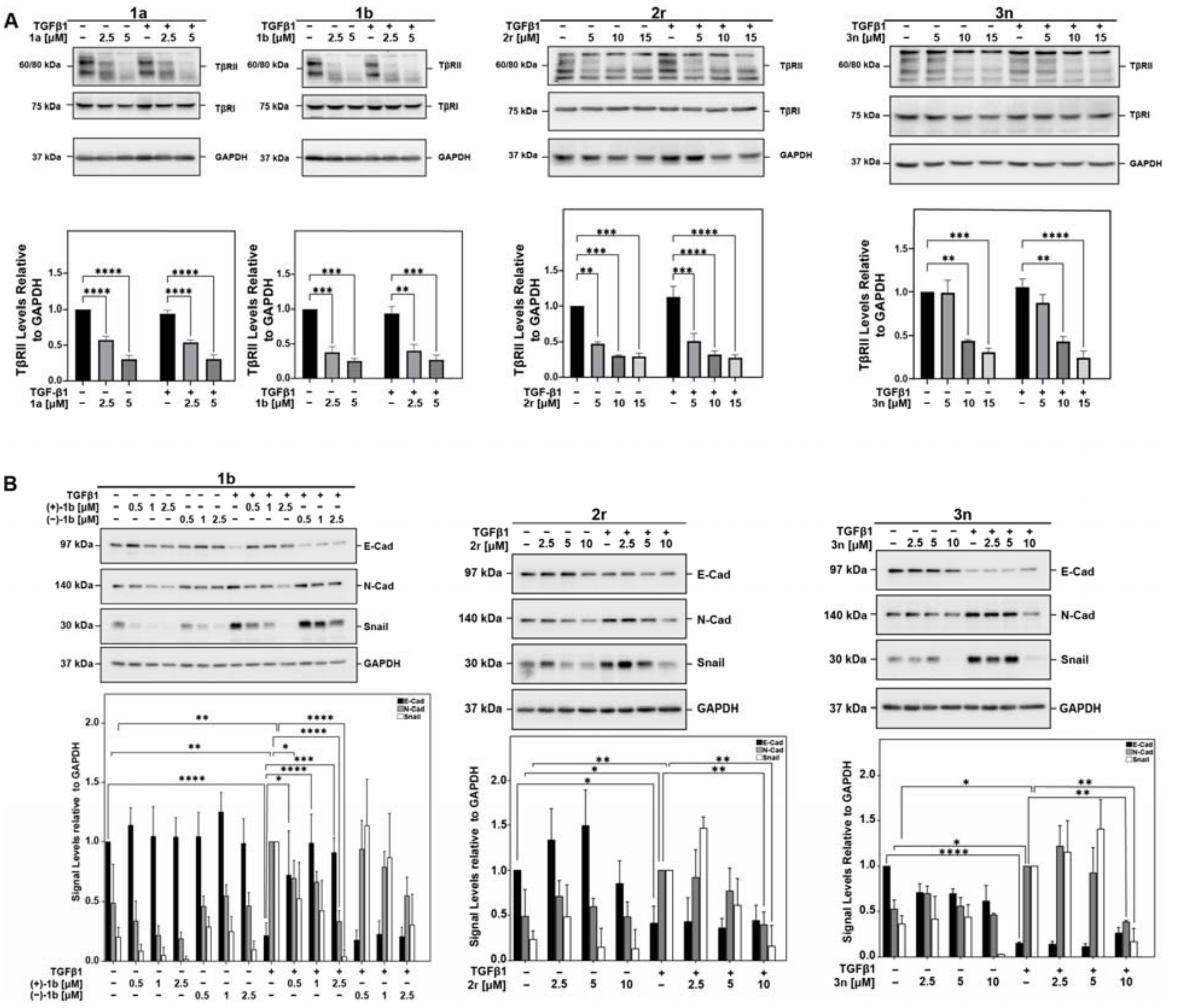

Figure S3 | Supplementary data for TβRII degradation and EMT inhibition in presence and absence of TGFβ. (A) A549 cells were serum-starved, then treated with the indicated compounds for 24 h, followed by TGFβ-1 (100 pM, 1 h). Protein lysates were subjected to Western blotting for TβRI, TβRII and GAPDH. Relative levels of the protein of interest were quantitated using QuantityOne software and graphed as the ratio of TβRII/GAPDH. n = 3-5 independent experiments (mean ± SEM, normalized to DMSO = 1, ** $p \leq 0.01$, *** $p \leq 0.001$; **** $p \leq 0.0001$). **(B)** A549 cells were serum-starved, then treated with increasing concentrations of (+)-**1b,** (−)-**1b, 2r,** or **3n** and TGFβ1 (100 pM, 48 h). Protein lysates were subjected to Western blotting for E-cadherin (E-cad), N-cadherin (N-cad), and Snail levels. Relative levels of the protein of interest were quantitated using QuantityOne software and graphed as the ratio of protein of interest/GAPDH. n = 3 independent experiments (mean ± SEM, normalized to DMSO for E-Cad and normalized to TGFβ1/DMSO for N-Cad and Snail, * $p \leq 0.05$, ** $p \leq 0.01$, *** $p \leq 0.001$; **** $p \leq 0.0001$).

Figure S4

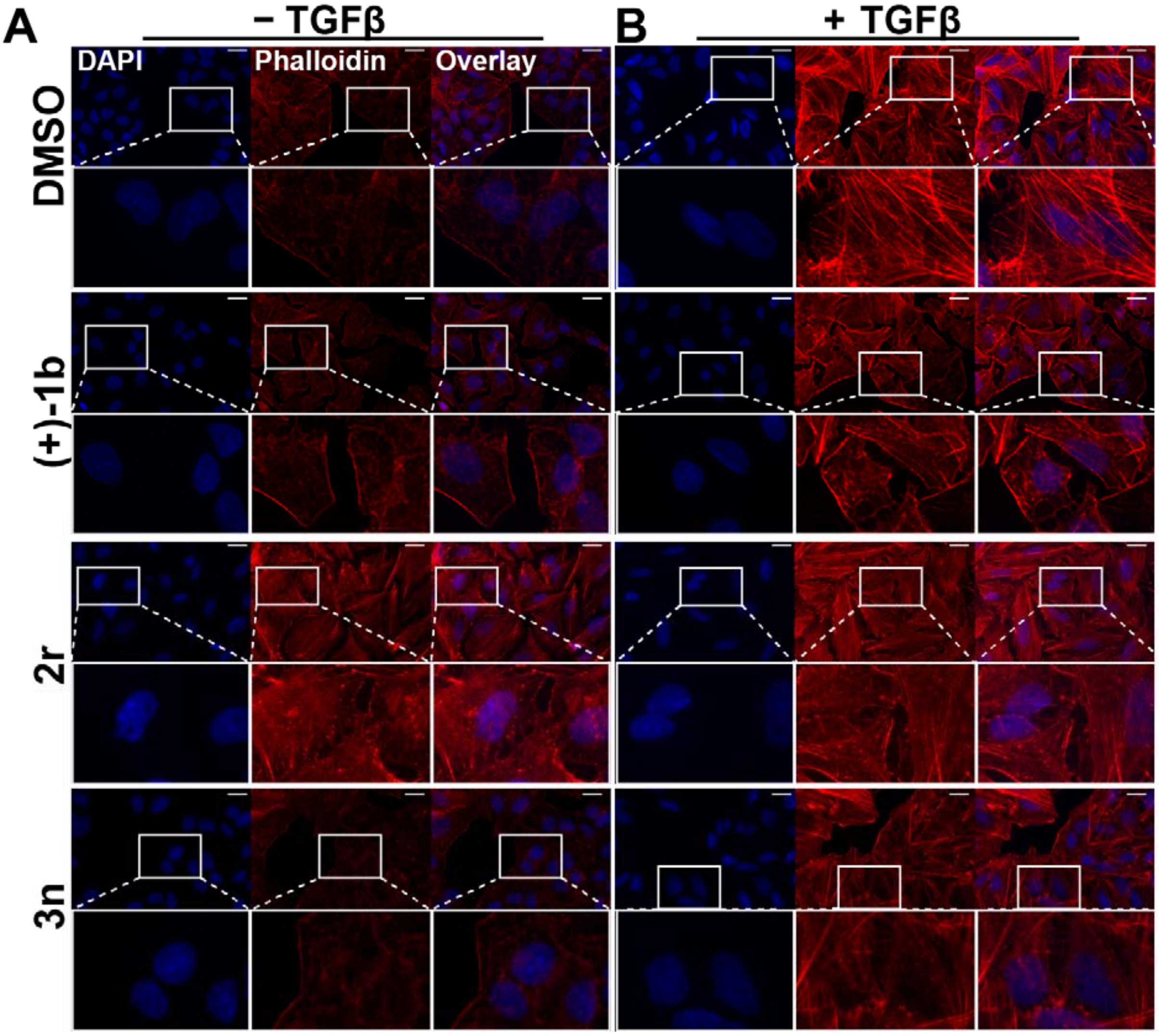

Figure S4 | Supplementary data on TβRII degrader chemotypes 1, 2 and 3 to modulate stress fiber formation in A549 cells **(A, B)** A549 cells were serum-starved, then treated with the indicated compounds (1 µM **1b**, 5 µM **2r** and **3n**) **(A)** and 100 pM TGFβ1 **(B)** for 48 h. Cells were stained with DAPI (nuclei, blue) and AF555-Phalloidin (red) to visualize cortical actin in control cells or stress fibers in TGFβ-treated cells (Olympus IX 81 microscope, 40x, scale bar = 20 µm).

Figure S5

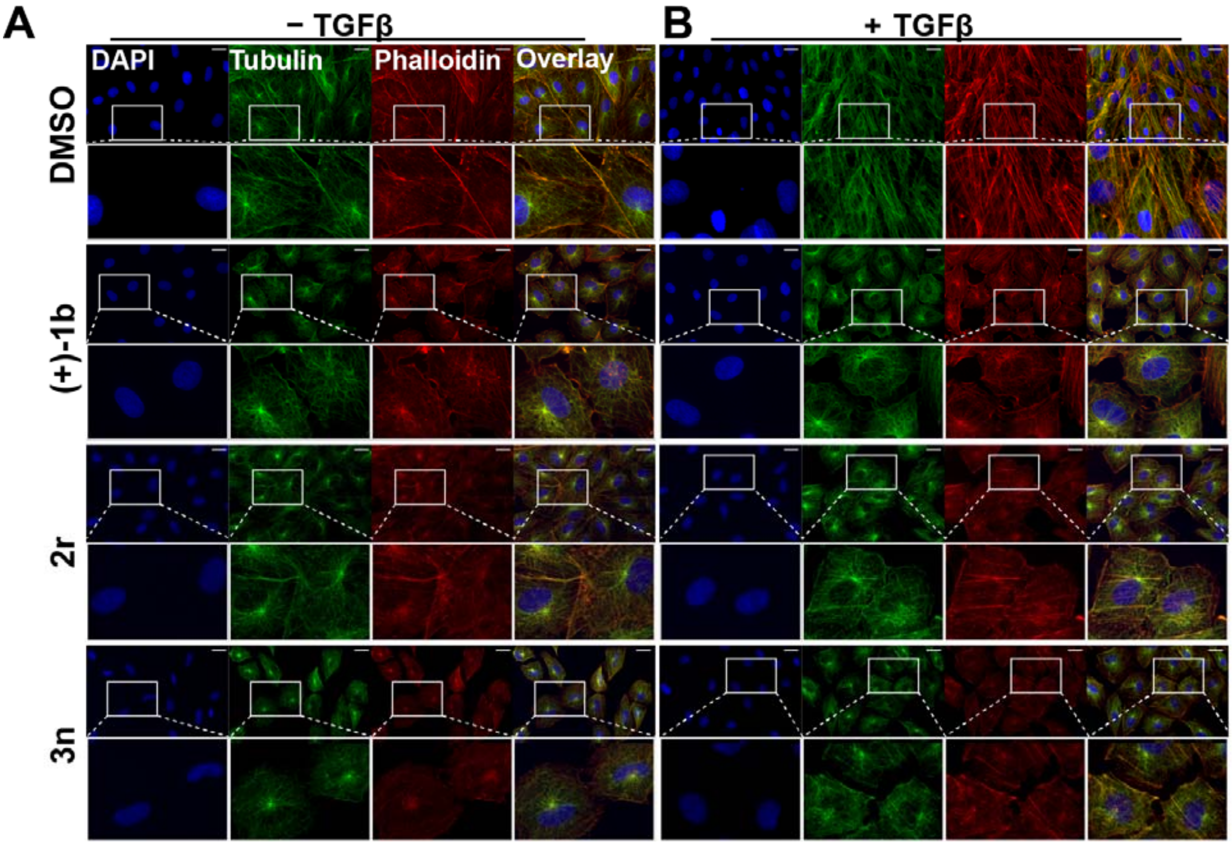

Figure S5 | Supplementary data on TβRII degrader chemotypes 1, 2 and 3 to modulate stress fiber formation and microtubules in Rat2 fibroblasts. (**A, B)** Rat2 fibroblasts were serum-starved and treated with the indicated compounds (1 µM **1b**, 5 µM **2r** and **3n**) **(A)** and TGFβ **(B)** for 48 h. Cells were immunostained against tubulin (green) and co-stained with AF555-Phalloidin (F-actin, red), and DAPI (nuclei, blue) (Olympus IX 81 microscope, 40x, scale bar = 20 µm).

Figure S6

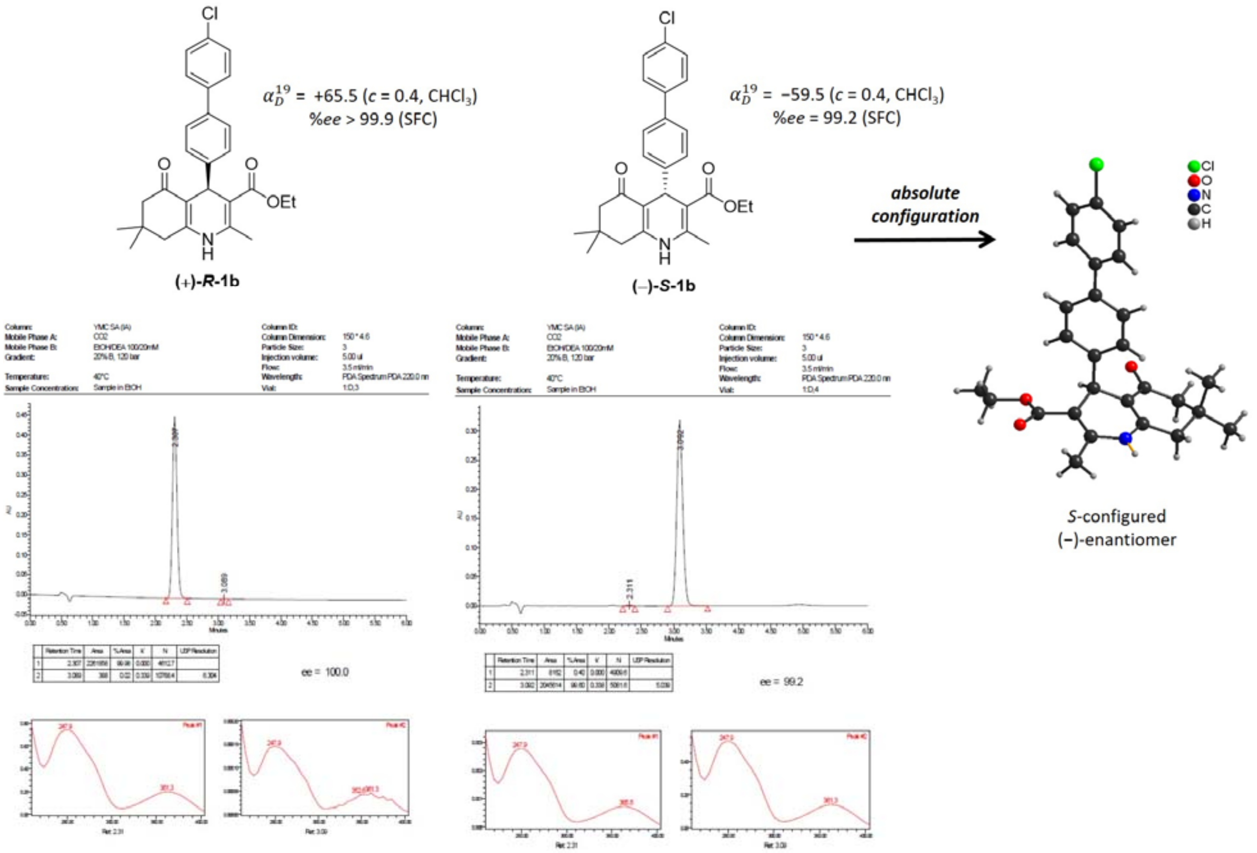

Figure S6 | Supplementary data for the resolution of 1b enantiomers and x-ray crystallography of (−)-1b. Analytical SFC chromatogram (chiral stationary phase: YMC SA (IA)) of the single isolated enantiomers for the determination of %*ee*, along with their optical rotation values and the assignment of absolute configuration by single crystal x-ray analysis of (−)-**1b**.

Figure S7

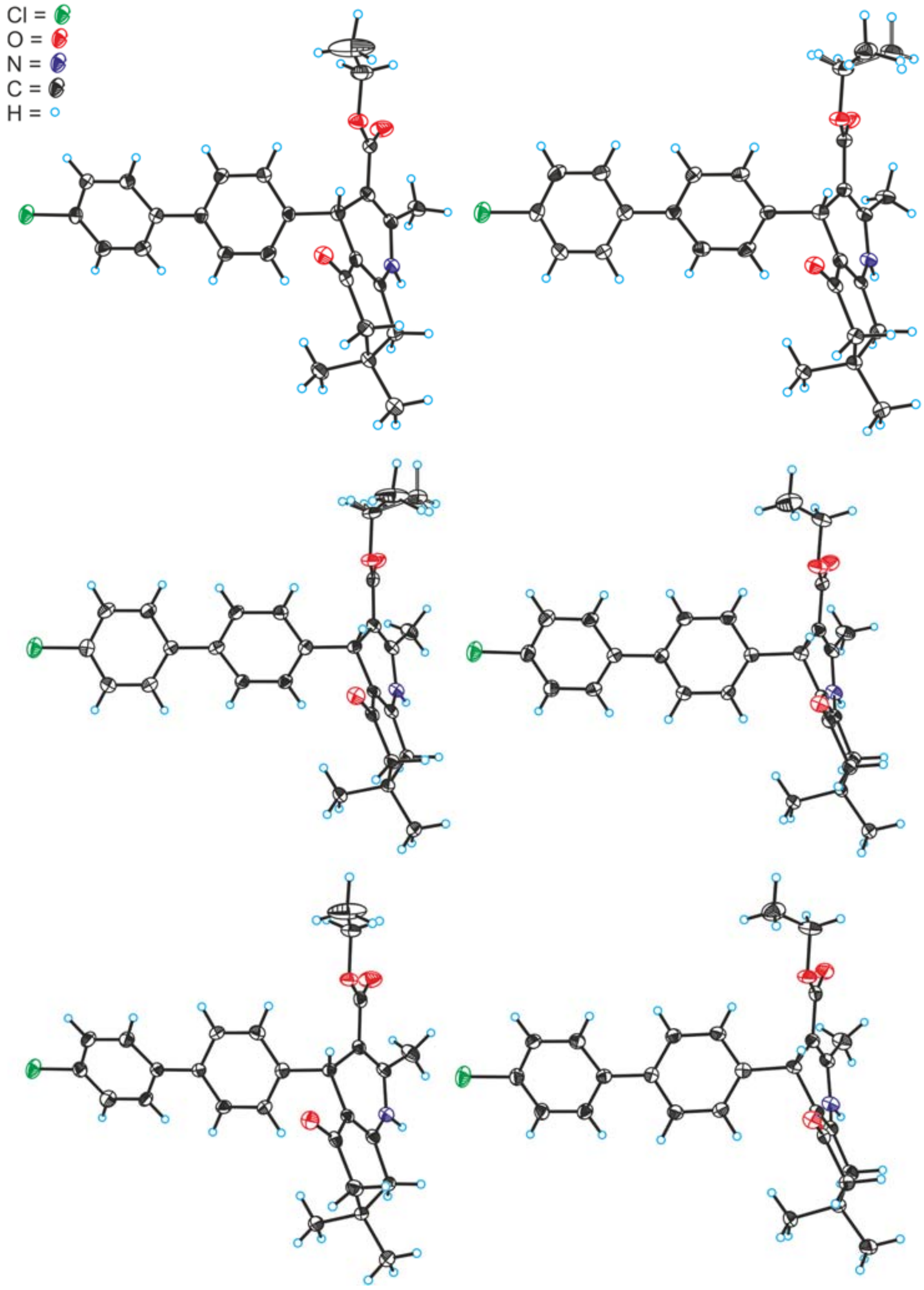

Figure S7 | ORTEP diagram for (−)-1b. Crystal structure of the six crystallographically independent molecules with displacement ellipsoids drawn at the 50% probability level. Please note that in two of these molecules one methyl group is disordered.

Table S1

Table S1 | Selected crystal data and details of the structure refinement for (–)-1b

Empirical formula	$C_{27}H_{28}ClNO_3$
Formula weight	449.95
Temperature/K	100.00(10)
Crystal system	monoclinic
Space group	$P2_1$
a/Å	11.37640(10)
b/Å	26.9728(5)
c/Å	22.4357(2)
α/°	90
β/°	90.009(3)
γ/°	90
Volume/Å^3	6884.47(15)
Z	12
ρ_{calc}g/cm^3	1.302
μ/mm^{-1}	1.703
F(000)	2856.0
Crystal size/mm^3	0.22 × 0.16 × 0.14
Radiation	Cu Kα (λ = 1.54178)
2Θ range for data collection/°	5.124 to 160.822
Index ranges	-14 ≤ h ≤ 14, -34 ≤ k ≤ 33, -28 ≤ l ≤ 28
Reflections collected	102742
Independent reflections	28631 [R_{int} = 0.0241, R_{sigma} = 0.0176]
Reflections with [I>=2σ (I)]	28394
Data/restraints/parameters	28631/9/1800
Goodness-of-fit on F^2	1.072
Final R indexes [I>=2σ (I)]	R_1 = 0.0299, wR_2 = 0.0851
Final R indexes [all data]	R_1 = 0.0301, wR_2 = 0.0853
Largest diff. peak/hole / e Å^{-3}	0.39/-0.23
Flack parameter	-0.008(2)

Extended Chemistry

Synthetic procedures for indol-3-acetates (3)

Ethyl 2-(4-[(3-chlorobenzyl)(methyl)carbamoyl)]-1*H*-indol-3-yl)acetate (**3b**). The title compound was produced by following the general procedure using 1-(3-chlorophenyl)-*N*-methylmethanamine (37.35mg) to yield **3b** as a yellow oil (65.7 mg, 0,17 mmol, 84%). R_f = 0.34 (cyclohexane/ethyl acetate 1:1). ^{1}H-NMR (400 MHz, DMSO-d_6): δ = 11.25 (s, 1H minor isomer), 11.23 (s, 1H major isomer), 7.31 – 7.49 (m, 5H major isomer, 5H minor isomer), 7.17 – 7.22 (m, 1H major isomer, 1H minor isomer), 7.11 – 7.15 (m, 1H major isomer), 7.01 – 7.05 (m, 1 minor isomer), 6.91 (d, 1H, *J* = 7.3 Hz, major isomer), 6.86 (d, 1H, J = 7.3 Hz, minor isomer), 4.67 (br. s, 2H major isomer), 4.28 (br. s, 2H minor isomer), 4.10 (quart, 2H, *J* = 7.1 Hz, minor isomer), 4.01 (quart, 2H, *J* = 7.1 Hz, major isomer), 3.68 (s, 2H, minor isomer), 3.64 (s, 2H, major isomer), 2.92 (s, 3H, minor isomer), 2.73 (s, 3H, major isomer), 1.15 – 1.23 (m, 3H major isomer, 3H minor isomer). ^{13}C-NMR (101 MHz, DMSO-d_6): δ = 171.7, 171.6, 170.8, 170.5, 140.2, 139.7, 136.9, 136.8, 133.4, 133.2, 130.6, 130.5, 127.9, 127.7, 127.5, 127.3, 127.2, 126.8, 126.6, 126.3, 125.9, 125.5, 123.1, 123.1, 120.4, 120.3, 116.9, 116.2, 112.6, 112.5, 106.9, 106.8, 60.1, 60.0, 53.7, 49.1, 36.9, 32.3, 30.9, 30.9, 14.2, 14.2. LRMS (ESI) *m/z* = 385 [M+H].

Ethyl 2-(4-[methyl((4-methyl-4*H*-thieno[3,2-*b*]pyrrol-5-yl)methyl)carbamoyl]-1*H*-indol-3-yl)acetate (**3c**). The title compound was produced by following the general procedure using *N*-methyl-1-(4-methyl-4*H*-thieno[3,2-*b*]pyrrol-5-yl)methanamine (43.26 mg) to yield **3c** as a yellow oil (37.7 mg, 0,092 mmol, 46%). R_f = 0.20 (cyclohexane/ethyl acetate 1:1). ^{1}H-NMR (400 MHz, DMSO-d_6): δ = 11.24 (s, 1H minor isomer), 11.22 (s, 1H major isomer), 7.44 – 7.42 (m, 1H major isomer, 1H minor isomer), 7.32 – 7.34 (m, 1H major isomer, 1H minor isomer), 7.21 (d, 1H, *J* = 5.1 Hz, major isomer), 7.02 – 7.17 (m, 2H major isomer, 4H minor isomer), 6.96 (d, 1H, *J* = 7.1 Hz, minor isomer), 6.88 (d, 1H, *J* = 6.8 Hz, major isomer), 6.47 (s, 1H major isomer), 6.34 (s, 1H minor isomer), 4.78 (br. s, 2H major isomer) 4.36 (br. s, 2H minor isomer), 4.10 (quart, 2H, *J* = 7.1 Hz, minor isomer), 4.00 (quart, 2H, *J* = 7.1 Hz, major isomer), 3.80 (s, 3H major isomer), 3.68 – 3.70 (m, 2H major isomer, 2H minor isomer), 3.44 (s, 3H, minor isomer), 2.99 (s, 3H minor isomer), 2.68 (s, 3H major isomer), 1.22 (t, 3H, *J* = 7.1 Hz, minor isomer), 1.17 (t, 3H, *J* = 7.1 Hz, major isomer).^{13}C-NMR (101 MHz, DMSO-d_6): δ = 171.8, 171.7, 170.5, 170.0, 141.4, 141.1, 136.9, 136.8, 133.7, 133.2, 127.9, 127.5, 126.2, 125.9, 123.1, 123.1, 122.8, 122.4, 120.8, 120.6, 120.4, 116.9, 116.3, 112.6, 112.4, 111.1, 110.9, 106.9, 106.8, 101.5, 99.3, 60.0, 47.4, 41.8, 36.0, 32.3, 32.0, 31.7, 30.9, 14.2. LRMS (ESI) *m/z* = 410 [M+H].

Ethyl 2-(4-[(4-bromobenzyl)(methyl)carbamoyl]-1*H*-indol-3-yl)acetate (**3d**). The title compound was produced by following the general procedure using 1-(4-bromophenyl)-*N*-methylmethanamine (48.02 mg) to yield **3d** as a yellow oil (68.7 mg, 0,16 mmol, 80%). R_f = 0.34 (cyclohexane/ethyl acetate 1:1). ^{1}H-NMR (400 MHz, DMSO-d_6): δ = 11.24 (s, 1H minor isomer), 11.22 (s, 1H major isomer), 7.57 – 7.59 (m, 2H major isomer), 7.52 – 7.54 (m, 2H minor isomer), 7.31 – 7.44 (m, 3H major isomer, 3H minor isomer), 7.10 – 7.17 (m, 2H major isomer, 1H minor isomer), 7.00 – 7.04 (m, 1H minor isomer), 6.90 (d, 1H, *J* = 7.3 Hz, major isomer), 6.86 (d, 1H, *J* = 7.3 Hz, minor isomer), 4.64 (br. s, 2H major isomer), 4.30 (br. s, 1H minor isomer), 4.07 – 4.12 (m, 3H minor isomer), 4.00 (quart, 2H, *J* = 7.1 Hz, major isomer), 3.68 (s, 2H minor isomer), 3.63 (s, 2H major isomer), 2.90 (s, 3H minor isomer), 2.70 (s, 3H major isomer), 1.21 (t, 3H, *J* = 7.1 Hz, minor isomer), 1.18 (t, 3H, *J* = 7.1 Hz, minor isomer). ^{13}C-NMR (101 MHz, DMSO-d_6): δ = 171.7, 171.6, 170.8, 170.4, 137.0, 136.9, 136.8, 136.5, 131.5, 131.4, 130.3, 129.1, 127.8, 127.5, 126.3, 125.9, 123.1, 120.4, 120.3, 120.3, 116.9, 116.2, 112.6, 112.4, 106.9, 106.8, 60.1, 60.0, 53.6, 49.0, 36.8, 32.2, 30.9, 30.8, 14.2, 14.2. LRMS (ESI) *m/z* = 429 [M+H].

Ethyl 2-(4-[methyl((3-methylthiophen-2-yl)methyl)carbamoyl]-1*H*-indol-3-yl)acetate (**3e**). The title compound was produced by following the general procedure using *N*-methyl-1-(3-methylthiophen-2-yl)methanamine (33.90 mg) to yield **3e** as a yellow oil (38.5 mg, 0,10 mmol, 52%). R_f = 0.31 (cyclohexane/ethyl acetate 1:1). ^{1}H-NMR (400 MHz, DMSO-d$_6$): δ = 11.25 (s, 1H minor isomer), 11.22 (s, 1H minor isomer), 7.30 – 7.45 (m, 3H major isomer, 3H minor isomer), 7.08 – 7.13 (m, 1H major isomer, 1H minor isomer), 6.95 (d, 1H, *J* = 7.0 Hz, minor isomer), 6.88 (d, 1H, *J* = 5.1 Hz, major isomer), 6.80 – 6.83 (m, 1H major isomer, 1H minor isomer), 4.77 (br. s, 2H major isomer), 4.32 (br. s, 2H minor isomer), 4.09 (quart, 2H, *J* = 7.1 Hz, minor isomer), 3.99 (quart, 2H, *J* = 7.1 Hz, major isomer), 3.66 (s, 2H major isomer, 2H minor isomer), 2.93 (s, 3H, minor isomer), 2.71 (s, 3H, major isomer), 2.63 (s, 3H major isomer), 1.92 (s, 3H minor isomer), 1.15 – 1.23 (m, 3H major isomer, 3H minor isomer). ^{13}C-NMR (101 MHz, DMSO-d$_6$): δ = 171.7, 171.7, 170.4, 170.0, 136.9, 136.8, 135.1, 134.5, 133.4, 133.0, 130.1, 129.9, 127.7, 127.4, 126.2, 125.9, 124.1, 123.9, 123.1, 123.1, 120.4, 116.8, 116.6, 112.6, 112.4, 107.0, 106.8, 60.0, 60.0, 47.7, 42.4, 36.4, 32.0, 30.9, 30.8, 14.2, 14.2, 13.4, 13.1. LRMS (ESI) *m/z* = 371 [M+H].

Ethyl 2-(4-[(benzo[*d*][1,3]dioxol-5-ylmethyl)(methyl)carbamoyl]-1*H*-indol-3-yl)acetate (**3f**). The title compound was produced by following the general procedure using 1-(benzo[*d*][1,3]dioxol-5-yl)-*N*-methylmethanamine (39.65 mg) to yield **3f** as a yellow oil (77.3 mg, 0,20 mmol, 98%). R_f = 0.26 (cyclohexane/ethyl acetate 1:1). ^{1}H-NMR (400 MHz, DMSO-d$_6$): δ = 11.24 (s, 1H minor isomer), 11.21 (s, 1H major isomer), 7.41 – 7.44 (m, 1H major isomer, 1H minor isomer), 7.30 – 7.34 (m, 1H major isomer, 1H minor isomer), 7.04 – 7.13 (m, 1H major isomer, 1H minor isomer), 6.86 – 6.98 (m, 3H major isomer, 3H minor isomer), 6.74 (d, 1H, *J* = 1.3 Hz, minor isomer), 6.65 (d, 1H, J = 8.0 Hz, minor isomer), 6.02 (s, 2H major isomer), 6.00 (s, 2H minor isomer), 4.57 (br. s, 2H major isomer), 4.26 (br. s, 1H minor isomer), 4.12 – 4.26 (m, 3H minor isomer), 4.01 (quart, 2H, *J* = 7.1 Hz, major isomer), 3.67 (s, 2H, minor isomer), 3.64 (s, 2H, major isomer), 2.88 (s, 3H, minor isomer), 2.67 (s, 3H, major isomer), 1.16 – 1.22 (m, 3H major isomer, 3H minor isomer). ^{13}C-NMR (101 MHz, DMSO-d$_6$): δ = 171.7, 171.6, 170.6, 170.3, 147.6, 147.5, 146.5, 146.5, 136.9, 136.8, 131.3, 130.7, 128.0, 127.7, 126.2, 125.9, 123.1, 123.1, 121.5, 120.4, 120.4, 120.2, 116.9, 116.4, 112.5, 112.3, 108.6, 108.3, 108.2, 107.4, 106.9, 106.8, 101.0, 100.9, 60.0, 60.0, 53.9, 49.3, 36.5, 31.9, 30.9, 30.8, 14.2, 14.1 LRMS (ESI) *m/z* = 395 [M+H].

Ethyl 2-(4-[(1*H*-indol-4-yl)methyl)(methyl)carbamoyl]-1*H*-indol-3-yl)acetate (**3g**). The title compound was produced by following the general procedure using 1-(1*H*-indol-4-yl)-*N*-methylmethanamine (38.45 mg) to yield **3g** as a yellow oil (23.4 mg, 0,060 mmol, 30%). R_f = 0.17 (cyclohexane/ethyl acetate 1:1). ^{1}H-NMR (400 MHz, DMSO-d$_6$): δ = 11.23 – 11.21 (m, 2H major isomer, 2H minor isomer), 7.32 – 7.43 (m, 4H major isomer, 4H minor isomer), 6.81 – 7.14 (m, 4H major isomer, 4H minor isomer), 6.62 (s, 1H major isomer), 6.26 (s, 1H minor isomer), 4.49 – 5.43 (br. m, 2H major isomer, 2H minor isomer), 4.13 (quart, 2H, *J* = 7.1 Hz, minor isomer), 4.06 (quart, 2H, *J* = 7.1 Hz, major isomer), 3.75 (s, 2H minor isomer), 3.71 (s, 2H, major isomer), 2.96 (s, 3H minor isomer), 2.61 (s, 3H major isomer), 1.20 – 1.25 (m, 3H major isomer, 3H minor isomer). ^{13}C-NMR (101 MHz, DMSO-d$_6$): δ = 171.8, 171.7, 170.8, 170.0, 136.9, 136.8, 136.0, 135.9, 128.2, 128.2, 127.9, 126.7, 126.2, 125.9, 125.7, 125.4, 125.3, 123.2, 123.1, 121.1, 120.9, 120.4, 120.3, 118.7, 116.6, 116.2, 116.1, 112.4, 112.2, 110.9, 110.6, 107.0, 106.9, 99.5, 98.5, 60.1, 60.0, 52.5, 47.8, 36.4, 32.5, 30.9, 14.2, 14.2. LRMS (ESI) *m/z* = 390 [M+H].

Ethyl 2-(4-[(3-methoxybenzyl)(methyl)carbamoyl]-1*H*-indol-3-yl)acetate (**3h**). The title compound was produced by following the general procedure using 1-(3-methoxyphenyl)-*N*-methylmethanamine (36.29 mg) to yield **3h** as a yellow oil (57.1 mg, 0,15 mmol, 75%). R_f = 0.26 (cyclohexane/ethyl acetate 1:1). ^{1}H-NMR (400 MHz, DMSO-d$_6$): δ = 11.24 (s, 1H minor isomer), 11.22 (s, 1H major isomer), 7.40 – 7.44 (m, 1H major isomer, 1H minor isomer), 7.25 – 7.34 (m, 2H major isomer, 2H minor isomer), 7.11 – 7.15 (m, 1H major isomer), 7.02 – 7.05 (m, 1H major isomer), 6.77 – 6.98 (m, 3H major isomer, 4H minor isomer), 6.70 (s, 1H minor isomer), 4.64 (br. s, 2H major isomer), 4.35 (br. s, 1H minor isomer), 4.07 – 4.35 (m, 3H minor isomer), 4.01 (quart, 2H, *J* = 7.1 Hz, major isomer), 3.78 (s, 3H major isomer),

3.72 (s, 3H minor isomer), 3.69 (s, 2H minor isomer), 3.66 (s, 2H major isomer), 2.91 (s, 3H, minor isomer), 2.70 (s, 3H, major isomer), 1.16– 1.23 (m, 3H major isomer, 3H minor isomer). ^{13}C-NMR (101 MHz, DMSO-d$_6$): $\delta = 171.7$, 171.7, 170.8, 170.3, 159.5, 159.5, 139.1, 138.7, 136.9, 136.8, 129.8, 129.7, 127.9, 127.7, 126.2, 125.9, 123.1, 123.1, 120.5, 120.4, 120.0, 118.8, 116.8, 116.3, 113.5, 112.8, 112.7, 112.5, 112.4, 112.4, 107.0, 106.8, 60.1, 60.0, 54.1, 49.5, 36.7, 32.2, 30.9, 30.8, 14.2, 14.2. LRMS (ESI) *m/z* = 381 [M+H].

Ethyl 2-(4-[methyl((1-methyl-1*H*-benzo[*d*]imidazol-2-yl)methyl)carbamoyl]-1*H*-indol-3-yl)acetate (**3i**). The title compound was produced by following the general procedure using *N*-methyl-1-(1-methyl-1*H*-benzo[*d*]imidazol-2-yl)methanamine (42.06 mg) to yield **3i** as a yellow oil (29.1 mg, 0,072 mmol, 36%). $R_f = 0.21$ (cyclohexane/ethyl acetate 1:4). ^{1}H-NMR (400 MHz, DMSO-d$_6$): $\delta = 11.24$ (s, 1H major isomer), 11.20 (s, 1H minor isomer), 7.58 – 7.69 (m, 2H major isomer, 1H minor isomer), 7.38 – 7.50 (m, 1H major isomer, 2H minor isomer), 7.19 – 7.32 (m, 3H major isomer, 3H minor isomer), 7.11 – 7.15 (m, 1H major isomer), 6.88 – 6.97 (m, 1H major isomer, 2H minor isomer), 4.99 (br. s, 2H major isomer), 4.59 (br. s, 2H minor isomer), 4.11 (quart, 2H, *J* = 7.1 Hz, minor isomer), 3.91 – 3.97 (m, 5H major isomer), 3.73 – 3.79 (m, 2H, minor isomer), 3.69 (s, 2H major isomer), 3.43 (s, 3H minor isomer), 3.11 (s, 3H minor isomer), 2.86 (s, 3H major isomer), 1.23 (t, 3H, *J* = 7.1 Hz, minor isomer), 1.12 (t, 3H, *J* = 7.1 Hz, major isomer). ^{13}C-NMR (101 MHz, DMSO-d$_6$): $\delta = 172.0$, 171.7, 170.6, 170.5, 150.9, 150.5, 141.5, 136.9, 135.9, 135.8, 127.4, 127.4, 125.9, 125.9, 123.3, 123.1, 122.3, 122.0, 121.7, 121.6, 120.4, 120.2, 118.7, 118.6, 117.1, 116.2, 112.6, 110.3, 110.0, 107.1, 106.9, 60.0, 59.9, 47.6, 42.6, 37.2, 33.4, 31.0, 29.9, 29.3, 14.2, 14.1 LRMS (ESI) *m/z* = 405 [M+H].

Ethyl 2-(4-[(4-*tert*-butylbenzyl)(methyl)carbamoyl]-1*H*-indol-3-yl)acetate (**3j**). The title compound was produced by following the general procedure using 1-(4-*tert*-butylphenyl)-*N*-methylmethanamine (42.55 mg) to yield **3j** as a yellow oil (27.6 mg, 0,068 mmol, 34%). $R_f = 0.38$ (cyclohexane/ethyl acetate 1:1). ^{1}H-NMR (400 MHz, DMSO-d$_6$): $\delta = 11.23$ (s, 1H minor isomer), 11.21 (s, 1H major isomer), 7.31 – 7.44 (m, 5H minor isomer, 5H major isomer), 7.14 – 7.10 (m, 2H major isomer, 1H minor isomer), 7.06 – 7.02 (m, 1H minor isomer), 6.89 – 6.92 (m, 1H major isomer, 1H minor isomer), 4.62 (br. s, 2H major isomer), 4.35 (br. s, 1H minor isomer), 4.06 – 4.12 (m, 3H minor isomer), 4.02 (quart, *J* = 7.1 Hz, 2H major isomer), 3.68 (s, 2H minor isomer), 3.66 (s, 2H major isomer), 2.88 (s, 3H minor isomer), 2.68 (s, 3H major isomer), 1.29 (s, 9H major isomer), 1.26 (9H minor isomer), 1.16 – 1.23 (m, 3H major isomer, 3H minor isomer). ^{13}C-NMR (101 MHz, DMSO-d$_6$): $\delta = 171.7$, 171.7, 170.7, 170.3, 149.6, 149.5, 136.9, 136.8, 134.5, 133.9, 128.0, 127.8, 127.7, 126.6, 126.2, 125.8, 125.4, 125.3, 123.1, 123.1, 120.4, 120.4, 116.9, 116.3, 112.4, 112.3, 106.9, 106.8, 60.0, 60.0, 53.8, 49.1, 36.7, 34.2, 32.1, 31.2, 31.1, 30.9, 30.8, 14.2, 14.2. LRMS (ESI) *m/z* = 407 [M+H].

Ethyl 2-(4-[(3,4-dimethylbenzyl)(methyl)carbamoyl]-1*H*-indol-3-yl)acetate (**3k**). The title compound was produced by following the general procedure using 1-(3,4-dimethylphenyl)-*N*-methylmethanamine (35.82 mg) to yield **3k** as a yellow oil (42.4 mg, 0,11 mmol, 56%). $R_f = 0.35$ (cyclohexane/ethyl acetate 1:1). ^{1}H-NMR (400 MHz, DMSO-d$_6$): $\delta = 11.23$ (s, 1H minor isomer), 11.21 (s, 1H major isomer), 7.40 – 7.44 (m, 1H minor isomer, 1H major isomer), 7.34 (d, 1H, *J* = 2.3 Hz, minor isomer), 7.30 (d, 1H, *J* = 2.2 Hz, major isomer), 7.02 – 7.18 (m, 3H major isomer, 3H minor isomer), 6.87 – 6.92 (m, 2H major isomer, 2H minor isomer), 4.58 (br. s, 2H major isomer), 4.29 (br. s, 1H minor isomer), 4.06 – 4.12 (m, 3H minor isomer), 4.01 (quart, 2H, *J* = 7.1 Hz, major isomer), 3.67 (s, 2H minor isomer), 3.65 (s, 2H major isomer), 2.88 (s, 3H minor isomer), 2.65 (s, 3H major isomer), 2.18 – 2.22 (m, 6H major isomer, 6H minor isomer), 1.21 (t, 3H, *J* = 7.1 Hz, minor isomer), 1.18 (t, 3H, *J* = 7.1 Hz, minor isomer). ^{13}C-NMR (101 MHz, DMSO-d$_6$): $\delta = 171.7$, 171.6, 170.6, 170.2, 136.9, 136.8, 136.5, 136.3, 135.2, 135.1, 134.8, 134.2, 129.7, 129.6, 129.3, 128.1, 128.0, 127.8, 126.1, 125.8, 125.5, 124.3, 123.1, 123.0, 120.5, 120.4, 116.8, 116.3, 112.4, 112.3, 106.9, 106.8, 60.0, 60.0, 53.9, 49.2, 36.5, 32.0, 30.9, 30.8, 19.4, 19.4, 19.1, 19.0, 14.2, 14.2. LRMS (ESI) *m/z* = 379 [M+H].

Ethyl 2-(4-[(2-chloro-4,5-dimethoxybenzyl)(methyl)carbamoyl]-1*H*-indol-3-yl)acetate (**3l**). The title compound was produced by following the general procedure using 1-(2-chloro-4,5-dimethoxyphenyl)-*N*-methylmethanamine (51.76 mg) to yield **3l** as a yellow oil (73.0 mg, 0,16 mmol, 82%). R_f = 0.19 (cyclohexane/ethyl acetate 1:1). ^{1}H-NMR (400 MHz, DMSO-d$_6$): δ = 11.25 (s, 1H minor isomer), 11.22 (s, 1H major isomer), 7.40 – 7.45 (m, 1H major isomer, 1H minor isomer), 7.31 – 7.35 (m, 1H major isomer, 1H minor isomer), 7.11 – 7.15 (m, 1H major isomer), 6.99 – 7.07 (m, 2H major isomer, 2H minor isomer), 6.91 (d, 1H, *J* = 7.2 Hz, major isomer), 6.86 (d, 1H, J = 7.0 Hz, minor isomer), 6.81 (s, 1H minor isomer), 4.70 (br. s, 2H major isomer), 4.23 (br. s, 2H minor isomer), 4.09 (quart, 2H, *J* = 7.1 Hz, minor isomer), 4.00 (quart, 2H, *J* = 7.1 Hz, major isomer), 3.76 – 3.80 (m, 6H major isomer, 6H minor isomer), 3.70 (s, 2H, minor isomer), 3.68 (s, 2H, major isomer), 2.95 (s, 3H, minor isomer), 2.70 (s, 3H, major isomer), 1.17 – 1.23 (m, 3H major isomer, 3H minor isomer). ^{13}C-NMR (101 MHz, DMSO-d$_6$): δ = 171.8, 171.7, 171.0, 170.5, 148.8, 148.6, 148.1, 148.0, 136.9, 136.8, 127.8, 127.5, 126.3, 126.1, 126.0, 125.7, 123.8, 123.1, 123.1, 122.7, 120.5, 120.4, 116.8, 116.2, 113.0, 112.8, 112.6, 112.6, 112.5, 110.9, 106.9, 106.8, 60.1, 60.0, 55.9, 55.8, 55.7, 51.8, 46.9, 36.8, 32.5, 31.0, 30.9, 14.2, 14.1. LRMS (ESI) *m/z* = [M+H].

N-(4-*tert*-Butylbenzyl)-3-(2-isopropoxy-2-oxoethyl)-*N*-methyl-1*H*-indole-4-carboxamide (**3m**).

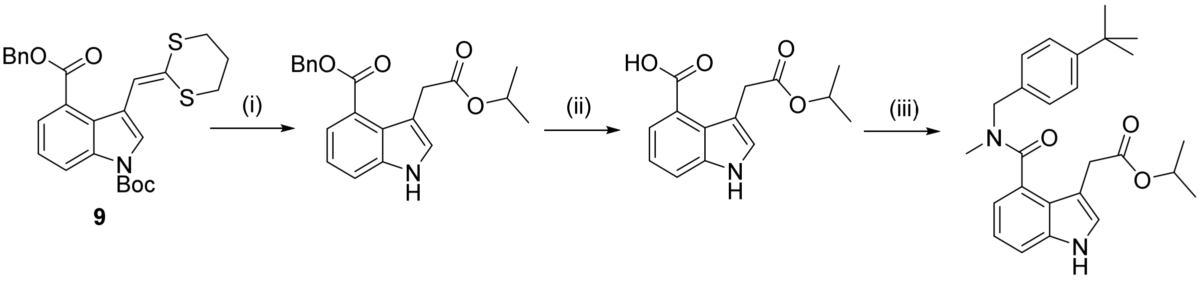

Step (i): To a suspension of **9** (488 mg, 1.73 mmol) in dry i-PrOH (45 mL) Hg_2Cl_2 (518 mg, 1.91 mmol) was added and the resulting mixture sonicated at 60 °C for 12 h. After cooling to room temperature, the suspension was filtered through Celite® and the solid was washed with isopropanol (100mL). The filtrate and the combined wash solutions were transferred to a round bottom flask and the solvent was evaporated. After flash chromatography (cyclohexane/ethyl acetate 2:1) the desired product was obtained as a brown solid (175 mg, 0.5 mmol, 29 %). Step (ii): To a solution of benzyl 3-(2-isopropoxy-2-oxoethyl)-1*H*-indole-4-carboxylate (445 mg, 1.266 mmol) in dry i-PrOH (40 mL) Pd(0) (10%/C, 25.68 mg, 0.025 mmol) was added and a hydrogen-filled balloon fitted on the apparatus. The mixture was heated to 60 °C for 12 h. After cooling to room temperature, the suspension was filtered through Celite® and the solid was washed with i-PrOH (20 mL). The volatile components of the filtrate and the combined washing solutions were evaporated in vacuo. The desired product was obtained as a brown solid (309 mg, 1.183 mmol, 93 %). Step (iii): The title compound was produced by following the general procedure by using 3-(2-isopropoxy-2-oxoethyl)-1*H*-indole-4-carboxylic acid (100 mg, 0.383 mmol) and *N*-methyl-1-[4-(*tert*-butyl)phenyl]methanamine (81 mg, 0.460 mmol) with HATU (437 mg, 1.149 mmol) and DIPEA (200 µL, 198 mg, 1.532 mmol) in DMF (5 mL) to yield **3m** as a yellow solid (122 mg, 0.290 mmol, 82%). R_f = 0.5 (cyclohexane/ethylacetate 1:1). ^{1}H-NMR (400 MHz, DMSO-d$_6$): δ = 11.20 – 11.22 (s, 1H minor isomer, 1h major isomer), 6.88-7.44 (m, 8H major isomer, 8H minor isomer), 4.85 – 4.95 (m, 1H minor isomer, 1H major isomer), 4.12 – 4.66 (br. m. 1H minor isomer, 1H major isomer), 3.65 (s, 1H minor isomer), 3.61 (s,1H major isomer), 2.89 (s, 3H minor isomer), 2.69 (s, 3H major isomer), 1.29 (s, 9H major isomer), 1.26 (s, 9H minor isomer), 1.19 – 1.22 (m, 6H minor isomer, 6H major isomer). ^{13}C-NMR (101 MHz, DMSO-d$_6$): δ 171.0, 171.0, 170.6, 170.2, 149.5, 149.4, 136.7, 136.6, 134.3, 133.7, 127.9, 127.7, 127.5, 126.5, 125.9, 125.6, 125.3, 125.2, 123.0, 122.9, 120.3, 120.3, 116.8, 116.3, 112.3, 112.2, 106.8, 106.7, 67.2, 67.1, 53.7, 49.1, 36.6, 34.1, 31.9, 31.1, 31.0, 30.9, 21.6, 21.6. HRMS (ESI) m/z calc. for $C_{26}H_{32}N_2O_3$ $[M+H]^+$: 421.2491, found: 421.2478.

Ethyl 2-(4-[(3-*tert*-butylbenzyl)(methyl)carbamoyl]-1*H*-indol-3-yl)acetate (**3o**). The title compound was produced by following the general procedure using 1-(3-*tert*-butylphenyl)-*N*-methylmethanamine (42.55 mg) to yield **3o** as a yellow oil (27.6 mg, 0,068 mmol, 34%). R_f = 0.43 (cyclohexane/ethyl acetate 1:1). ^{1}H-NMR (400 MHz, DMSO-d$_6$): δ = 11.24 (s, 1H minor isomer), 11.23 (s, 1H major isomer), 7.41 – 7.44 (m, 2H major isomer, 1H minor isomer), 7.02 – 7.35 (m, 5H major isomer, 6H minor isomer), 6.87 – 6.88 (m, 1H major isomer, 1H minor isomer), 4.67 (br. s, 2H major isomer), 4.35 (br. s, 1H minor isomer), 4.07 – 4.13 (m, 3H minor isomer), 4.02 (quart, 2H, *J* = 7.1 Hz, major isomer), 3.68 (s, 2H, minor isomer), 3.65 (s, 2H, major isomer), 2.93 (s, 3H, minor isomer), 2.69 (s, 3H, major isomer), 1.16 – 1.31 (m, 12H major isomer, 12H minor isomer). ^{13}C-NMR (101 MHz, DMSO-d$_6$): δ = 171.7, 171.6, 170.7, 170.3, 151.0, 150.9, 137.1, 136.9, 136.7, 136.5, 128.4, 128.3, 128.0, 127.8, 126.2, 125.8, 125.0, 124.6, 124.1, 124.1, 124.0, 123.7, 123.0, 120.5, 120.3, 116.7, 116.3, 112.4, 112.3, 107.0, 106.8, 60.0, 60.0, 54.3, 49.7, 36.7, 34.4, 34.3, 32.2, 31.2, 31.1, 31.0, 30.8, 14.2, 14.2. LRMS (ESI) *m/z* = 407 [M+H].

Isopropyl 2-(4-(methyl(4'-(trifluoromethyl)-[1,1'-biphenyl]-4-yl)carbamoyl)-1*H*-indol-3-yl)acetate (**3p**).

The title compound was produced by following the general procedure by using 3-(2-Isopropoxy-2-oxoethyl)-1*H*-indole-4-carboxylic acid (100 mg, 0.383 mmol) (obtained by the procedure described above for **3m**) and *N*-methyl-1-[4'-(trifluoromethyl)-(1,1'-biphenyl)-4-yl]methanamine hydrochloride (139 mg, 0.460 mmol) with HATU (437 mg, 1.15 mmol) and DIPEA (267 µL, 198 mg, 1.532 mmol) in DMF (5 mL) to yield **3p** as a yellow solid (160 mg, 0.32 mmol, 82%). R_f = 0.58 (DCM/MeOH 98:2). ^{1}H-NMR (400 MHz, DMSO-d$_6$): δ 11.24 (s, 1H minor isomer), 11.22 (s, 1H major isomer), 7.88 – 7.93(m, 2H minor isomer, 2H major isomer), 7.76 – 7.84 (m, 3H minor isomer, 3H major isomer), 7.55 – 7.73 (m, 2H minor isomer, 2H major isomer), 7.31 – 7.46 (m , 3H minor isomer, 3H major isomer), 7.14 (m, 1H major isomer), 7.05 (m, 1H minor isomer), 6.94 (m, 1H minor isomer, 1H major isomer), 4.94 (m, 1H minor isomer), 4.87 (m, 1H major isomer), 4.75 (br. s, 2H major isomer), 4.40 (br. S, 2H minor isomer), 3.67 (br. s, 2H minor isomer), 3.62 (s, 2H major isomer), 2.96 (s, 3H minor isomer), 2.75 (s, 3H major isomer), 1.23 (d, 6H minor isomer), 1.19 (d, 6H major isomer). ^{13}C-NMR (101 MHz, DMSO-d$_6$): δ 171.2, 171.1, 170.8, 170.4, 143.9, 143.7, 137.9, 137.5, 137.4, 136.8, 136.7, 128.7, 127.8, 127.6, 127.4, 127.3, 127.3, 126.1, 125.8, 125.8, 123.1, 123.0, 120.4, 120.3, 116.9, 116.3, 112.5, 112.4, 106.9, 106.8, 67.3, 62.2, 53.8, 49.3, 36.9, 32.2, 31.1, 31.1, 21.7, 21.6. HRMS (ESI) m/z calc. for C29H28F3N2O3 [M+H]+: 509.2052, found: 509.2047.

^{1}H and ^{13}C NMR spectra of newly synthesized compounds

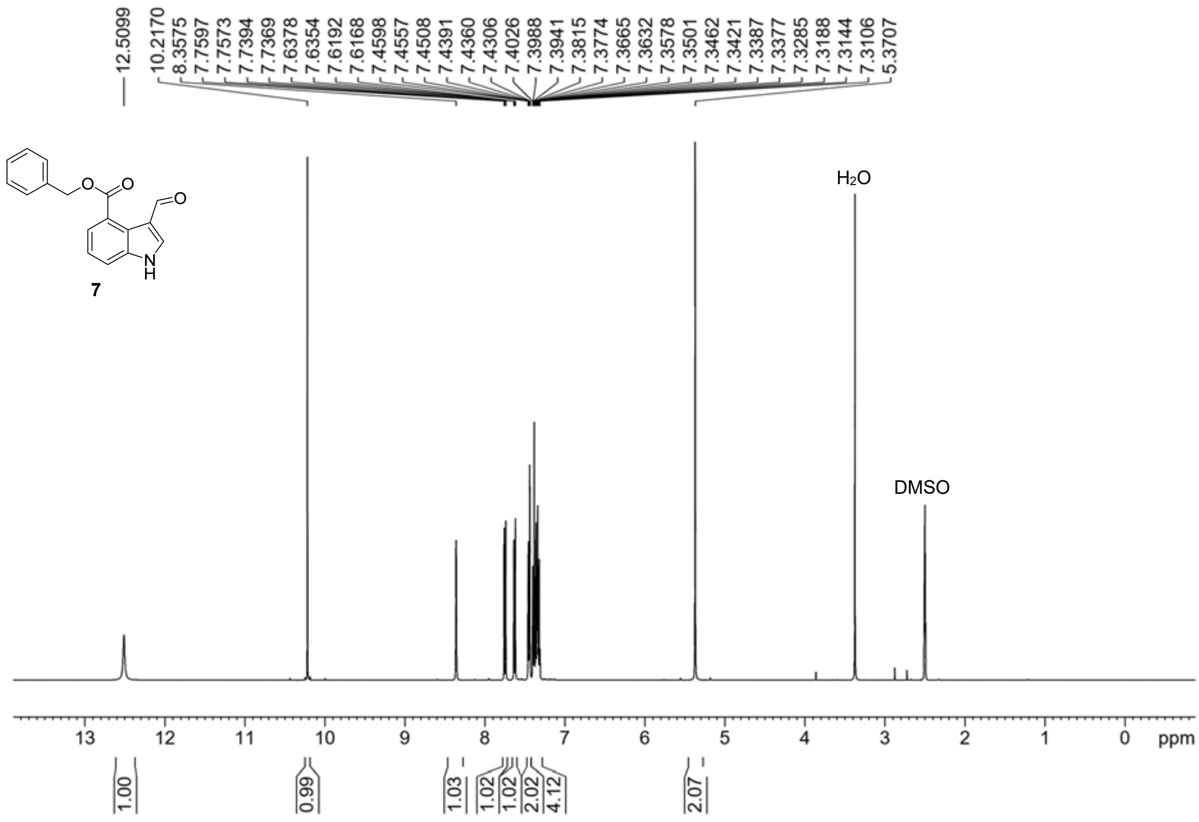

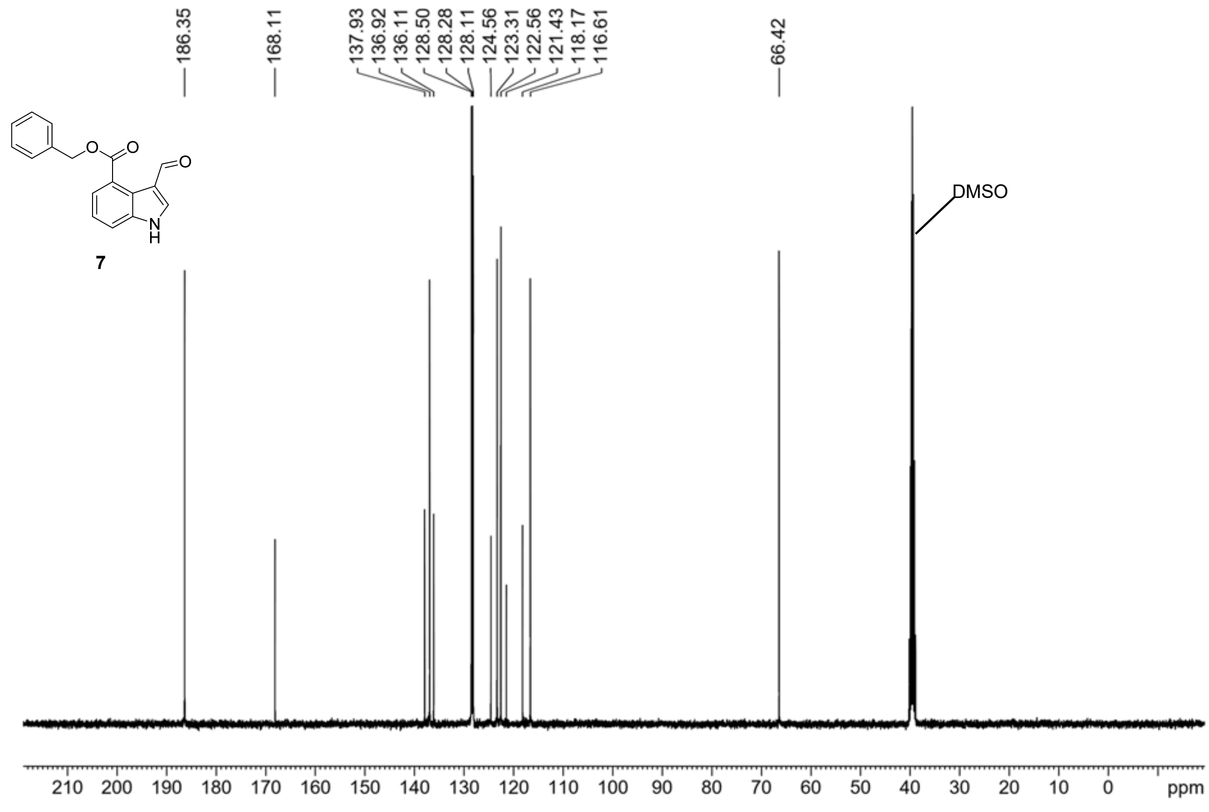

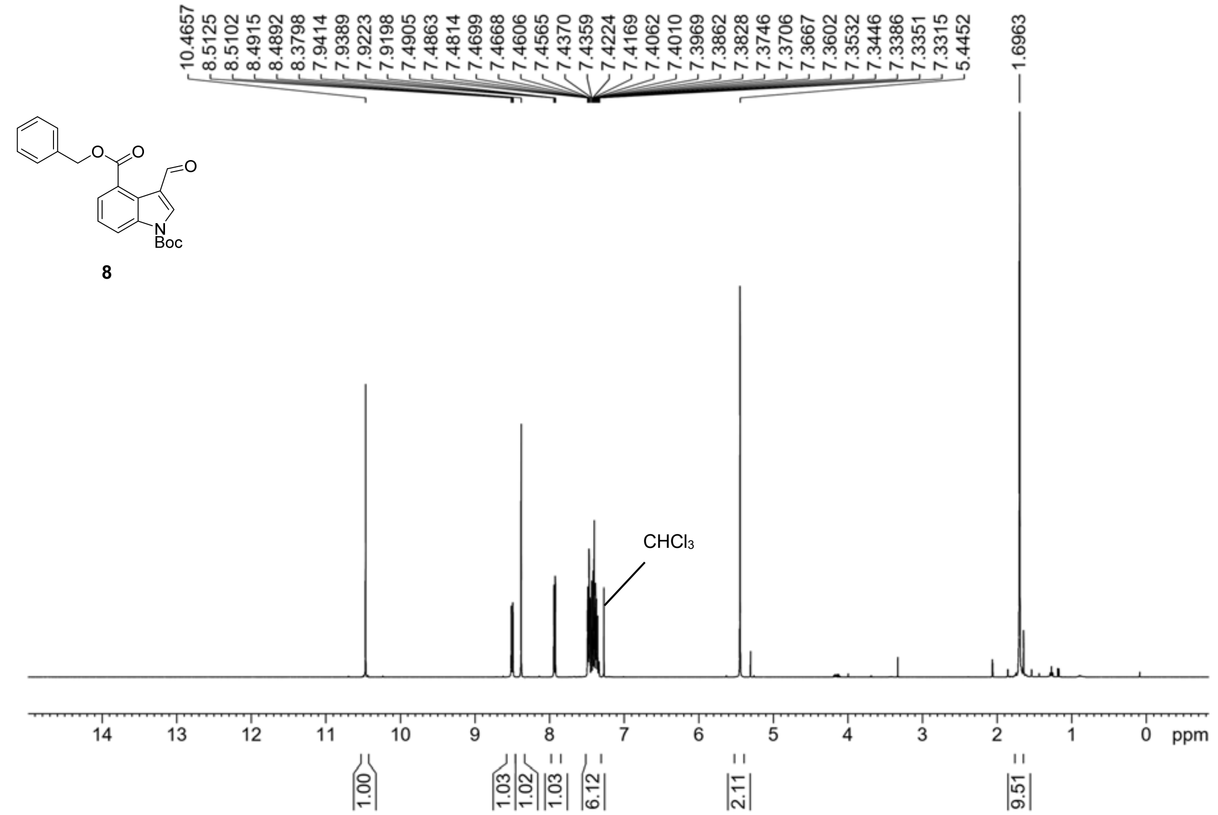
10.4657
8.5125
8.5102
8.4915
8.4892
8.3798
7.9414
7.9389
7.9223
7.9198
7.4905
7.4863
7.4814
7.4699
7.4668
7.4606
7.4565
7.4370
7.4359
7.4224
7.4169
7.4062
7.4010
7.3969
7.3862
7.3828
7.3746
7.3706
7.3667
7.3602
7.3532
7.3446
7.3386
7.3351
7.3315
5.4452
1.6963
Boc
CHCl3
14 13 12 11 10 9 8 7 6 5 4 3 2 1 0 ppm
1.00
1.03
1.02
1.03
6.12
2.11
9.51

8

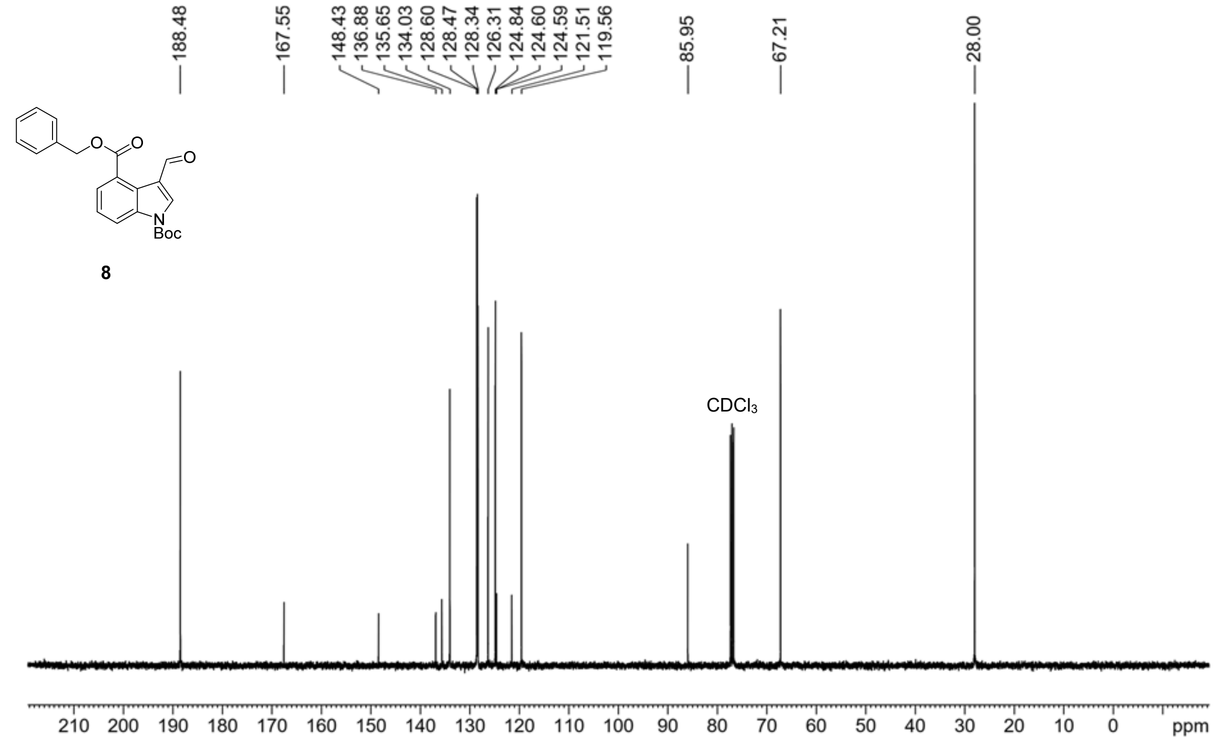
188.48
167.55
148.43
136.88
135.65
134.03
128.60
128.47
128.34
126.31
124.84
124.60
124.59
121.51
119.56
85.95
67.21
28.00
Boc
CDCl3
210 200 190 180 170 160 150 140 130 120 110 100 90 80 70 60 50 40 30 20 10 0 ppm

8

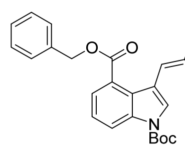
S
S
O
O
N
Boc

9

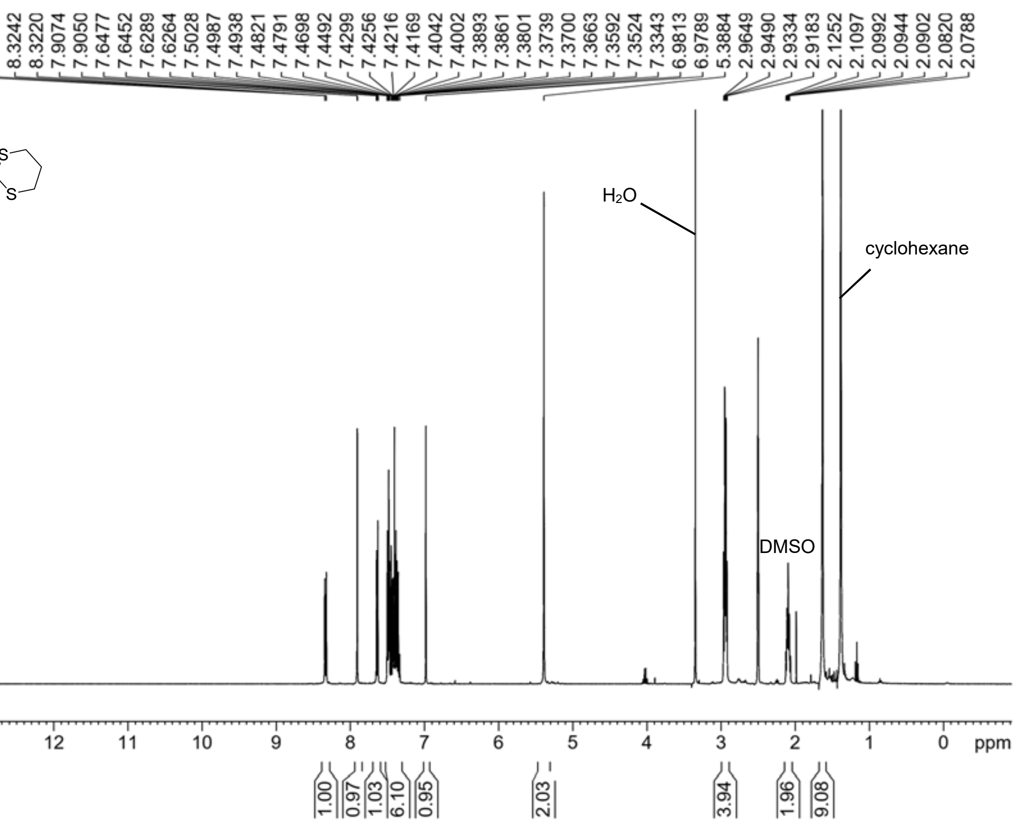
8.3451
8.3429
8.3242
8.3220
7.9074
7.9050
7.6477
7.6452
7.6289
7.6264
7.5028
7.4987
7.4938
7.4821
7.4791
7.4698
7.4492
7.4299
7.4256
7.4216
7.4169
7.4042
7.4002
7.3893
7.3861
7.3801
7.3739
7.3700
7.3663
7.3592
7.3524
7.3343
6.9813
6.9789
5.3884
2.9649
2.9490
2.9334
2.9183
2.1252
2.1097
2.0992
2.0944
2.0902
2.0820
2.0788
H2O
cyclohexane
DMSO
14 13 12 11 10 9 8 7 6 5 4 3 2 1 0 ppm
1.00
0.97
1.03
6.10
0.95
2.03
3.94
1.96
9.08

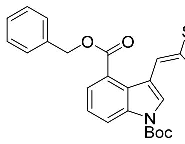
S
S
O
O
N
Boc

9

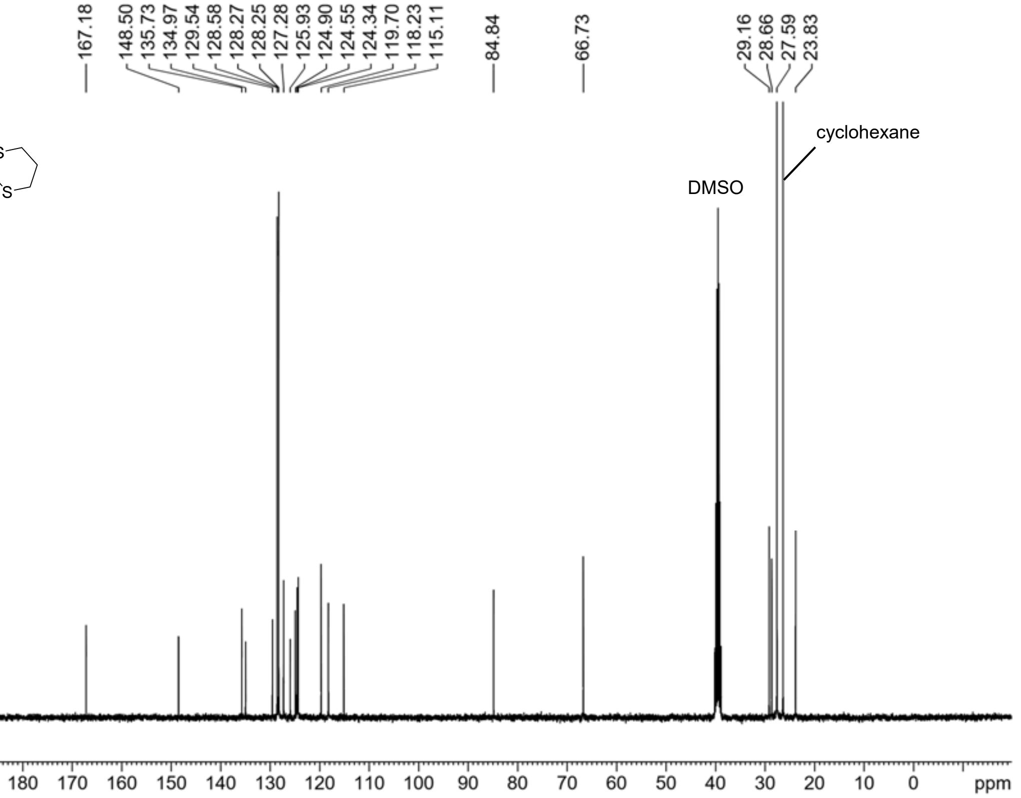
167.18
148.50
135.73
134.97
129.54
128.58
128.27
128.25
127.28
125.93
124.90
124.55
124.34
119.70
118.23
115.11
84.84
66.73
29.16
28.66
27.59
23.83
cyclohexane
DMSO
210 200 190 180 170 160 150 140 130 120 110 100 90 80 70 60 50 40 30 20 10 0 ppm

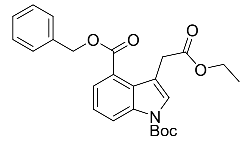

10

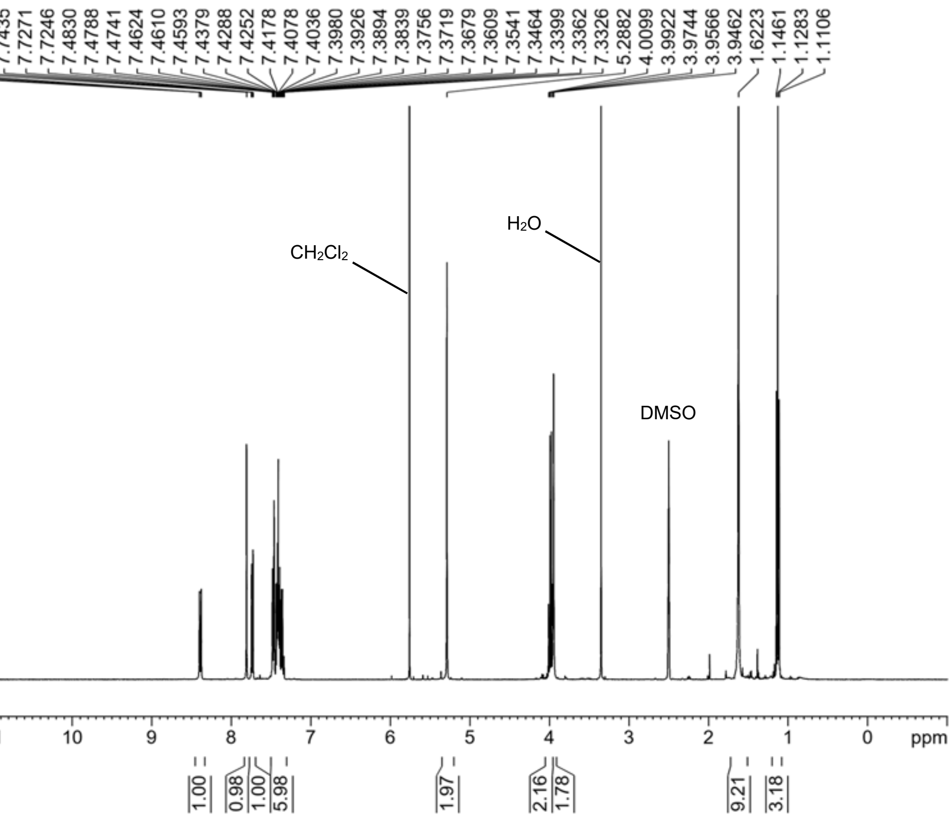

8.3999
8.3980
8.3792
8.3771
7.8073
7.7460
7.7435
7.7271
7.7246
7.4830
7.4788
7.4741
7.4624
7.4610
7.4593
7.4379
7.4288
7.4252
7.4178
7.4078
7.4036
7.3980
7.3926
7.3894
7.3839
7.3756
7.3719
7.3679
7.3609
7.3541
7.3464
7.3399
7.3362
7.3326
5.2882
4.0099
3.9922
3.9744
3.9566
3.9462
1.6223
1.1461
1.1283
1.1106
CH2Cl2
H2O
DMSO
13 12 11 10 9 8 7 6 5 4 3 2 1 0 ppm
1.00
0.98
1.00
5.98
1.97
2.16
1.78
9.21
3.18

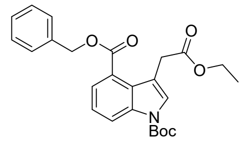

10

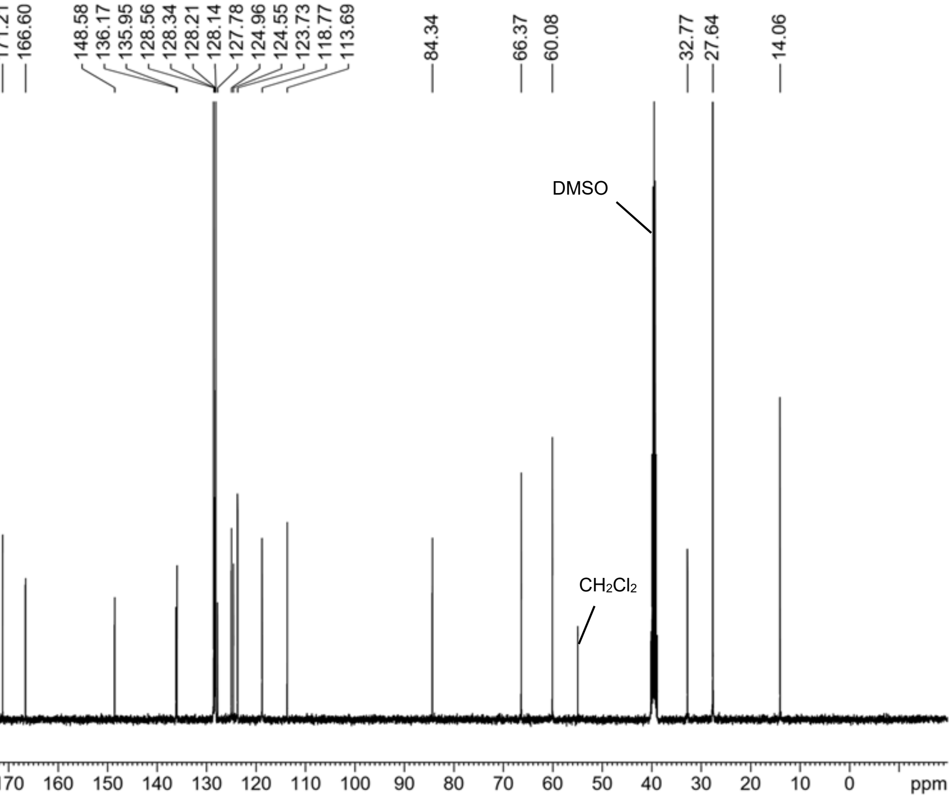

171.21
166.60
148.58
136.17
135.95
128.56
128.34
128.21
128.14
127.78
124.96
124.55
123.73
118.77
113.69
84.34
66.37
60.08
32.77
27.64
14.06
DMSO
CH2Cl2
210 200 190 180 170 160 150 140 130 120 110 100 90 80 70 60 50 40 30 20 10 0 ppm

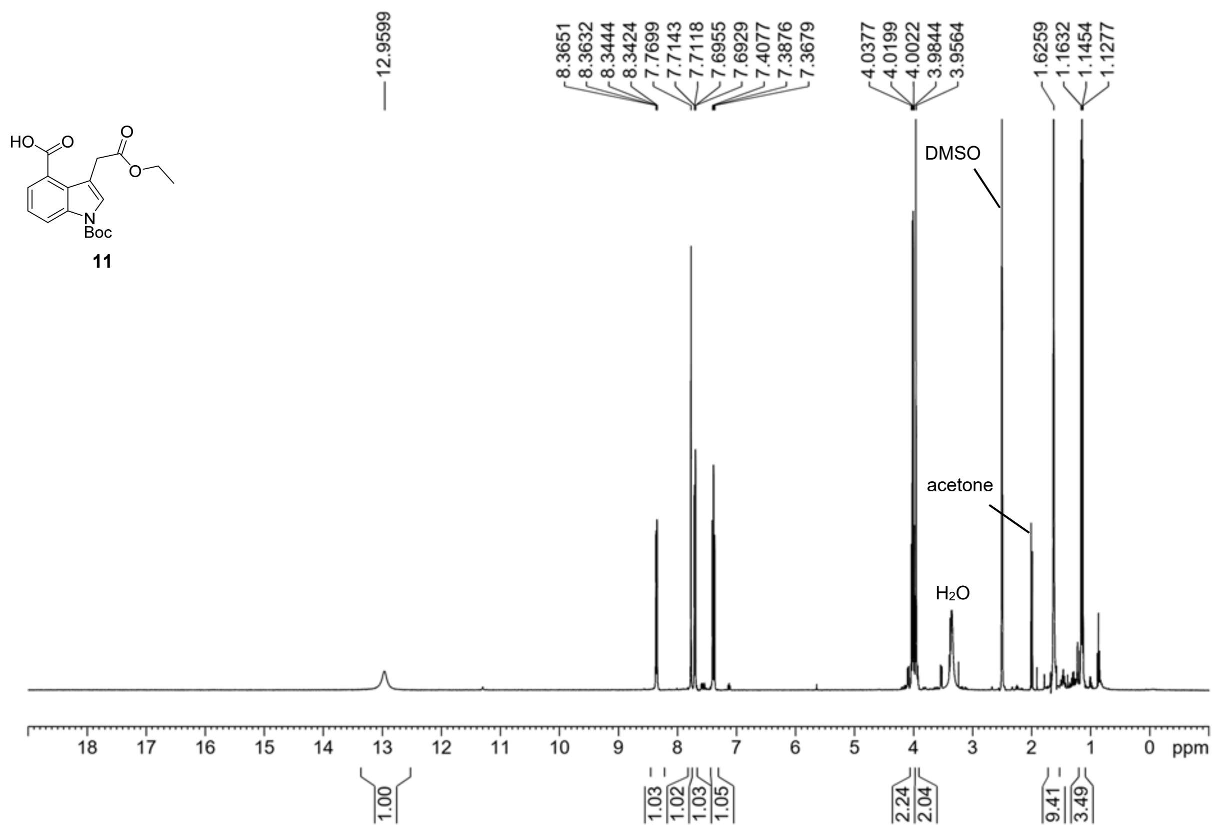
HO
O
O
O
N
Boc
11
12.9599
8.3651
8.3632
8.3444
8.3424
7.7699
7.7143
7.7118
7.6955
7.6929
7.4077
7.3876
7.3679
4.0377
4.0199
4.0022
3.9844
3.9564
1.6259
1.1632
1.1454
1.1277
DMSO
acetone
H_2O
18 17 16 15 14 13 12 11 10 9 8 7 6 5 4 3 2 1 0 ppm
1.00
1.03
1.02
1.03
1.05
2.24
2.04
9.41
3.49

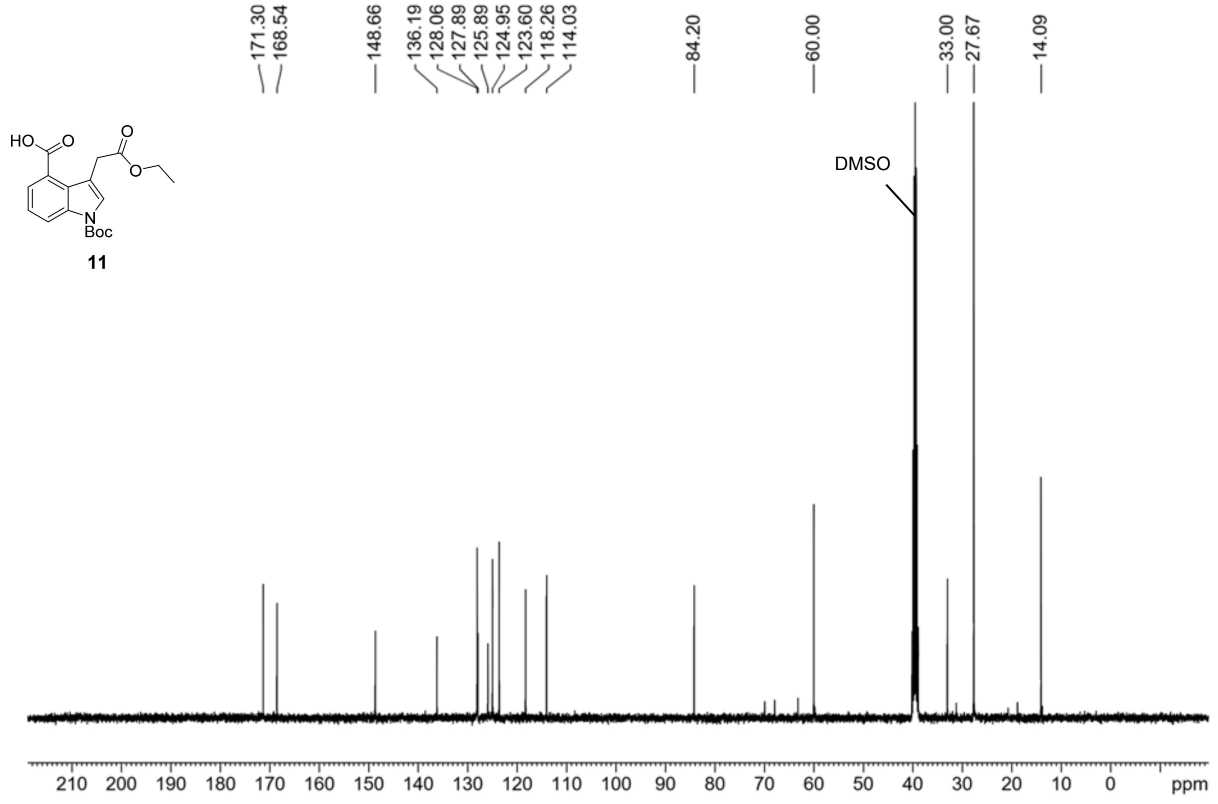
HO
O
O
O
N
Boc
11
171.30
168.54
148.66
136.19
128.06
127.89
125.89
124.95
123.60
118.26
114.03
84.20
60.00
33.00
27.67
14.09
DMSO
210 200 190 180 170 160 150 140 130 120 110 100 90 80 70 60 50 40 30 20 10 0 ppm

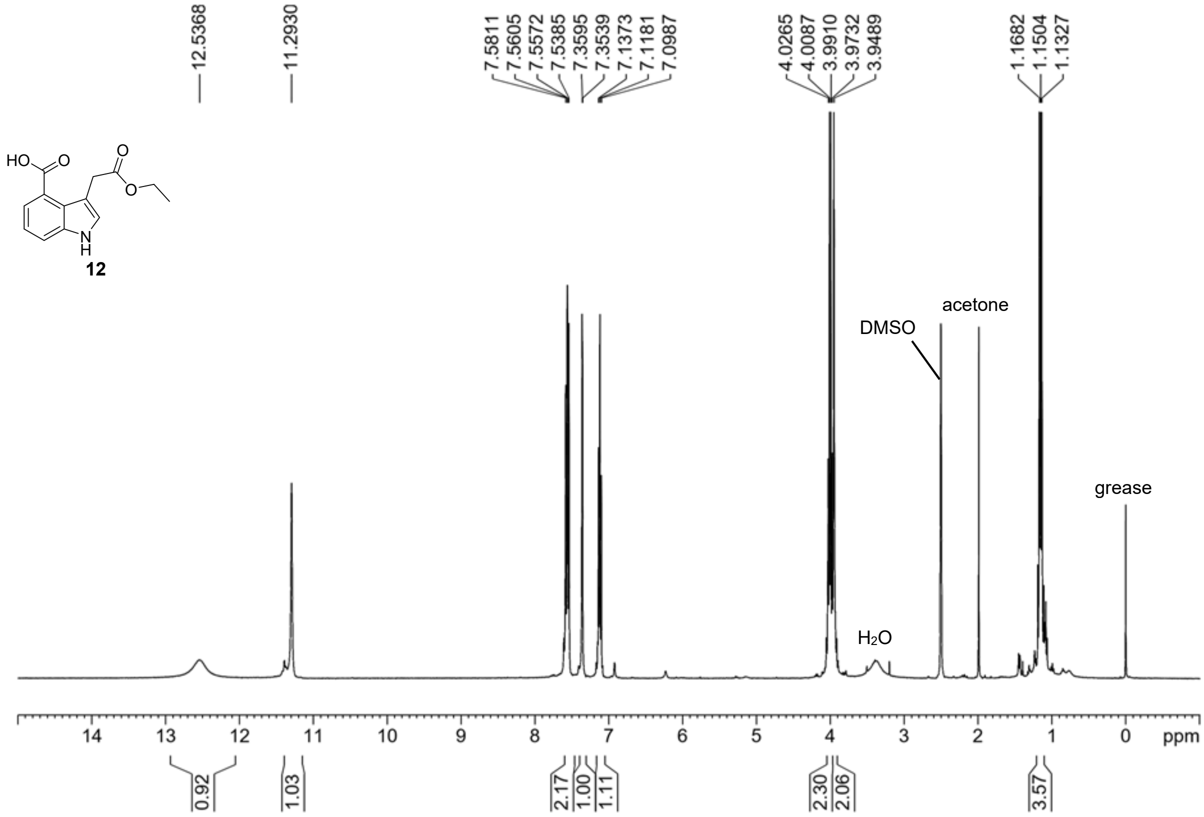

12.5368
11.2930
7.5811
7.5605
7.5572
7.5385
7.3595
7.3539
7.1373
7.1181
7.0987
4.0265
4.0087
3.9910
3.9732
3.9489
1.1682
1.1504
1.1327
HO
O
O
O
N
H
12
DMSO
acetone
grease
H2O
14 13 12 11 10 9 8 7 6 5 4 3 2 1 0 ppm
0.92
1.03
2.17
1.00
1.11
2.30
2.06
3.57

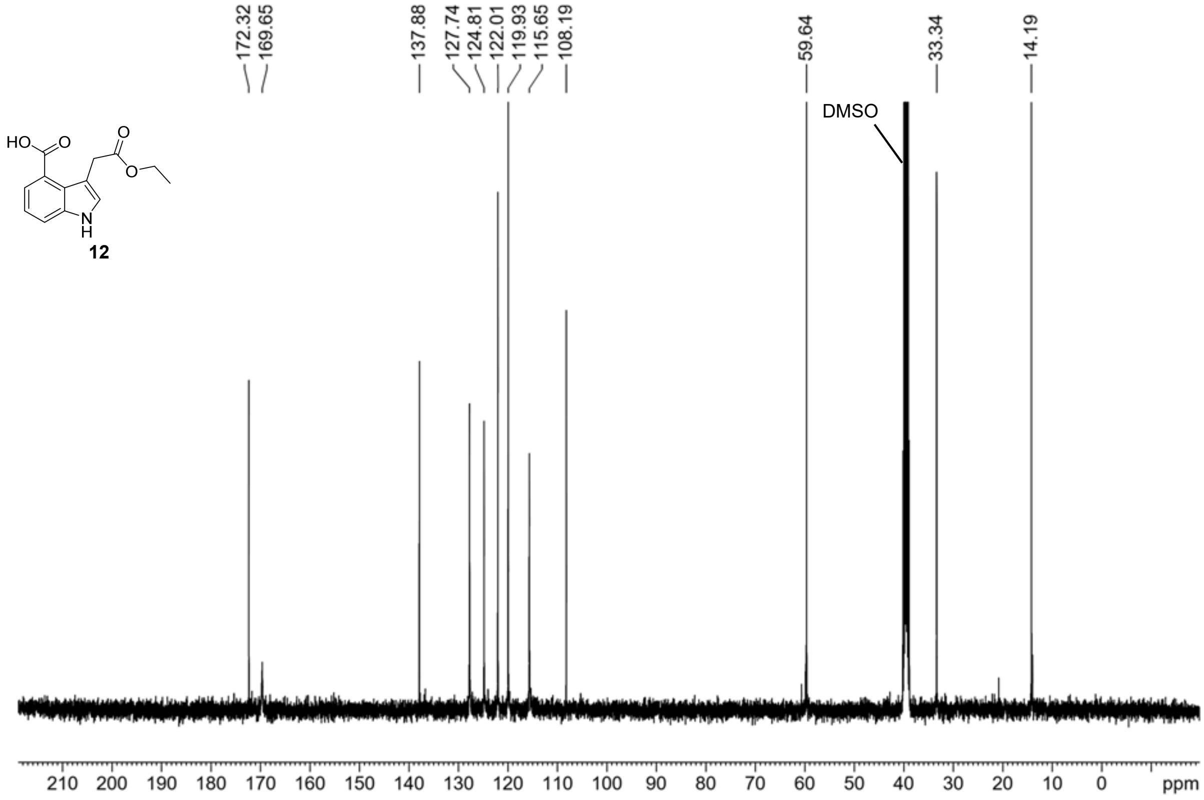

172.32
169.65
137.88
127.74
124.81
122.01
119.93
115.65
108.19
59.64
33.34
14.19
HO
O
O
O
N
H
12
DMSO
210 200 190 180 170 160 150 140 130 120 110 100 90 80 70 60 50 40 30 20 10 0 ppm

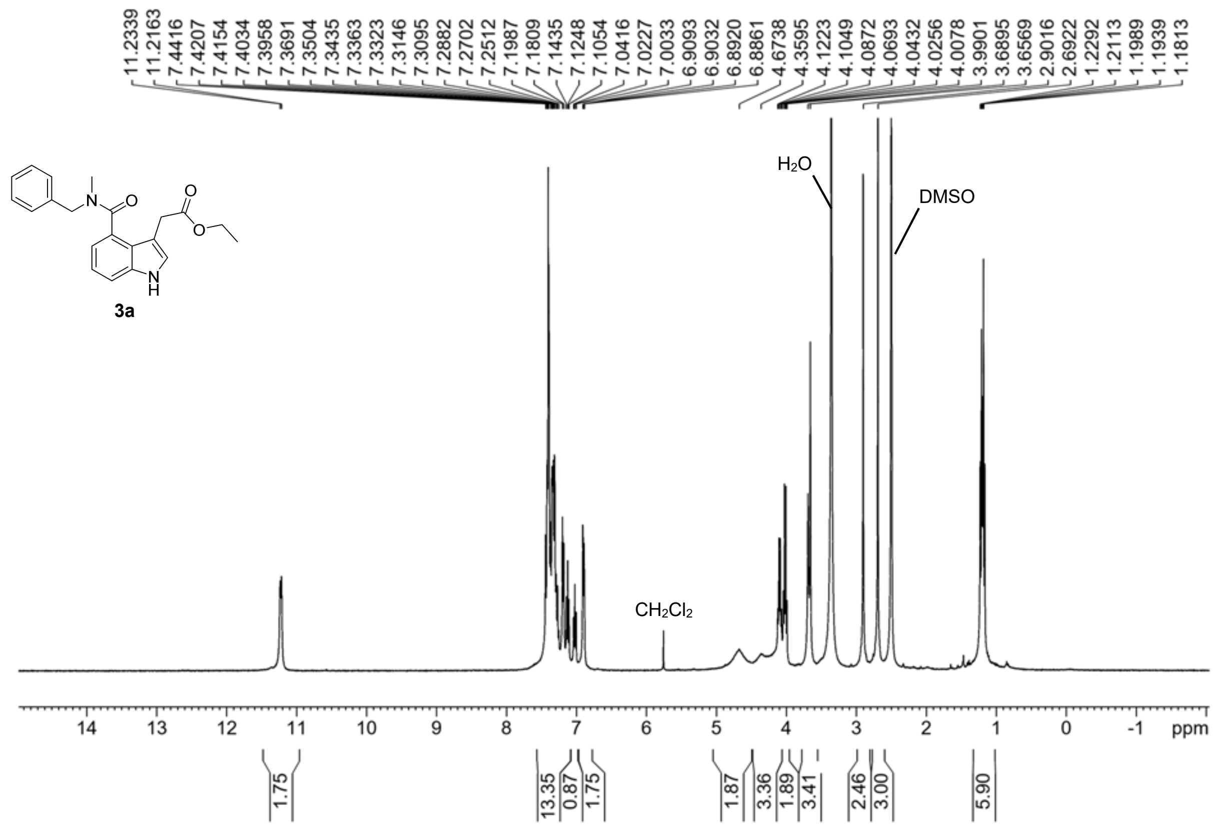
11.2339 11.2163 7.4416 7.4207 7.4154 7.4034 7.3958 7.3691 7.3504 7.3435 7.3363 7.3323 7.3146 7.3095 7.2882 7.2702 7.2512 7.1987 7.1809 7.1435 7.1248 7.1054 7.0416 7.0227 7.0033 6.9093 6.9032 6.8920 6.8861 4.6738 4.3595 4.1223 4.1049 4.0872 4.0693 4.0432 4.0256 4.0078 3.9901 3.6895 3.6569 2.9016 2.6922 1.2292 1.2113 1.1989 1.1939 1.1813
H2O
DMSO
CH2Cl2
14 13 12 11 10 9 8 7 6 5 4 3 2 1 0 -1 ppm
1.75 13.35 0.87 1.75 1.87 3.36 1.89 3.41 2.46 3.00 5.90

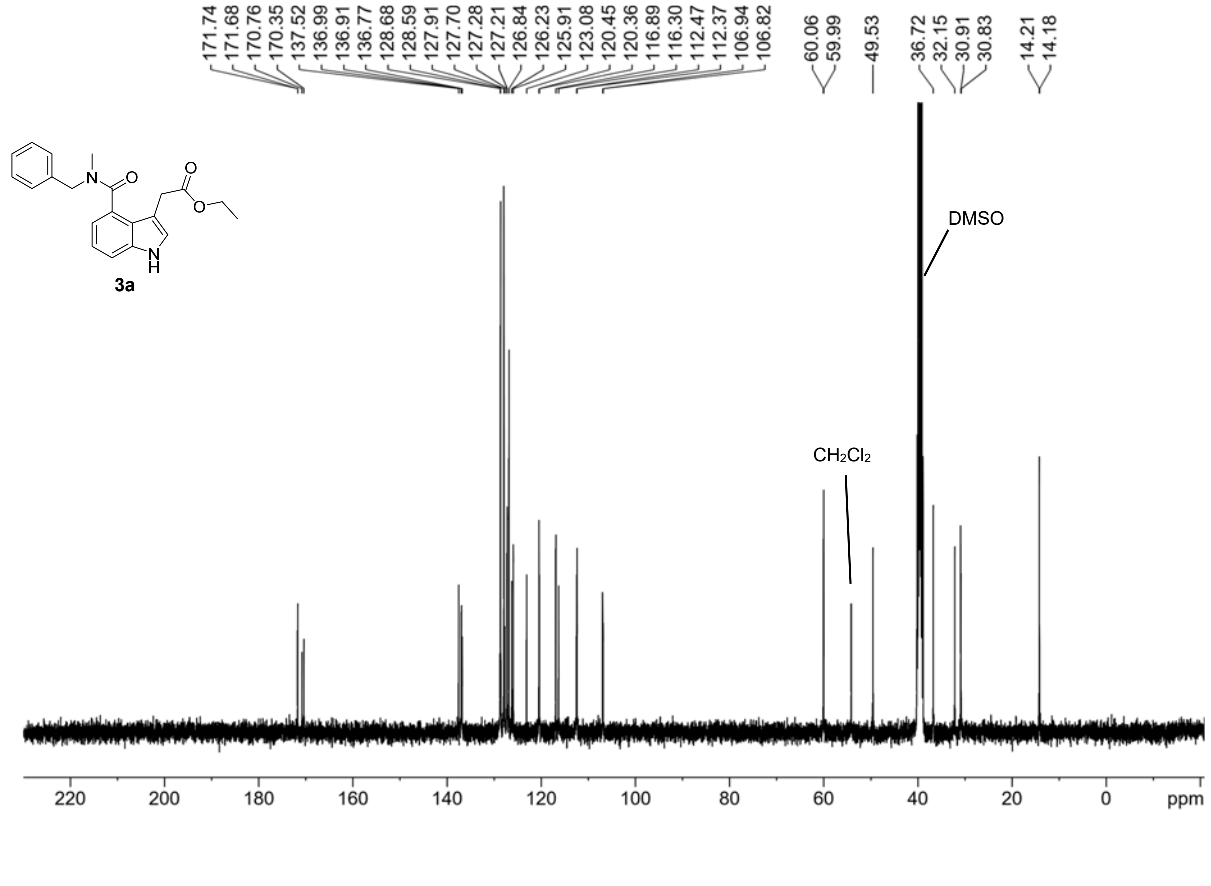
171.74 171.68 170.76 170.35 137.52 136.99 136.91 136.77 128.68 128.59 127.91 127.70 127.28 127.21 126.84 126.23 125.91 123.08 120.45 120.36 116.89 116.30 112.47 112.37 106.94 106.82 60.06 59.99 49.53 36.72 32.15 30.91 30.83 14.21 14.18
DMSO
CH2Cl2
220 200 180 160 140 120 100 80 60 40 20 0 ppm

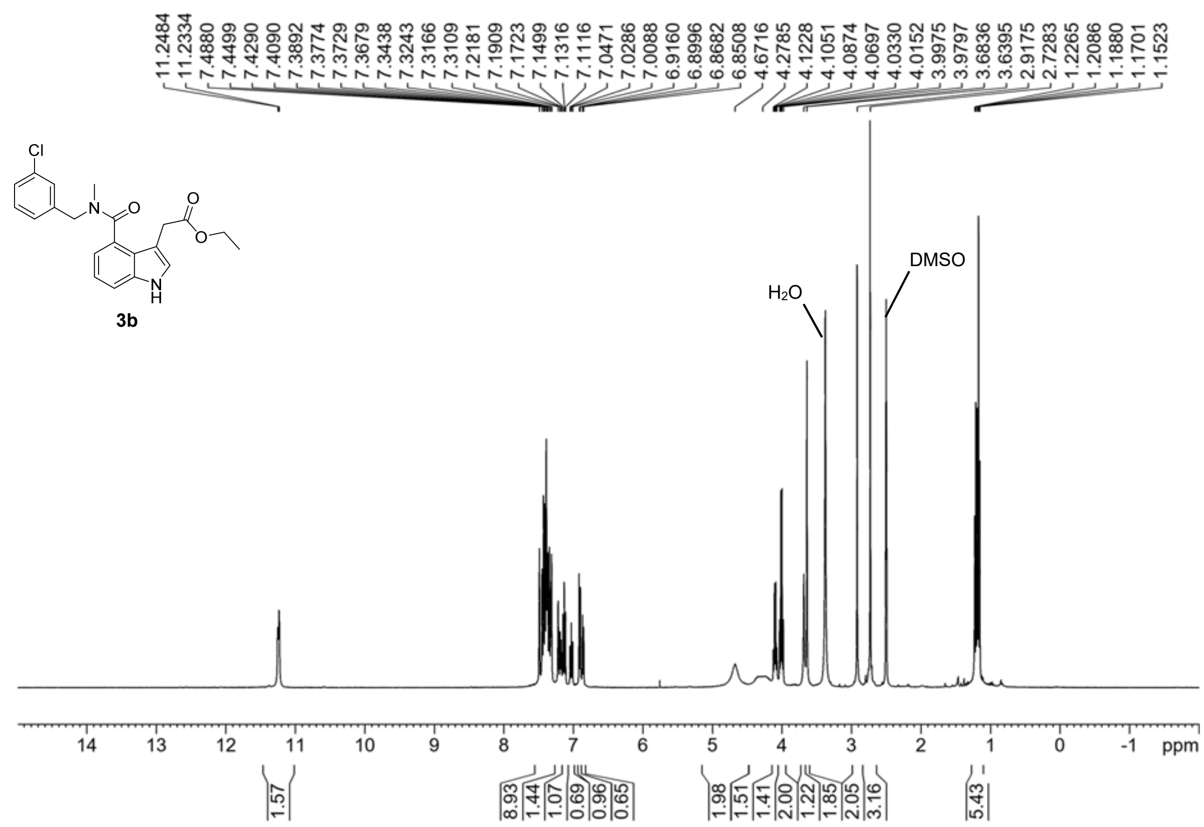
11.2484
11.2334
7.4880
7.4499
7.4290
7.4090
7.3892
7.3774
7.3729
7.3679
7.3438
7.3243
7.3166
7.3109
7.2181
7.1909
7.1723
7.1499
7.1316
7.1116
7.0471
7.0286
7.0088
6.9160
6.8996
6.8682
6.8508
4.6716
4.2785
4.1228
4.1051
4.0874
4.0697
4.0330
4.0152
3.9975
3.9797
3.6836
3.6395
2.9175
2.7283
1.2265
1.2086
1.1880
1.1701
1.1523
Cl
O
N
O
O
N
H
3b
H2O
DMSO
14 13 12 11 10 9 8 7 6 5 4 3 2 1 0 -1 ppm
1.57
8.93
1.44
1.07
0.69
0.96
0.65
1.98
1.51
1.41
2.00
1.22
1.85
2.05
3.16
5.43

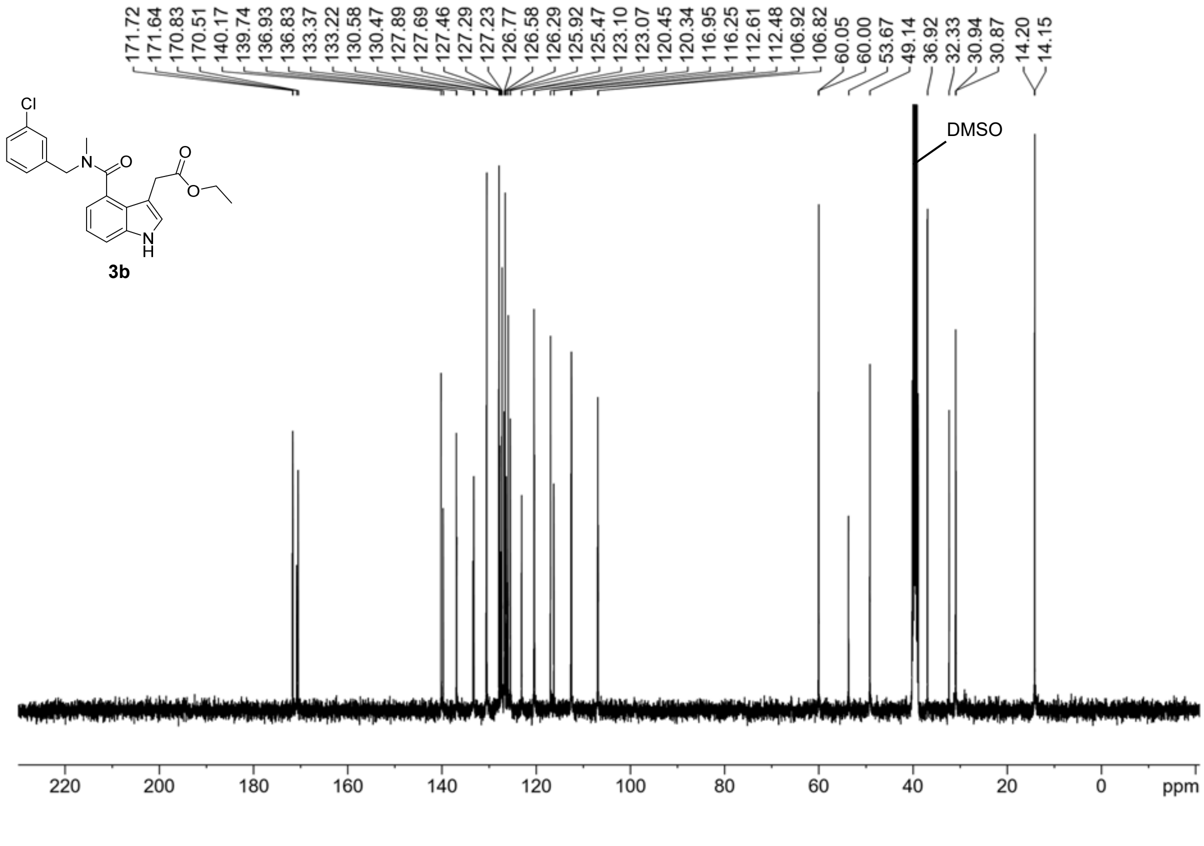
171.72
171.64
170.83
170.51
140.17
139.74
136.93
136.83
133.37
133.22
130.58
130.47
127.89
127.69
127.46
127.29
127.23
126.77
126.58
126.29
125.92
125.47
123.10
123.07
120.45
120.34
116.95
116.25
112.61
112.48
106.92
106.82
60.05
60.00
53.67
49.14
36.92
32.33
30.94
30.87
14.20
14.15
Cl
O
N
O
O
N
H
3b
DMSO
220 200 180 160 140 120 100 80 60 40 20 0 ppm

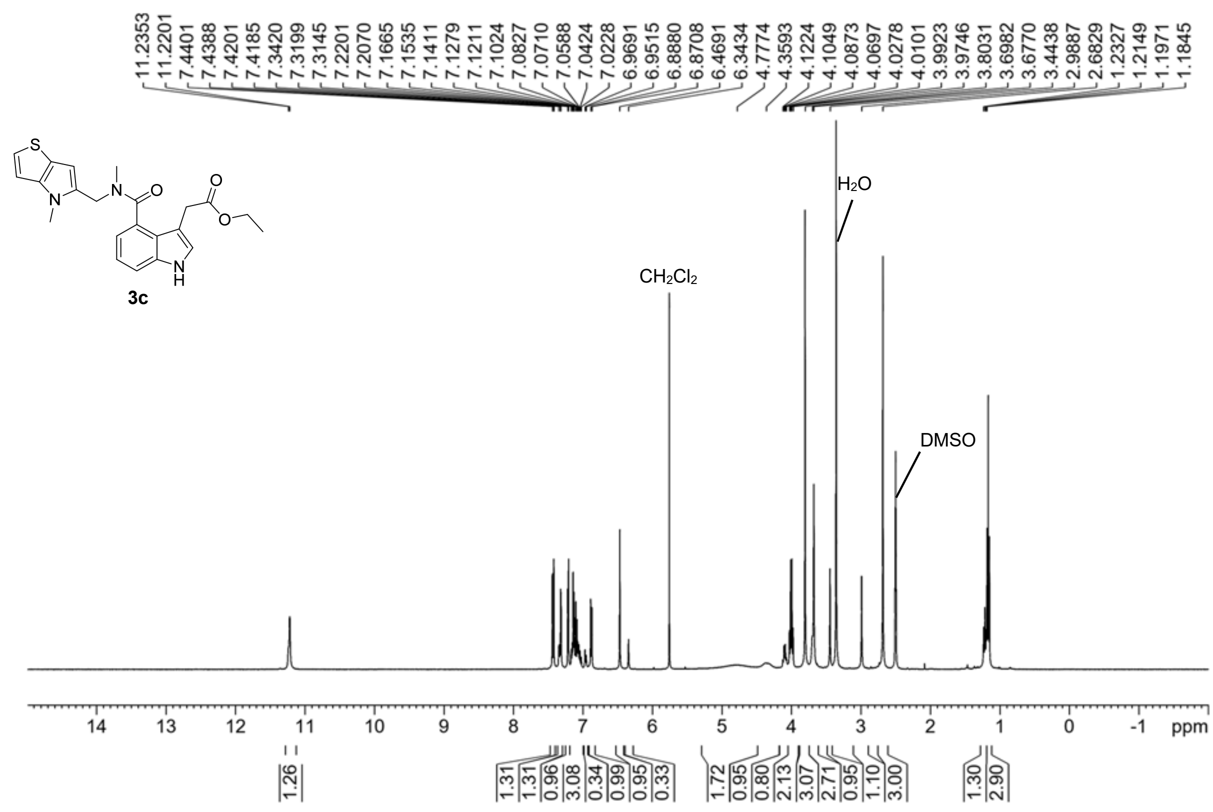
3c
CH2Cl2
H2O
DMSO
ppm

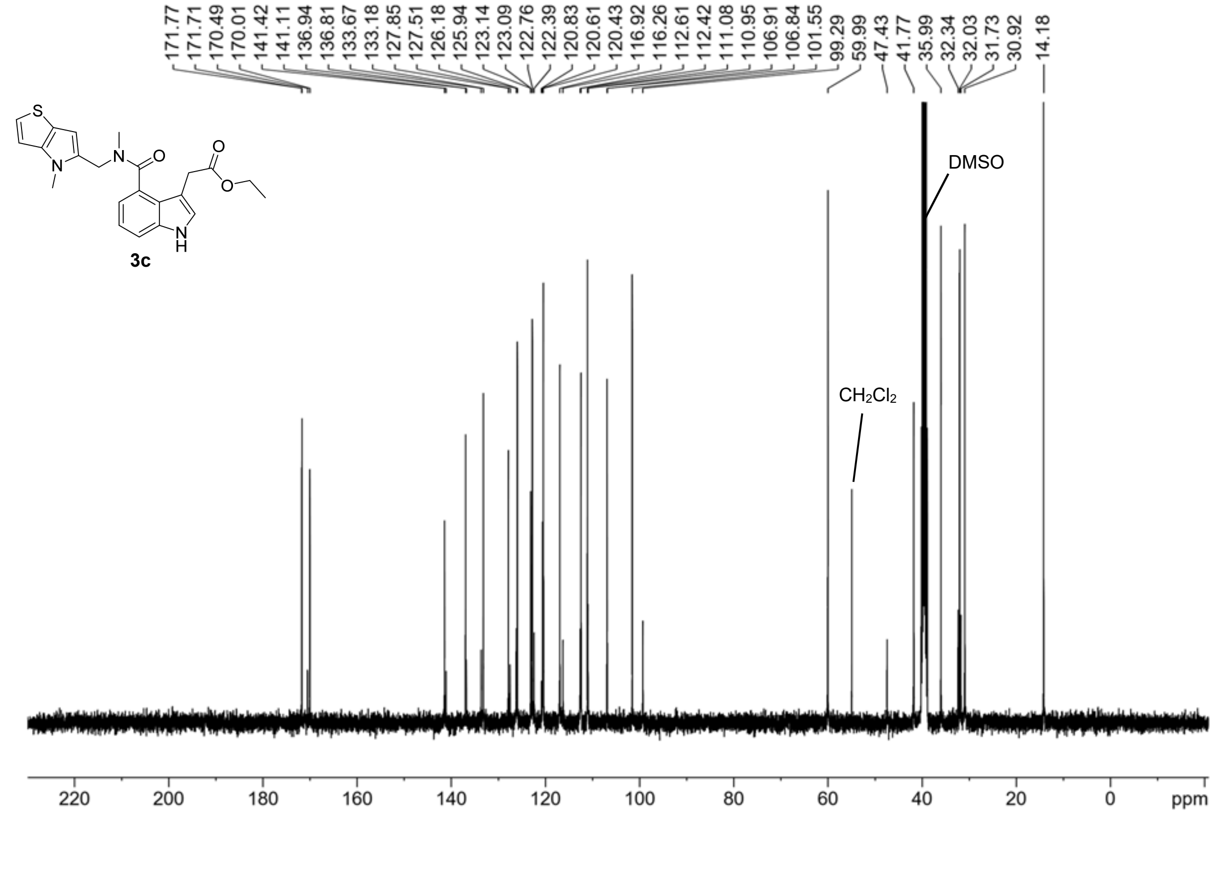
3c
DMSO
CH2Cl2
ppm

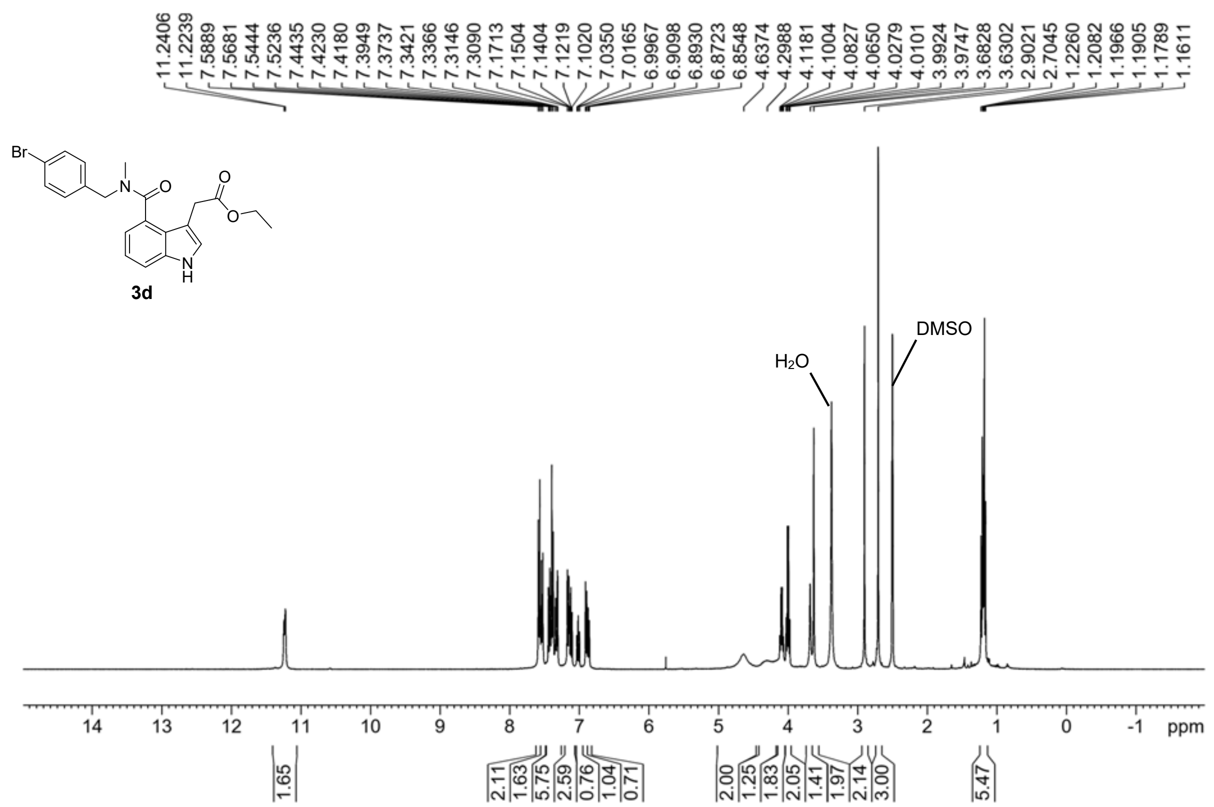
11.2406
11.2239
7.5889
7.5681
7.5444
7.5236
7.4435
7.4230
7.4180
7.3949
7.3737
7.3421
7.3366
7.3146
7.3090
7.1713
7.1504
7.1404
7.1219
7.1020
7.0350
7.0165
6.9967
6.9098
6.8930
6.8723
6.8548
4.6374
4.2988
4.1181
4.1004
4.0827
4.0650
4.0279
4.0101
3.9924
3.9747
3.6828
3.6302
2.9021
2.7045
1.2260
1.2082
1.1966
1.1905
1.1789
1.1611
Br
N
O
O
O
N
H
3d
H2O
DMSO
14
13
12
11
10
9
8
7
6
5
4
3
2
1
0
-1
ppm
1.65
2.11
1.63
5.75
2.59
0.76
1.04
0.71
2.00
1.25
1.83
2.05
1.41
1.97
2.14
3.00
5.47

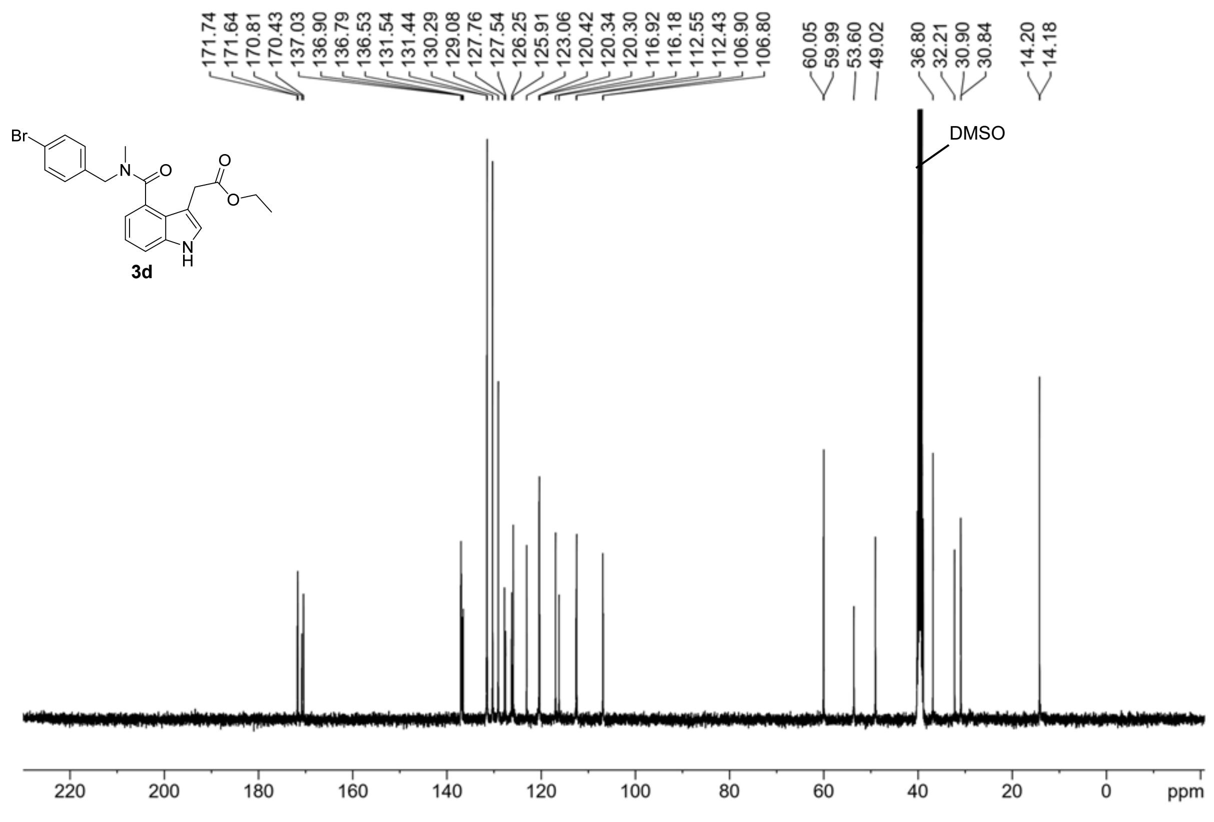
171.74
171.64
170.81
170.43
137.03
136.90
136.79
136.53
131.54
131.44
130.29
129.08
127.76
127.54
126.25
125.91
123.06
120.42
120.34
120.30
116.92
116.18
112.55
112.43
106.90
106.80
60.05
59.99
53.60
49.02
36.80
32.21
30.90
30.84
14.20
14.18
Br
N
O
O
O
N
H
3d
DMSO
220
200
180
160
140
120
100
80
60
40
20
0
ppm

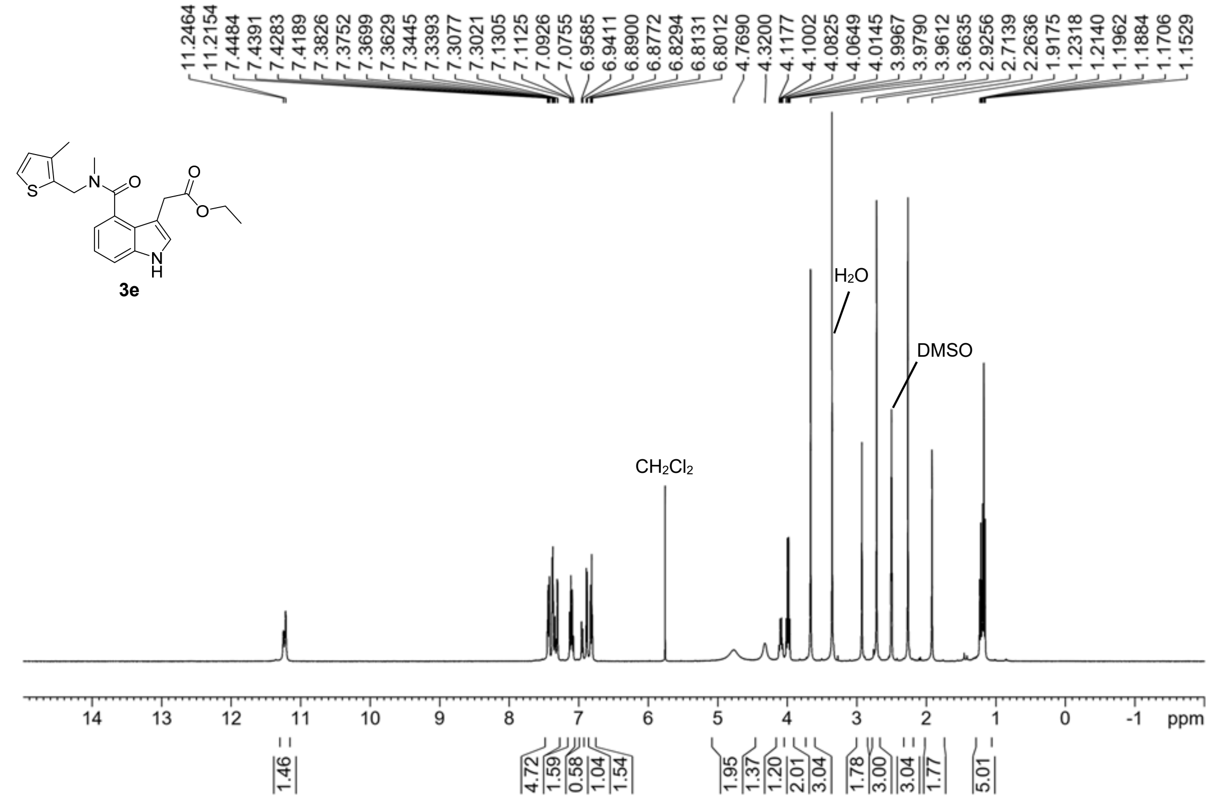
11.2464
11.2154
7.4484
7.4391
7.4283
7.4189
7.3826
7.3752
7.3699
7.3629
7.3445
7.3393
7.3077
7.3021
7.1305
7.1125
7.0926
7.0755
6.9585
6.9411
6.8900
6.8772
6.8294
6.8131
6.8012
4.7690
4.3200
4.1177
4.1002
4.0825
4.0649
4.0145
3.9967
3.9790
3.9612
3.6635
2.9256
2.7139
2.2636
1.9175
1.2318
1.2140
1.1962
1.1884
1.1706
1.1529
3e
H_2O
DMSO
CH_2Cl_2
14 13 12 11 10 9 8 7 6 5 4 3 2 1 0 -1 ppm
1.46
4.72
1.59
0.58
1.04
1.54
1.95
1.37
1.20
2.01
3.04
1.78
3.00
3.04
1.77
5.01

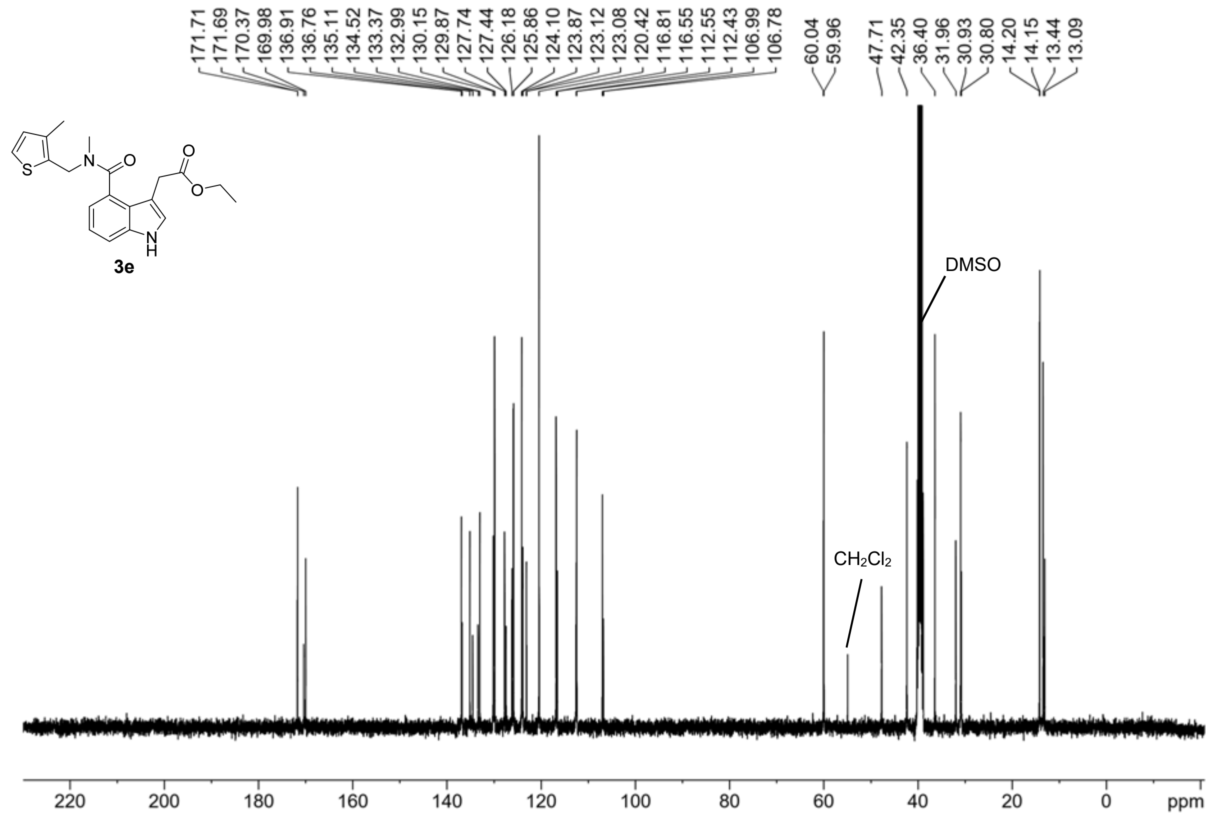
171.71
171.69
170.37
169.98
136.91
136.76
135.11
134.52
133.37
132.99
130.15
129.87
127.74
127.44
126.18
125.86
124.10
123.87
123.12
123.08
120.42
116.81
116.55
112.55
112.43
106.99
106.78
60.04
59.96
47.71
42.35
36.40
31.96
30.93
30.80
14.20
14.15
13.44
13.09
3e
DMSO
CH_2Cl_2
220 200 180 160 140 120 100 80 60 40 20 0 ppm

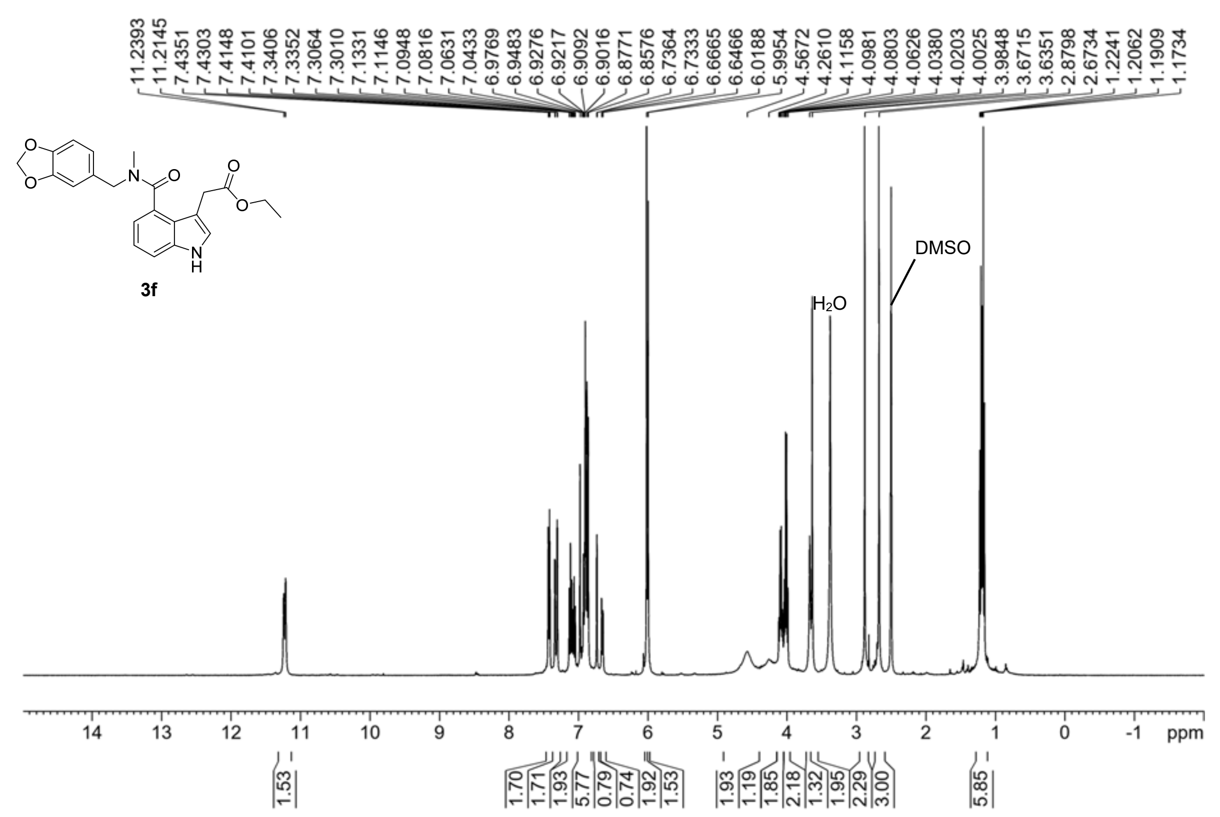
11.2393 11.2145 7.4351 7.4303 7.4148 7.4101 7.3406 7.3352 7.3064 7.3010 7.1331 7.1146 7.0948 7.0816 7.0631 7.0433 6.9769 6.9483 6.9276 6.9217 6.9092 6.9016 6.8771 6.8576 6.7364 6.7333 6.6665 6.6466 6.0188 5.9954 4.5672 4.2610 4.1158 4.0981 4.0803 4.0626 4.0380 4.0203 4.0025 3.9848 3.6715 3.6351 2.8798 2.6734 1.2241 1.2062 1.1909 1.1734
3f
DMSO
H_2O
14 13 12 11 10 9 8 7 6 5 4 3 2 1 0 -1 ppm
1.53 1.70 1.71 1.93 5.77 0.79 0.74 1.92 1.53 1.93 1.19 1.85 2.18 1.32 1.95 2.29 3.00 5.85

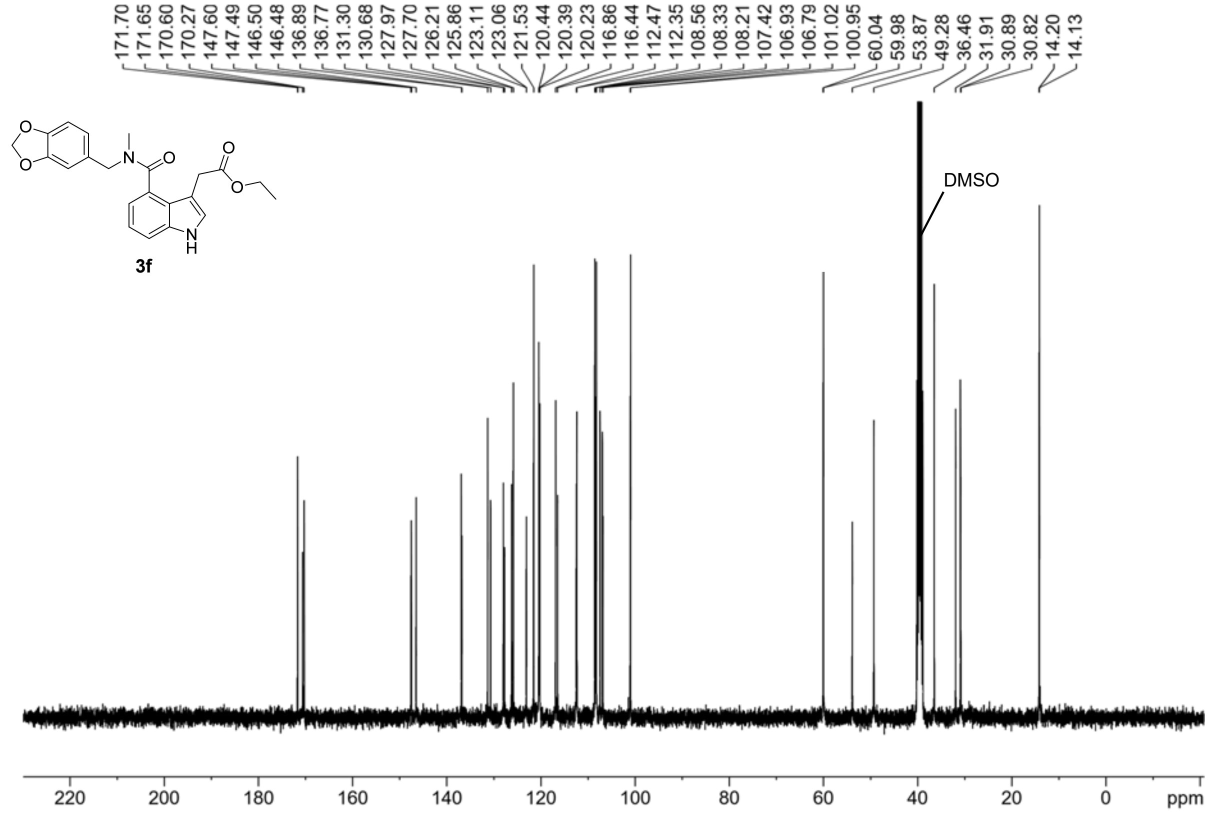
171.70 171.65 170.60 170.27 147.60 147.49 146.50 146.48 136.89 136.77 131.30 130.68 127.97 127.70 126.21 125.86 123.11 123.06 121.53 120.44 120.39 120.23 116.86 116.44 112.47 112.35 108.56 108.33 108.21 107.42 106.93 106.79 101.02 100.95 60.04 59.98 53.87 49.28 36.46 31.91 30.89 30.82 14.20 14.13
3f
DMSO
220 200 180 160 140 120 100 80 60 40 20 0 ppm

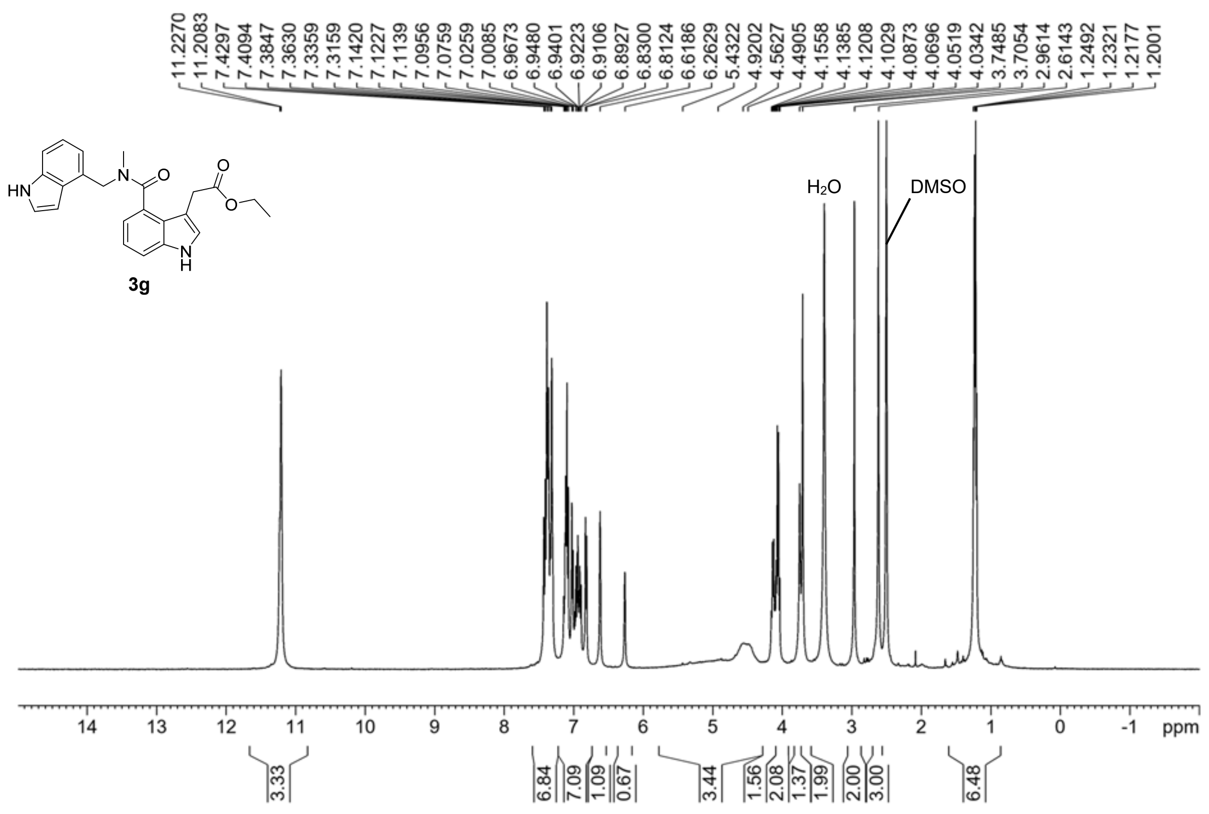
11.2270
11.2083
7.4297
7.4094
7.3847
7.3630
7.3359
7.3159
7.1420
7.1227
7.1139
7.0956
7.0759
7.0259
7.0085
6.9673
6.9480
6.9401
6.9223
6.9106
6.8927
6.8300
6.8124
6.6186
6.2629
5.4322
4.9202
4.5627
4.4905
4.1558
4.1385
4.1208
4.1029
4.0873
4.0696
4.0519
4.0342
3.7485
3.7054
2.9614
2.6143
1.2492
1.2321
1.2177
1.2001
HN
O
O
O
N
H
3g
H2O
DMSO
14
13
12
11
10
9
8
7
6
5
4
3
2
1
0
-1
ppm
3.33
6.84
7.09
1.09
0.67
3.44
1.56
2.08
1.37
1.99
2.00
3.00
6.48

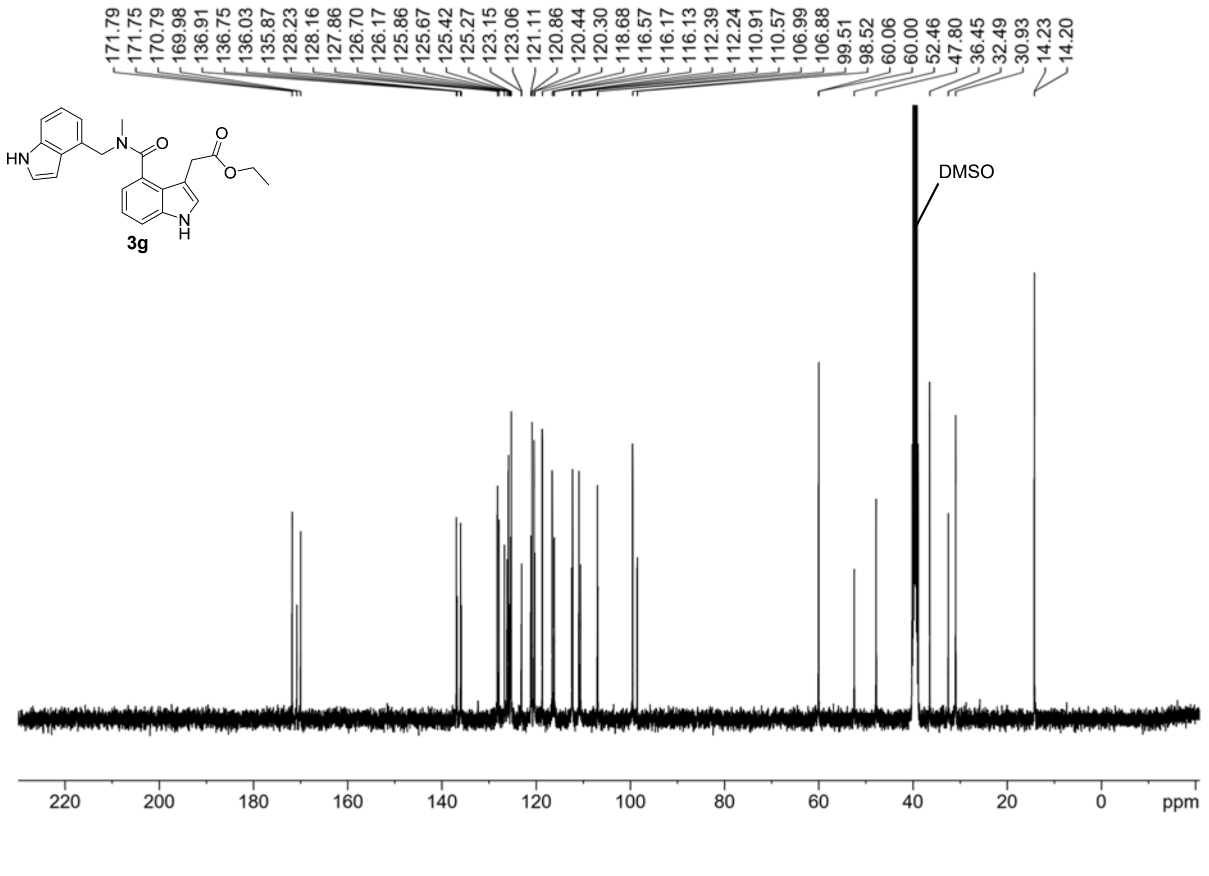
171.79
171.75
170.79
169.98
136.91
136.75
136.03
135.87
128.23
128.16
127.86
126.70
126.17
125.86
125.67
125.42
125.27
123.15
123.06
121.11
120.86
120.44
120.30
118.68
116.57
116.17
116.13
112.39
112.24
110.91
110.57
106.99
106.88
99.51
98.52
60.06
60.00
52.46
47.80
36.45
32.49
30.93
14.23
14.20
HN
O
O
O
N
H
3g
DMSO
220
200
180
160
140
120
100
80
60
40
20
0
ppm

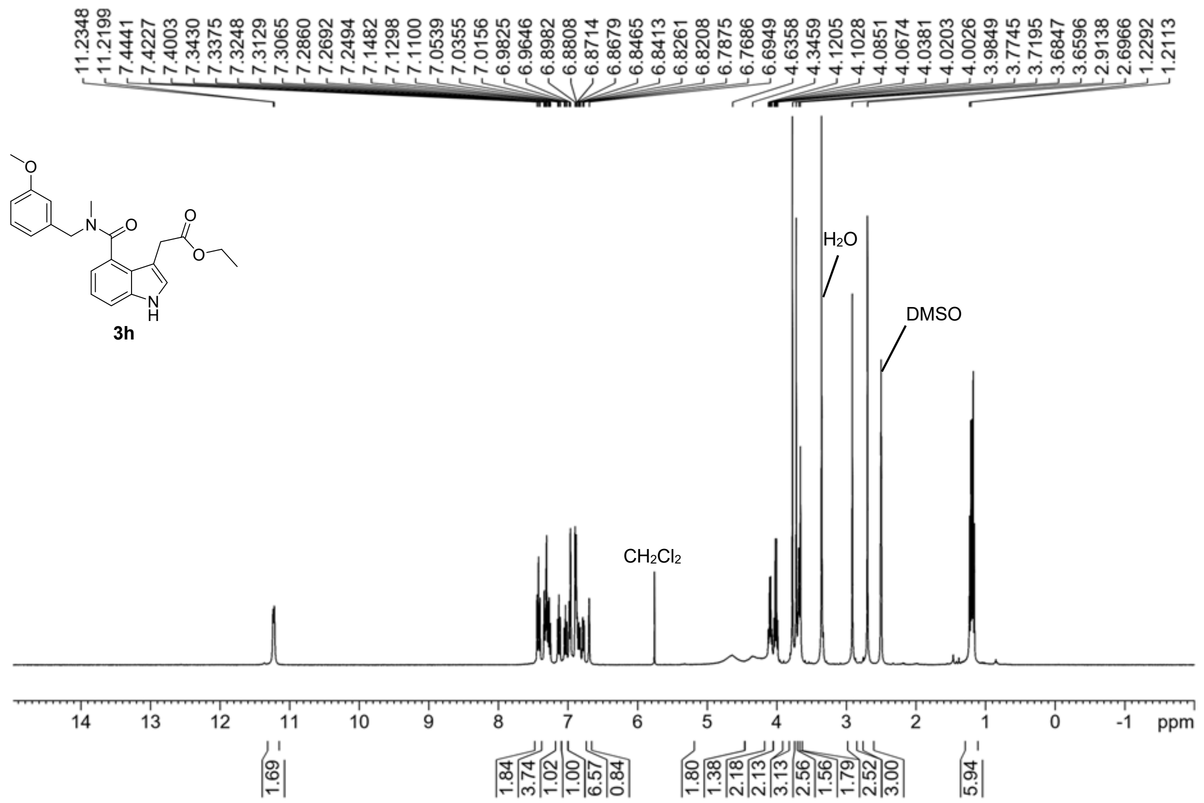
11.2348 11.2199 7.4441 7.4227 7.4003 7.3430 7.3375 7.3248 7.3129 7.3065 7.2860 7.2692 7.2494 7.1482 7.1298 7.1100 7.0539 7.0355 7.0156 6.9825 6.9646 6.8982 6.8808 6.8714 6.8679 6.8465 6.8413 6.8261 6.8208 6.7875 6.7686 6.6949 4.6358 4.3459 4.1205 4.1028 4.0851 4.0674 4.0381 4.0203 4.0026 3.9849 3.7745 3.7195 3.6847 3.6596 2.9138 2.6966 1.2292 1.2113
3h
H2O
DMSO
CH2Cl2
14 13 12 11 10 9 8 7 6 5 4 3 2 1 0 -1 ppm
1.69 1.84 3.74 1.02 1.00 6.57 0.84 1.80 1.38 2.18 2.13 3.13 2.56 1.56 1.79 2.52 3.00 5.94

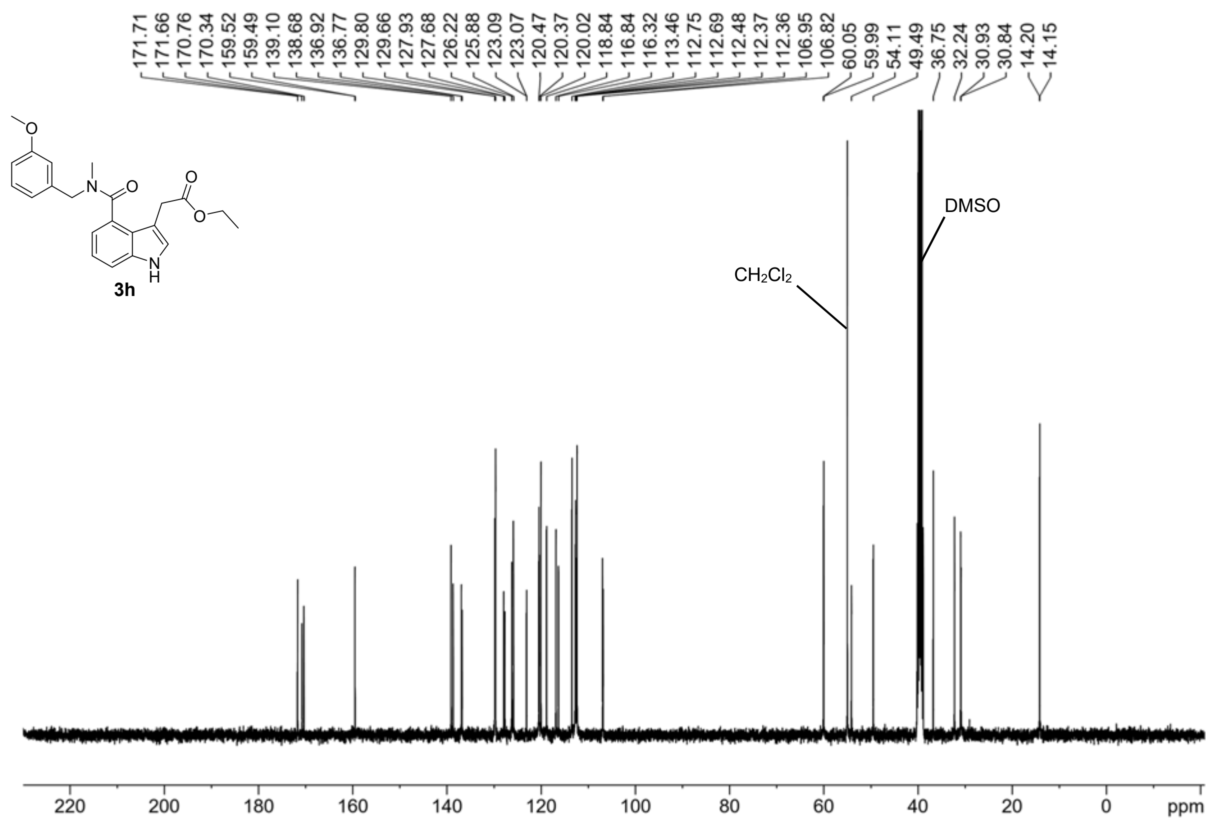
171.71 171.66 170.76 170.34 159.52 159.49 139.10 138.68 136.92 136.77 129.80 129.66 127.93 127.68 126.22 125.88 123.09 123.07 120.47 120.37 120.02 118.84 116.84 116.32 113.46 112.75 112.69 112.48 112.37 112.36 106.95 106.82 60.05 59.99 54.11 49.49 36.75 32.24 30.93 30.84 14.20 14.15
3h
DMSO
CH2Cl2
220 200 180 160 140 120 100 80 60 40 20 0 ppm

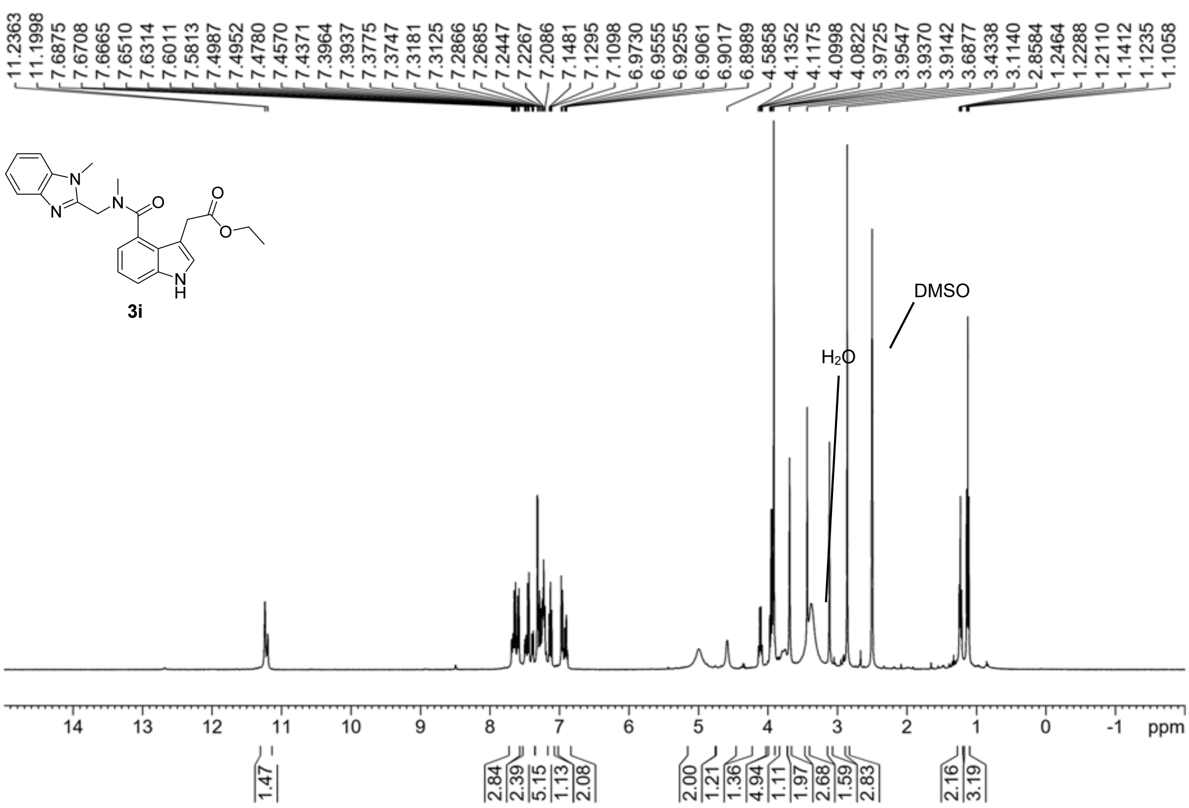
11.2363
11.1998
7.6875
7.6708
7.6665
7.6510
7.6314
7.6011
7.5813
7.4987
7.4952
7.4780
7.4570
7.4371
7.3964
7.3937
7.3775
7.3747
7.3181
7.3125
7.2866
7.2685
7.2447
7.2267
7.2086
7.1481
7.1295
7.1098
6.9730
6.9555
6.9255
6.9061
6.9017
6.8989
4.5858
4.1352
4.1175
4.0998
4.0822
3.9725
3.9547
3.9370
3.9142
3.6877
3.4338
3.1140
2.8584
1.2464
1.2288
1.2110
1.1412
1.1235
1.1058
3i
DMSO
H_2O
14 13 12 11 10 9 8 7 6 5 4 3 2 1 0 -1 ppm
1.47
2.84
2.39
5.15
1.13
2.08
2.00
1.21
1.36
4.94
1.11
1.97
2.68
1.59
2.83
2.16
3.19

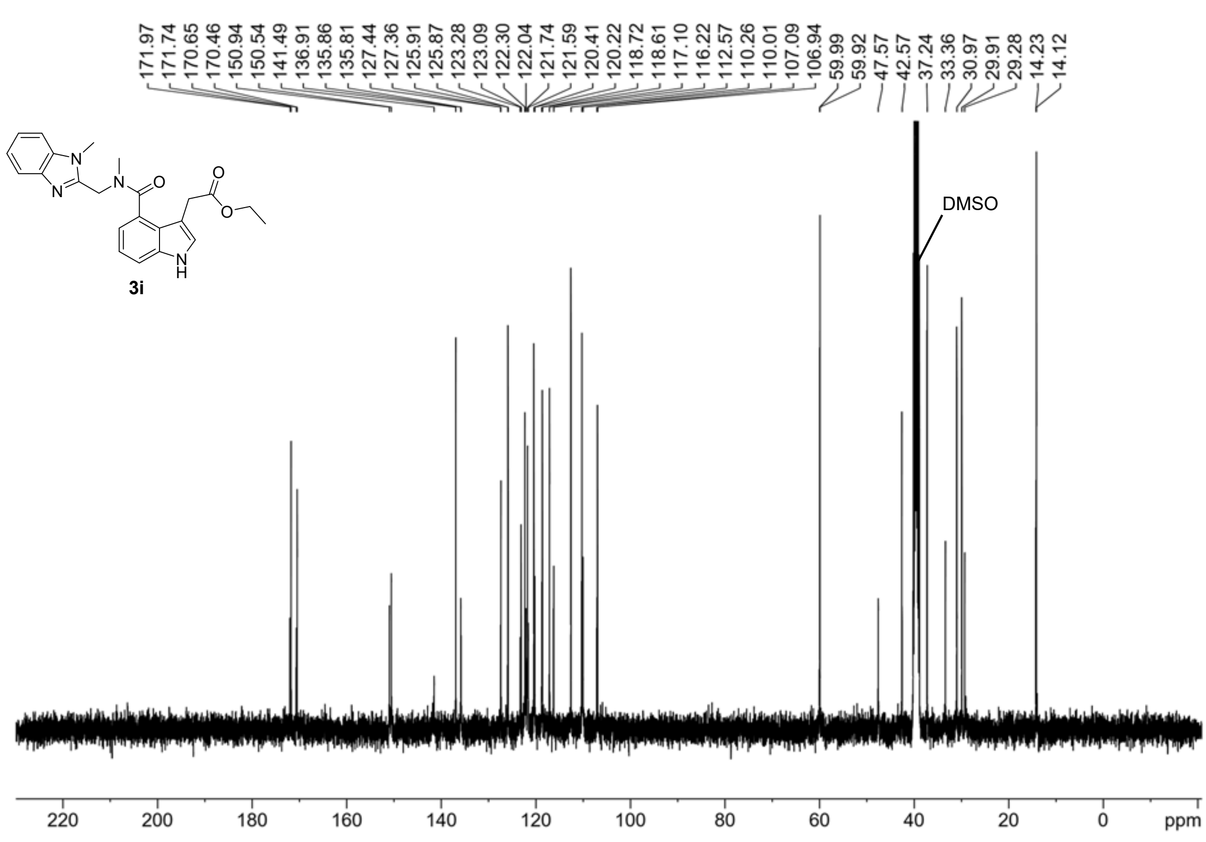
171.97
171.74
170.65
170.46
150.94
150.54
141.49
136.91
135.86
135.81
127.44
127.36
125.91
125.87
123.28
123.09
122.30
122.04
121.74
121.59
120.41
120.22
118.72
118.61
117.10
116.22
112.57
110.26
110.01
107.09
106.94
59.99
59.92
47.57
42.57
37.24
33.36
30.97
29.91
29.28
14.23
14.12
3i
DMSO
220 200 180 160 140 120 100 80 60 40 20 0 ppm

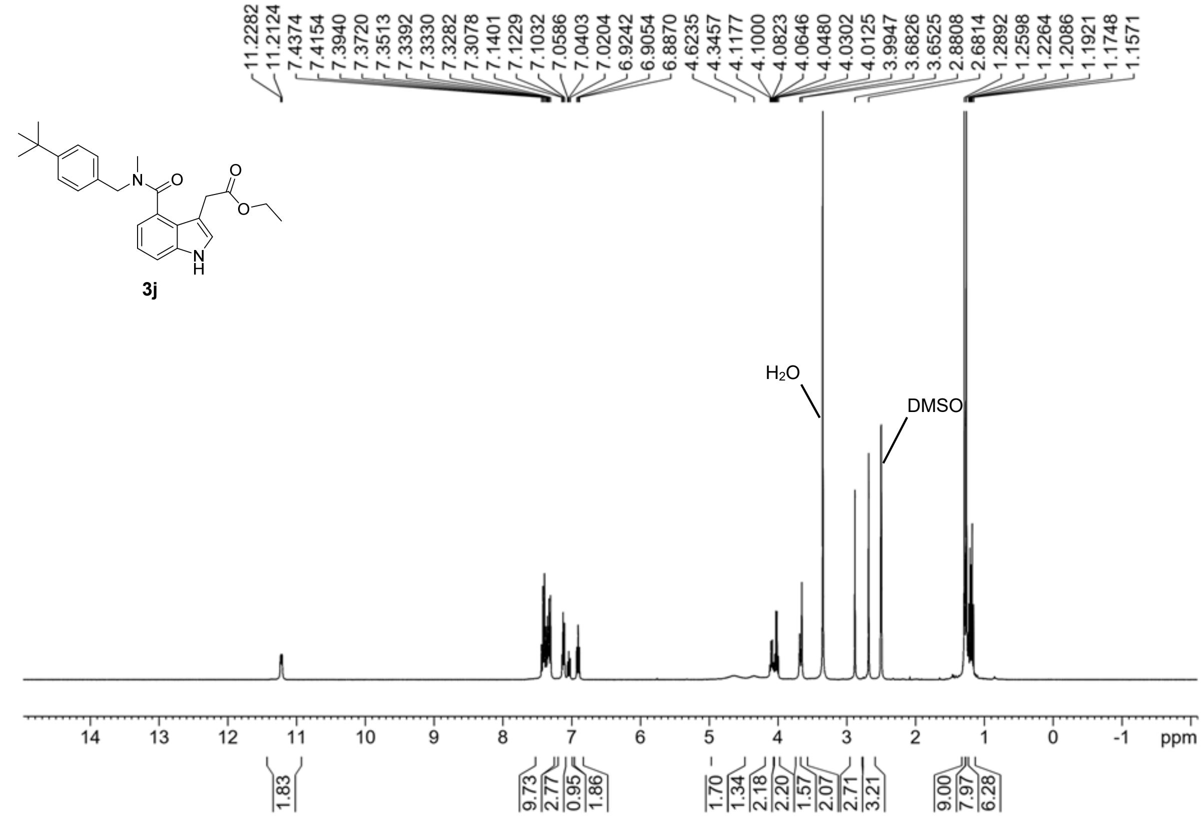

3j
11.2282
11.2124
7.4374
7.4154
7.3940
7.3720
7.3513
7.3392
7.3330
7.3282
7.3078
7.1401
7.1229
7.1032
7.0586
7.0403
7.0204
6.9242
6.9054
6.8870
4.6235
4.3457
4.1177
4.1000
4.0823
4.0646
4.0480
4.0302
4.0125
3.9947
3.6826
3.6525
2.8808
2.6814
1.2892
1.2598
1.2264
1.2086
1.1921
1.1748
1.1571
H_2O
DMSO
14 13 12 11 10 9 8 7 6 5 4 3 2 1 0 -1 ppm
1.83
9.73
2.77
0.95
1.86
1.70
1.34
2.18
2.20
1.57
2.07
2.71
3.21
9.00
7.97
6.28

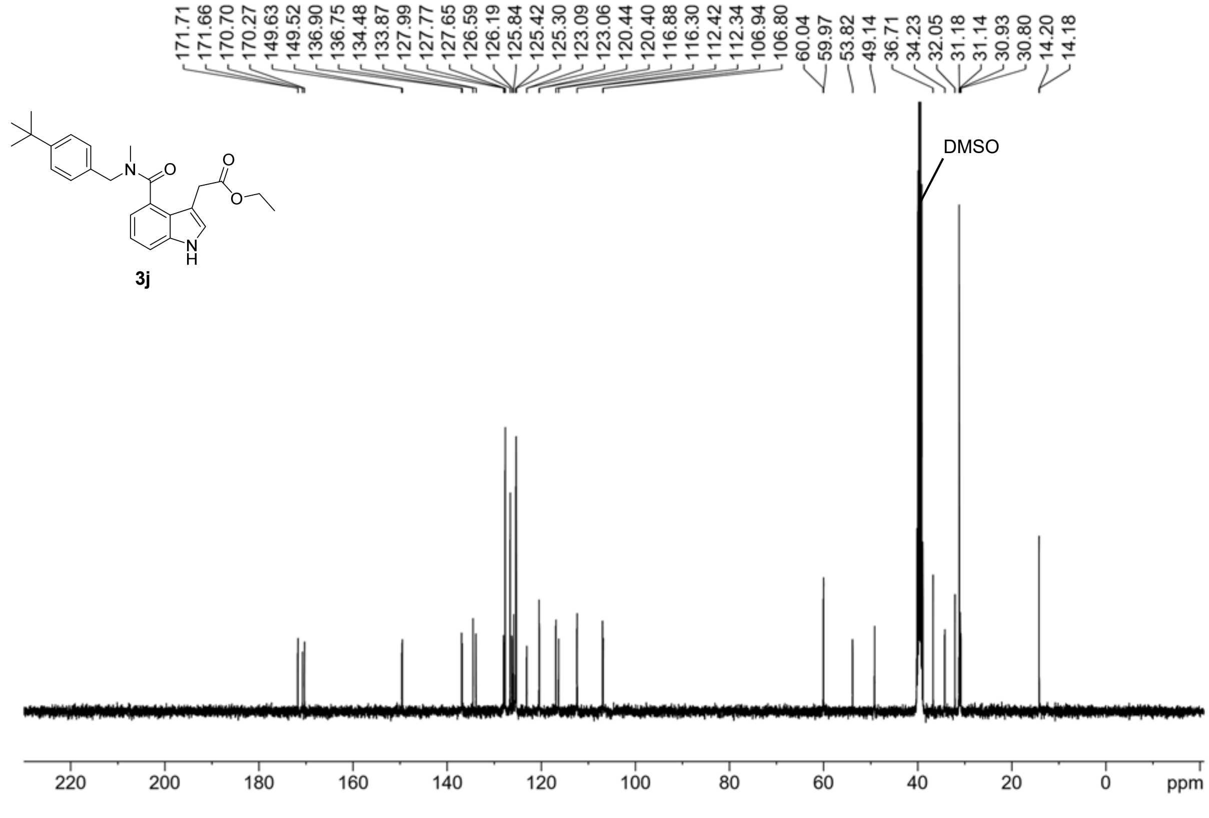

3j
171.71
171.66
170.70
170.27
149.63
149.52
136.90
136.75
134.48
133.87
127.99
127.77
127.65
126.59
126.19
125.84
125.42
125.30
123.09
123.06
120.44
120.40
116.88
116.30
112.42
112.34
106.94
106.80
60.04
59.97
53.82
49.14
36.71
34.23
32.05
31.18
31.14
30.93
30.80
14.20
14.18
DMSO
220 200 180 160 140 120 100 80 60 40 20 0 ppm

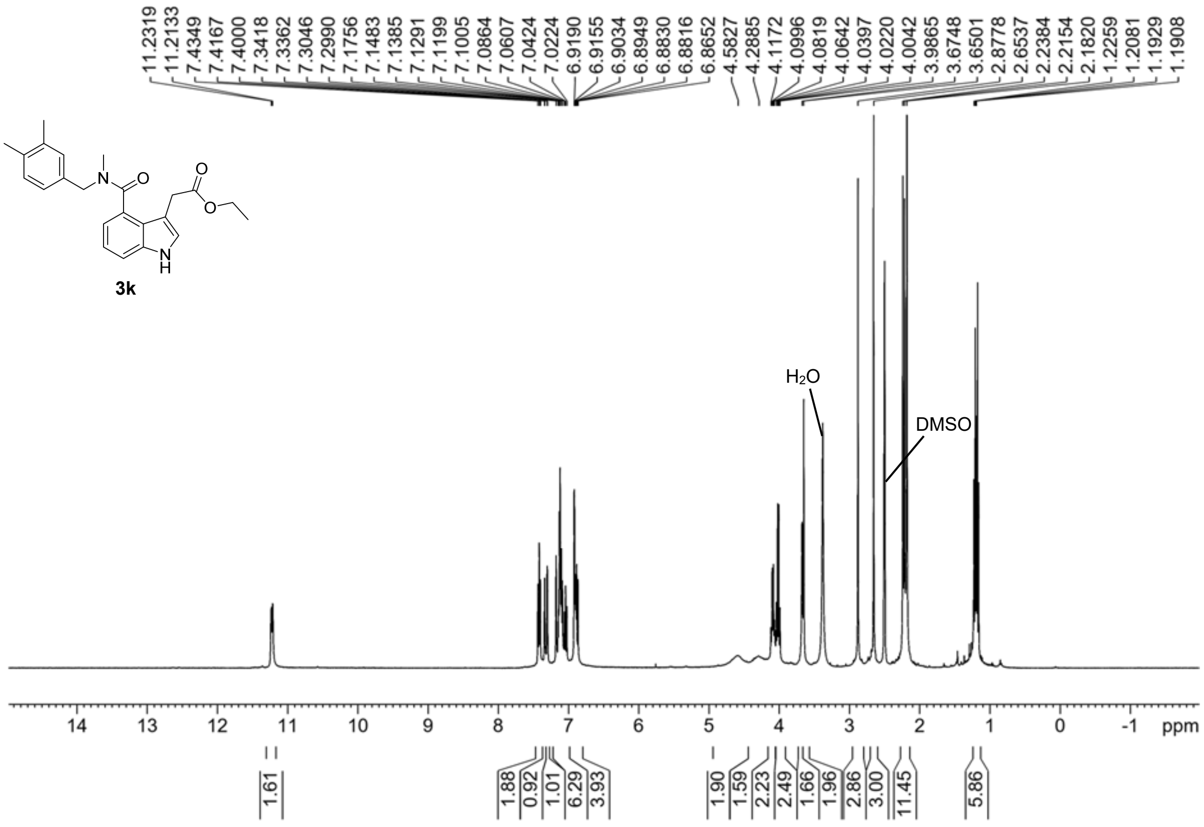

11.2319
11.2133
7.4349
7.4167
7.4000
7.3418
7.3362
7.3046
7.2990
7.1756
7.1483
7.1385
7.1291
7.1199
7.1005
7.0864
7.0607
7.0424
7.0224
6.9190
6.9155
6.9034
6.8949
6.8830
6.8816
6.8652
4.5827
4.2885
4.1172
4.0996
4.0819
4.0642
4.0397
4.0220
4.0042
3.9865
3.6748
3.6501
2.8778
2.6537
2.2384
2.2154
2.1820
1.2259
1.2081
1.1929
1.1908
3k
H_2O
DMSO
ppm
1.61
1.88
0.92
1.01
6.29
3.93
1.90
1.59
2.23
2.49
1.66
1.96
2.86
3.00
11.45
5.86

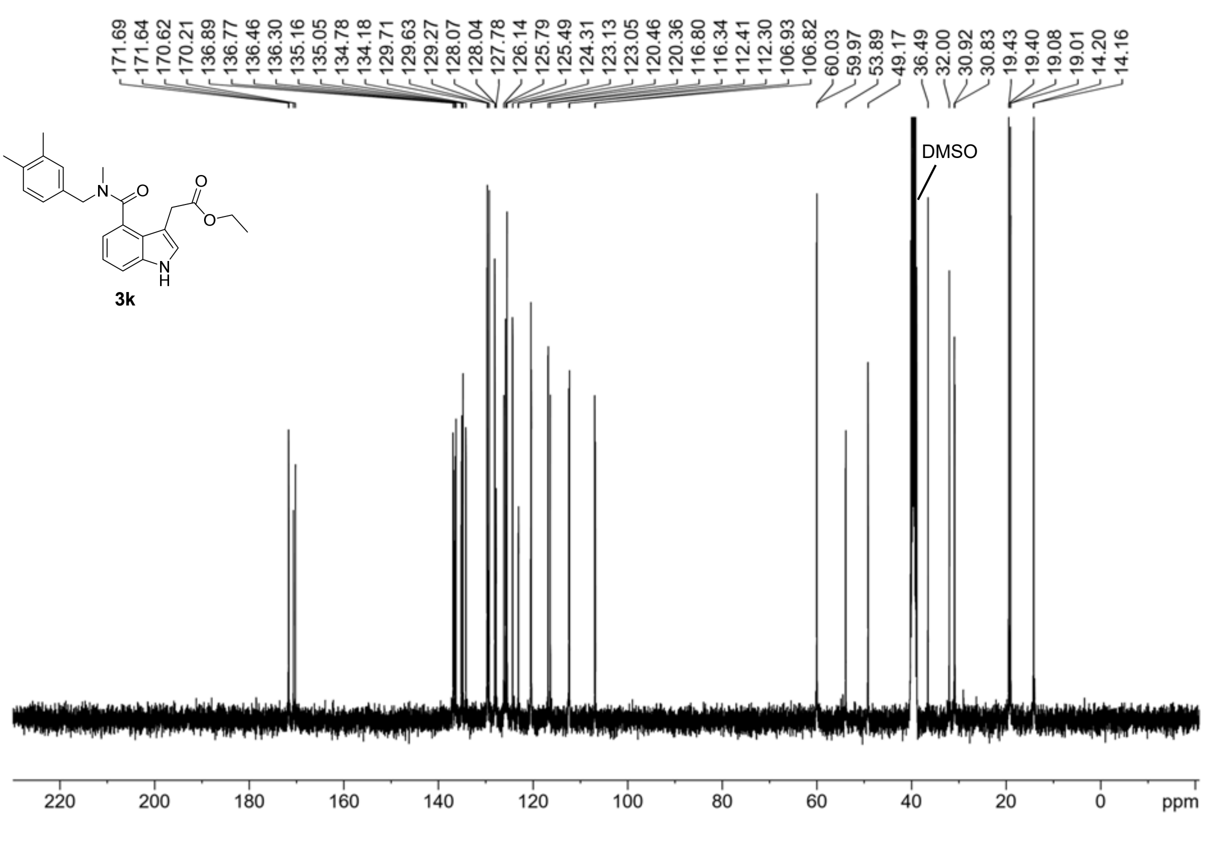

171.69
171.64
170.62
170.21
136.89
136.77
136.46
136.30
135.16
135.05
134.78
134.18
129.71
129.63
129.27
128.07
128.04
127.78
126.14
125.79
125.49
124.31
123.13
123.05
120.46
120.36
116.80
116.34
112.41
112.30
106.93
106.82
60.03
59.97
53.89
49.17
36.49
32.00
30.92
30.83
19.43
19.40
19.08
19.01
14.20
14.16
3k
DMSO
ppm

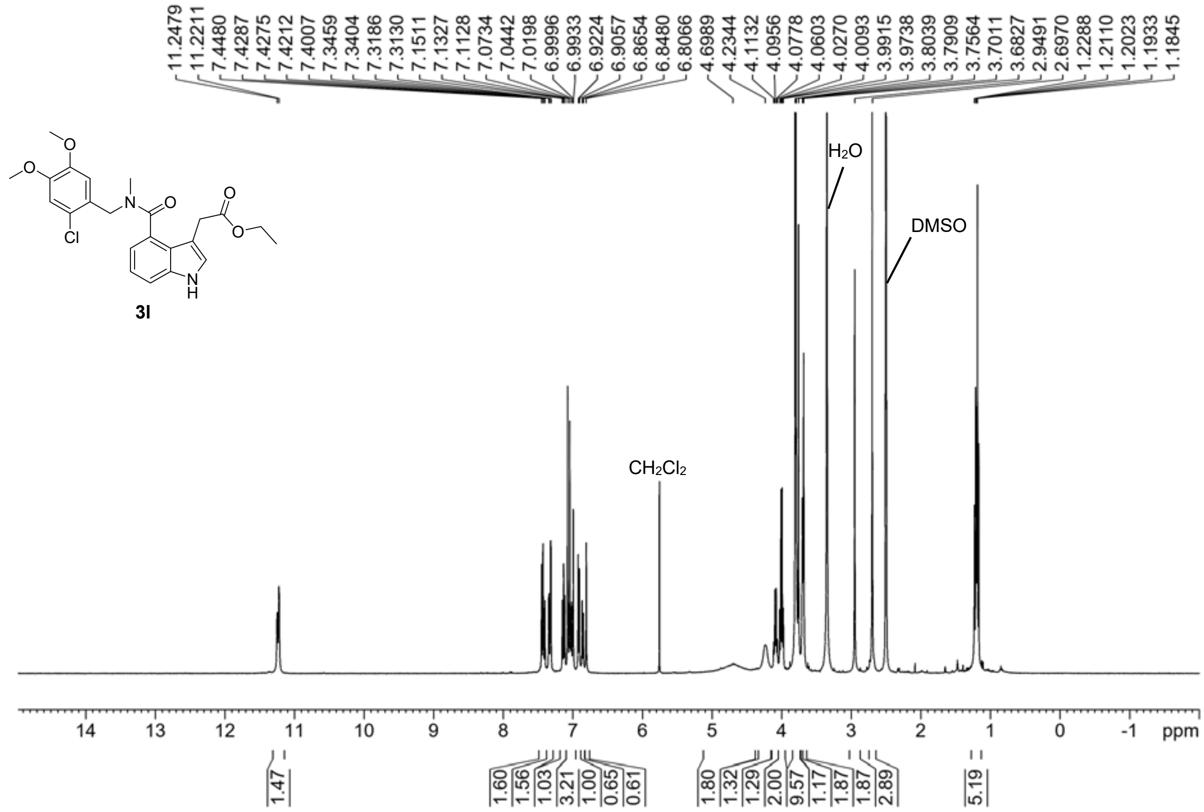
11.2479
11.2211
7.4480
7.4287
7.4275
7.4212
7.4007
7.3459
7.3404
7.3186
7.3130
7.1511
7.1327
7.1128
7.0734
7.0442
7.0198
6.9996
6.9933
6.9224
6.9057
6.8654
6.8480
6.8066
4.6989
4.2344
4.1132
4.0956
4.0778
4.0603
4.0270
4.0093
3.9915
3.9738
3.8039
3.7909
3.7564
3.7011
3.6827
2.9491
2.6970
1.2288
1.2110
1.1933
1.1845
H2O
DMSO
CH2Cl2
Cl
O
N
NH
3l
14
13
12
11
10
9
8
7
6
5
4
3
2
1
0
-1
ppm
1.47
1.60
1.56
1.03
3.21
1.00
0.65
0.61
1.80
1.32
1.29
2.00
9.57
1.17
1.87
1.87
2.89
5.19

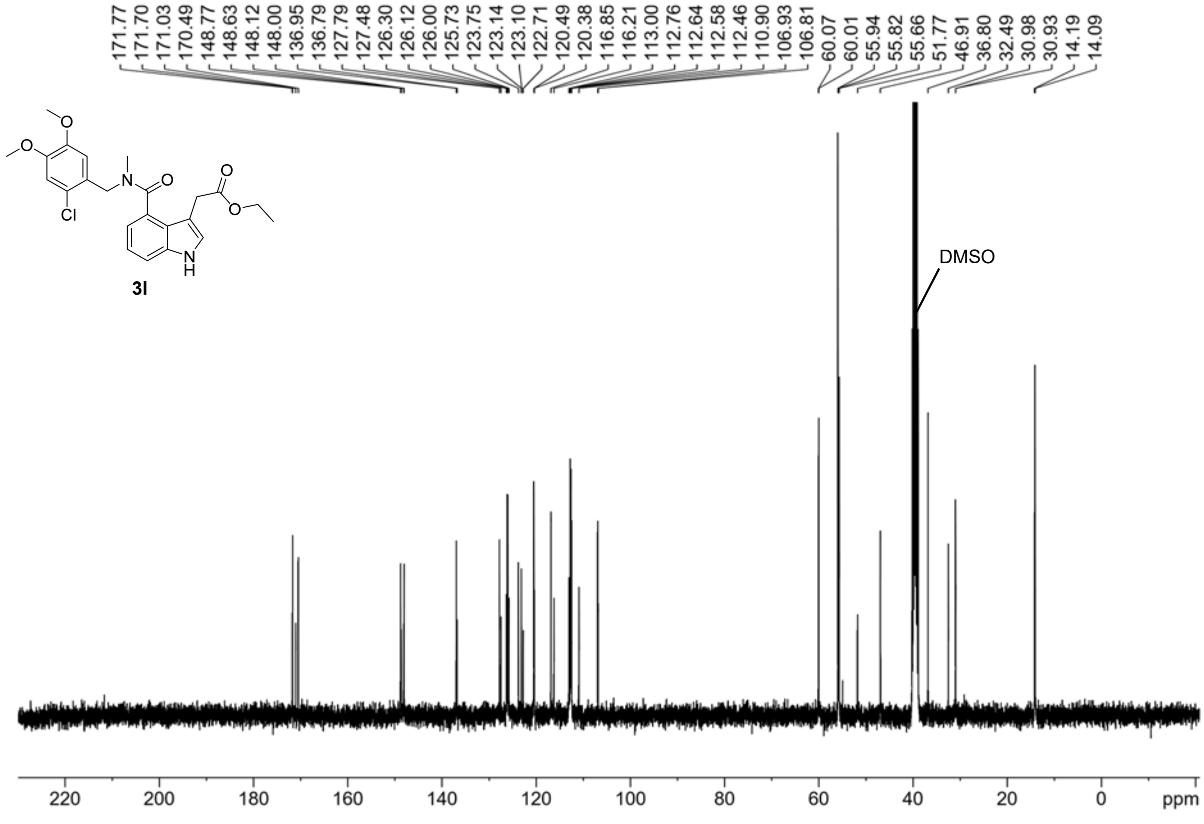
171.77
171.70
171.03
170.49
148.77
148.63
148.12
148.00
136.95
136.79
127.79
127.48
126.30
126.12
126.00
125.73
123.75
123.14
123.10
122.71
120.49
120.38
116.85
116.21
113.00
112.76
112.64
112.58
112.46
110.90
106.93
106.81
60.07
60.01
55.94
55.82
55.66
51.77
46.91
36.80
32.49
30.98
30.93
14.19
14.09
DMSO
Cl
O
N
NH
3l
220
200
180
160
140
120
100
80
60
40
20
0
ppm

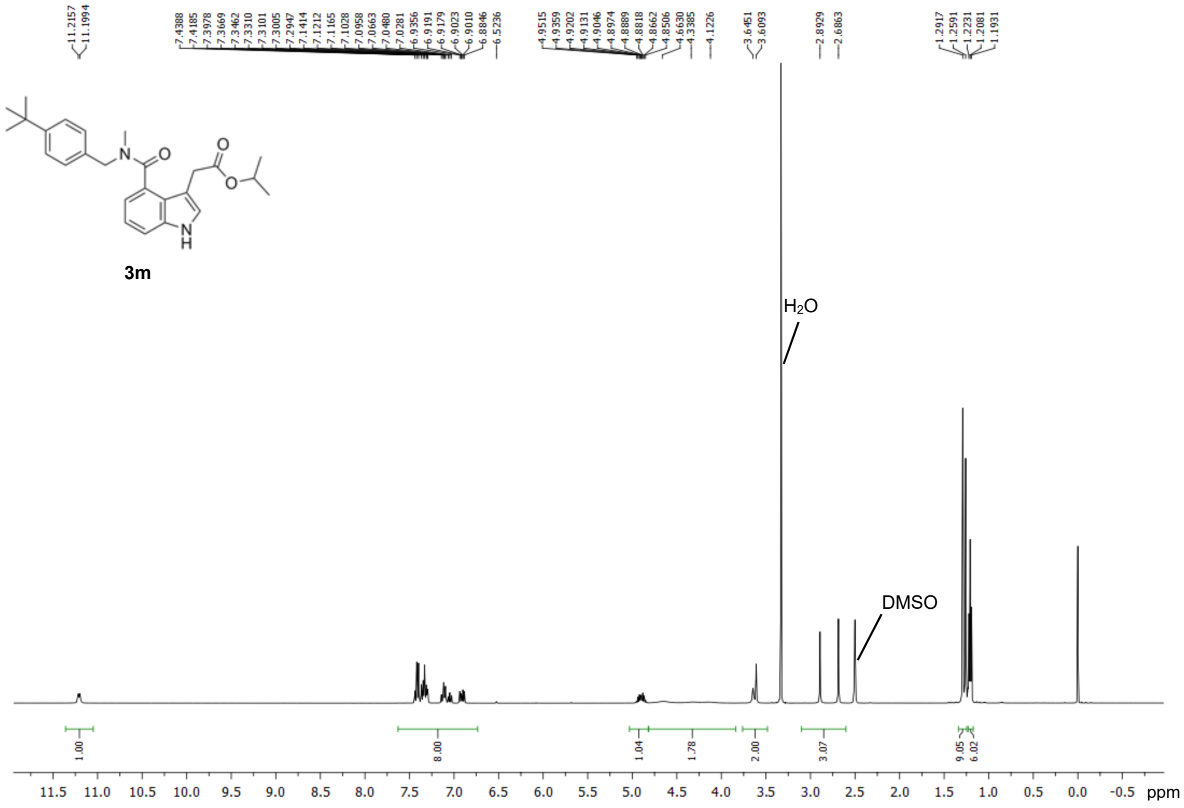
3m
H2O
DMSO
ppm

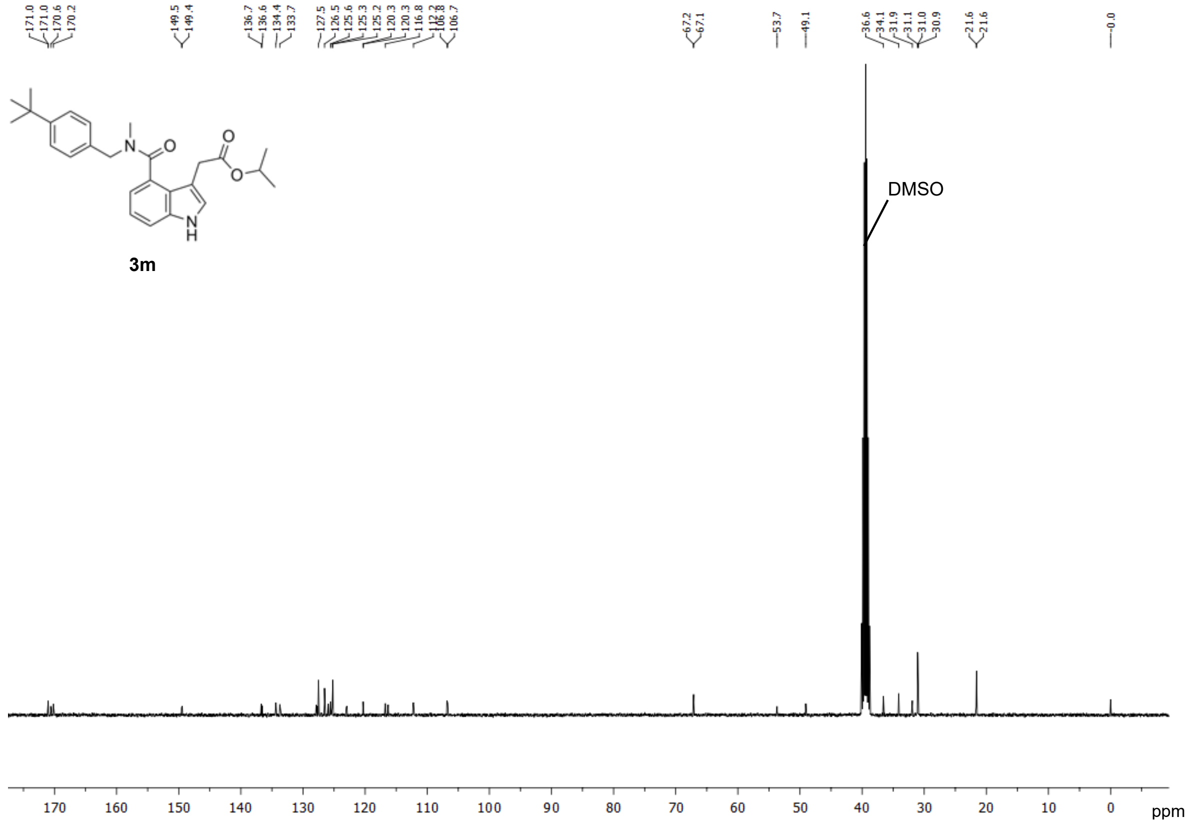
3m
DMSO
ppm

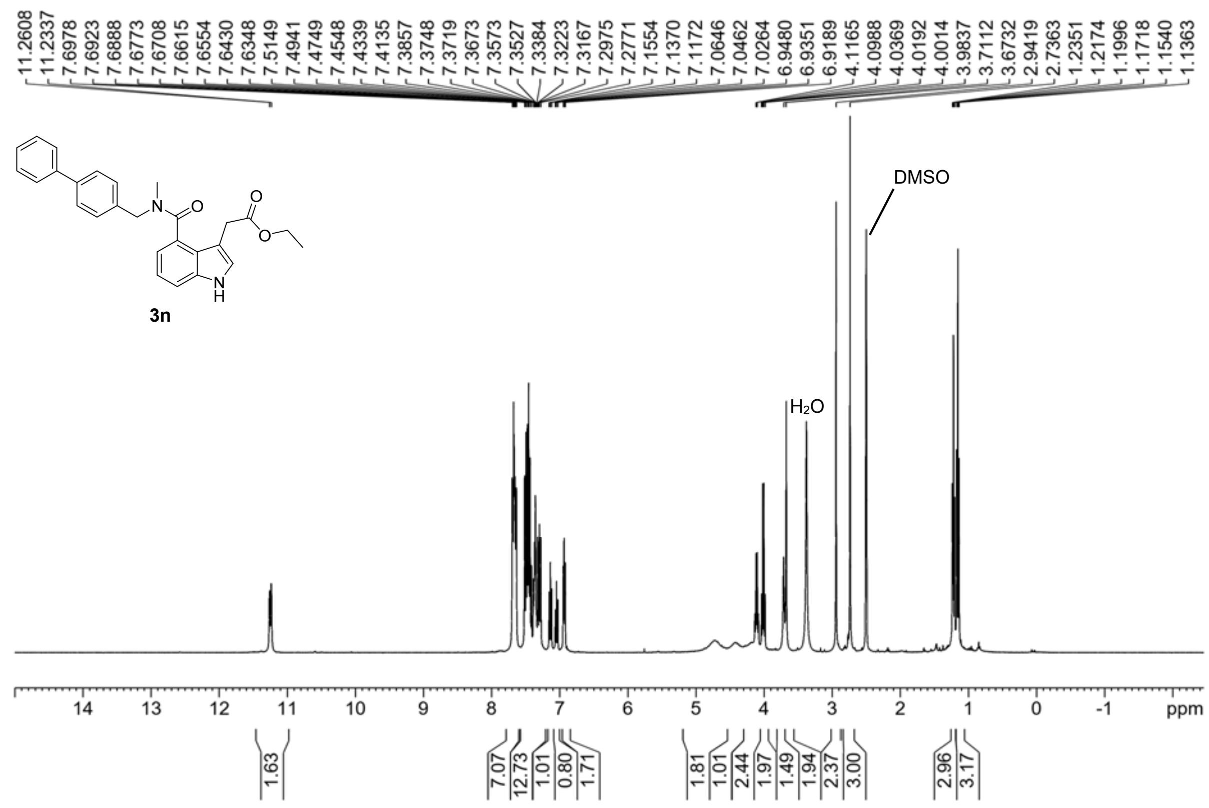
11.2608
11.2337
7.6978
7.6923
7.6888
7.6773
7.6708
7.6615
7.6554
7.6430
7.6348
7.5149
7.4941
7.4749
7.4548
7.4339
7.4135
7.3857
7.3748
7.3719
7.3673
7.3573
7.3527
7.3384
7.3223
7.3167
7.2975
7.2771
7.1554
7.1370
7.1172
7.0646
7.0462
7.0264
6.9480
6.9351
6.9189
4.1165
4.0988
4.0369
4.0192
4.0014
3.9837
3.7112
3.6732
2.9419
2.7363
1.2351
1.2174
1.1996
1.1718
1.1540
1.1363
O
N
O
O
NH
3n
DMSO
H_2O
14 13 12 11 10 9 8 7 6 5 4 3 2 1 0 -1 ppm
1.63
7.07
12.73
1.01
0.80
1.71
1.81
1.01
2.44
1.97
1.49
1.94
2.37
3.00
2.96
3.17

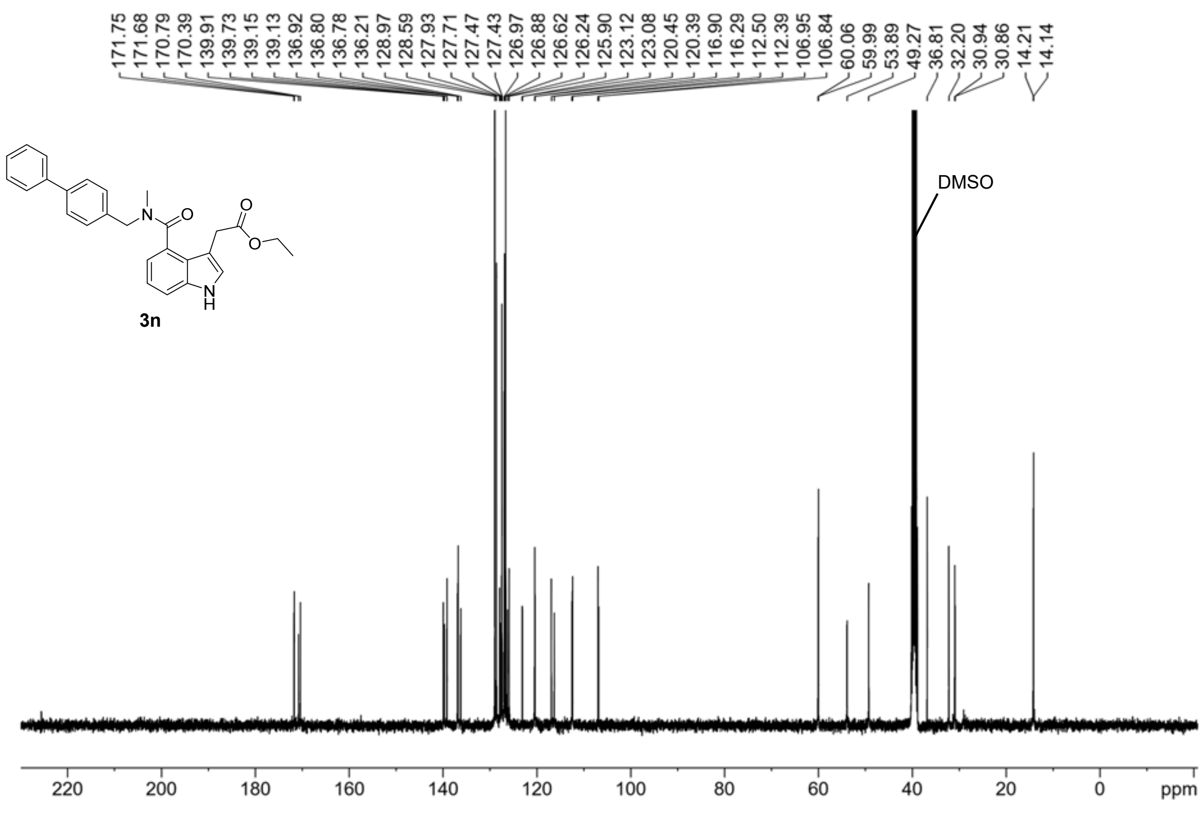
171.75
171.68
170.79
170.39
139.91
139.73
139.15
139.13
136.92
136.80
136.78
136.21
128.97
128.59
127.93
127.71
127.47
127.43
126.97
126.88
126.62
126.24
125.90
123.12
123.08
120.45
120.39
116.90
116.29
112.50
112.39
106.95
106.84
60.06
59.99
53.89
49.27
36.81
32.20
30.94
30.86
14.21
14.14
O
N
O
O
NH
3n
DMSO
220 200 180 160 140 120 100 80 60 40 20 0 ppm

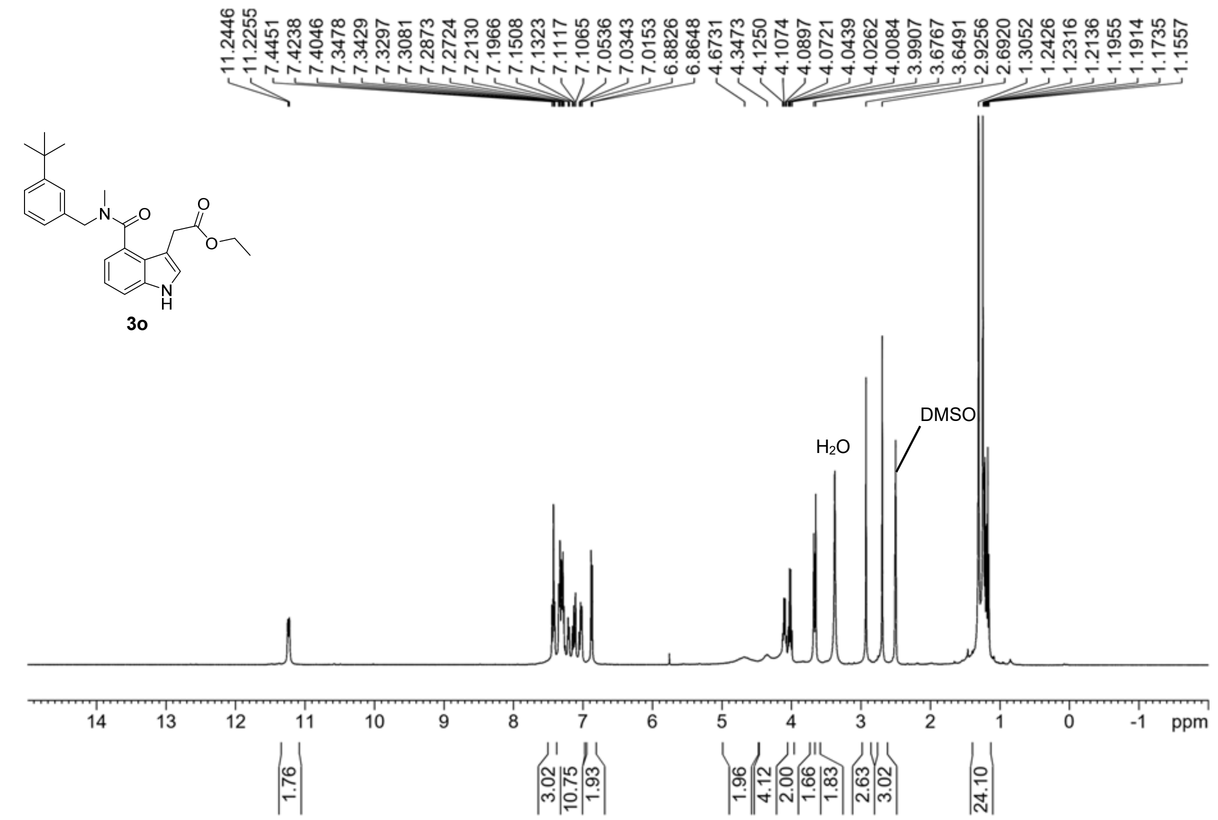
11.2446
11.2255
7.4451
7.4238
7.4046
7.3478
7.3429
7.3297
7.3081
7.2873
7.2724
7.2130
7.1966
7.1508
7.1323
7.1117
7.1065
7.0536
7.0343
7.0153
6.8826
6.8648
4.6731
4.3473
4.1250
4.1074
4.0897
4.0721
4.0439
4.0262
4.0084
3.9907
3.6767
3.6491
2.9256
2.6920
1.3052
1.2426
1.2316
1.2136
1.1955
1.1914
1.1735
1.1557
O
N
O
O
N
H
3o
DMSO
H_2O
14
13
12
11
10
9
8
7
6
5
4
3
2
1
0
-1
ppm
1.76
3.02
10.75
1.93
1.96
4.12
2.00
1.66
1.83
2.63
3.02
24.10

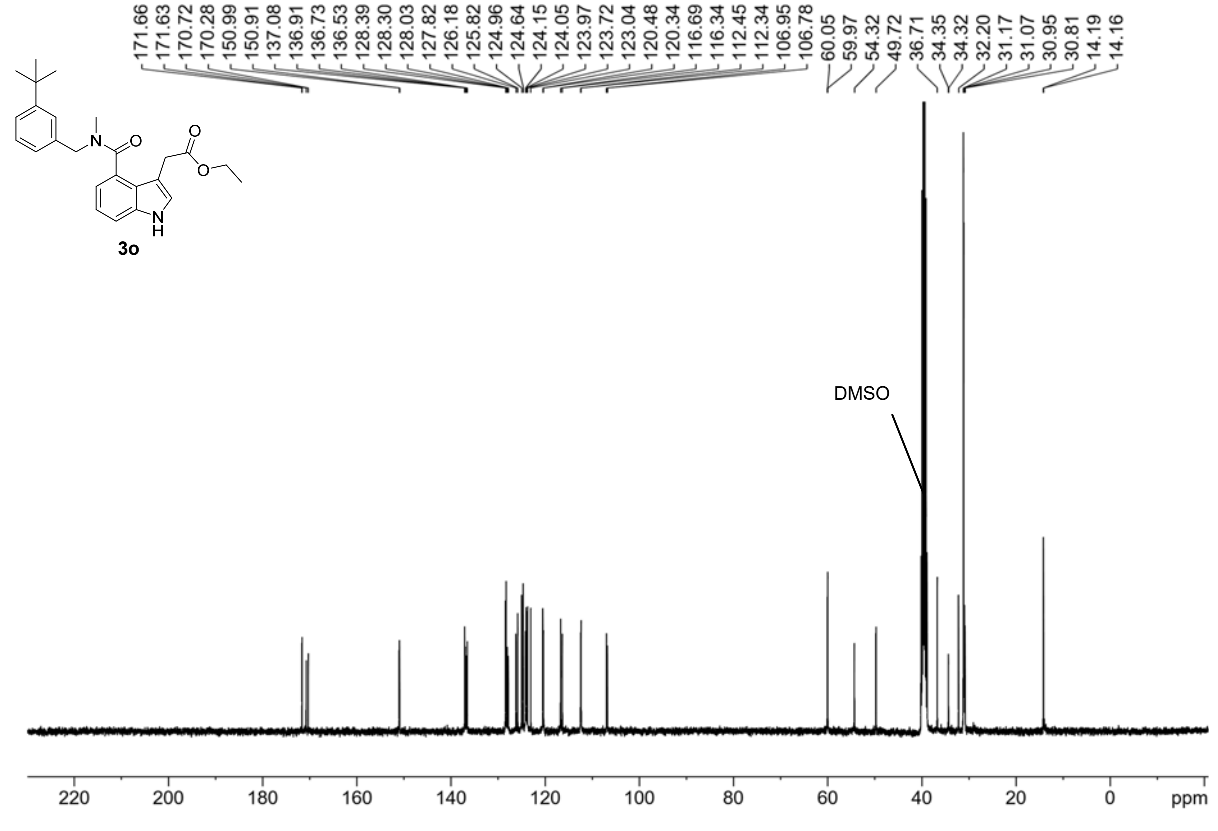
171.66
171.63
170.72
170.28
150.99
150.91
137.08
136.91
136.73
136.53
128.39
128.30
128.03
127.82
126.18
125.82
124.96
124.64
124.15
124.05
123.97
123.72
123.04
120.48
120.34
116.69
116.34
112.45
112.34
106.95
106.78
60.05
59.97
54.32
49.72
36.71
34.35
34.32
32.20
31.17
31.07
30.95
30.81
14.19
14.16
O
N
O
O
N
H
3o
DMSO
220
200
180
160
140
120
100
80
60
40
20
0
ppm

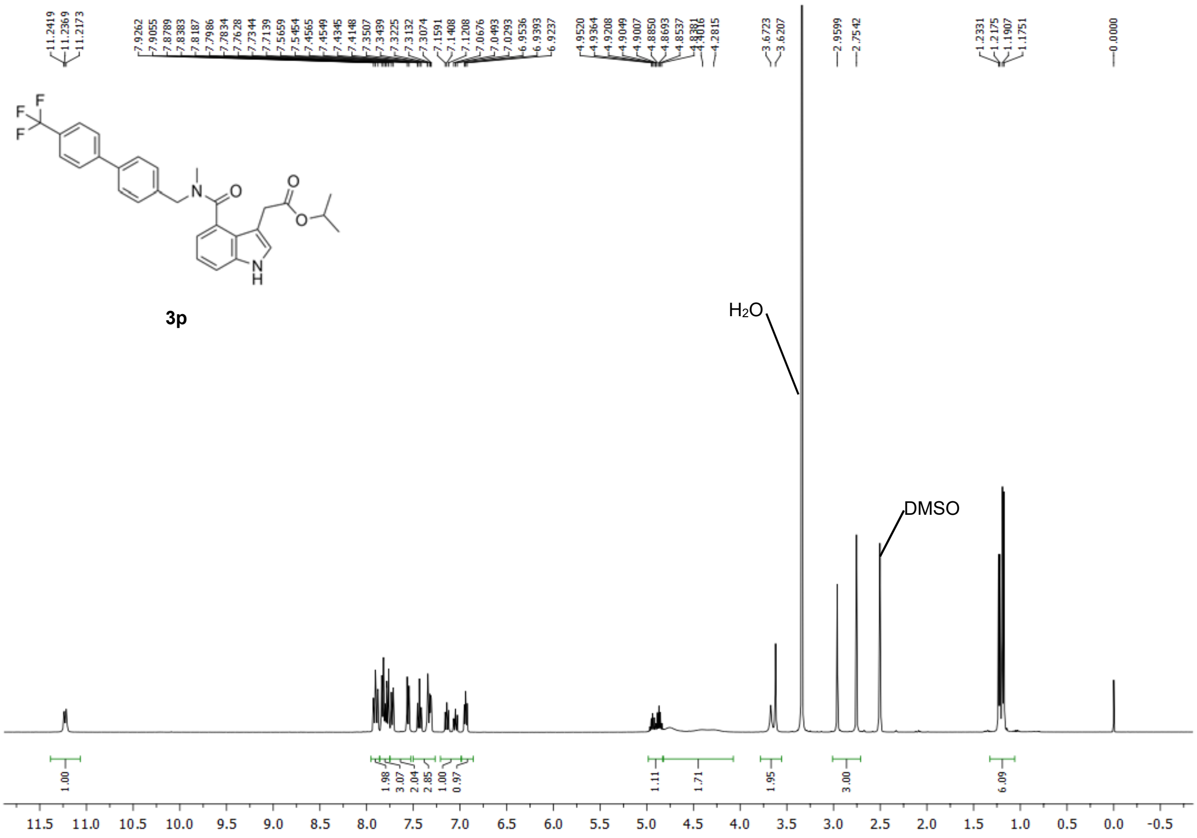
3p
H2O
DMSO

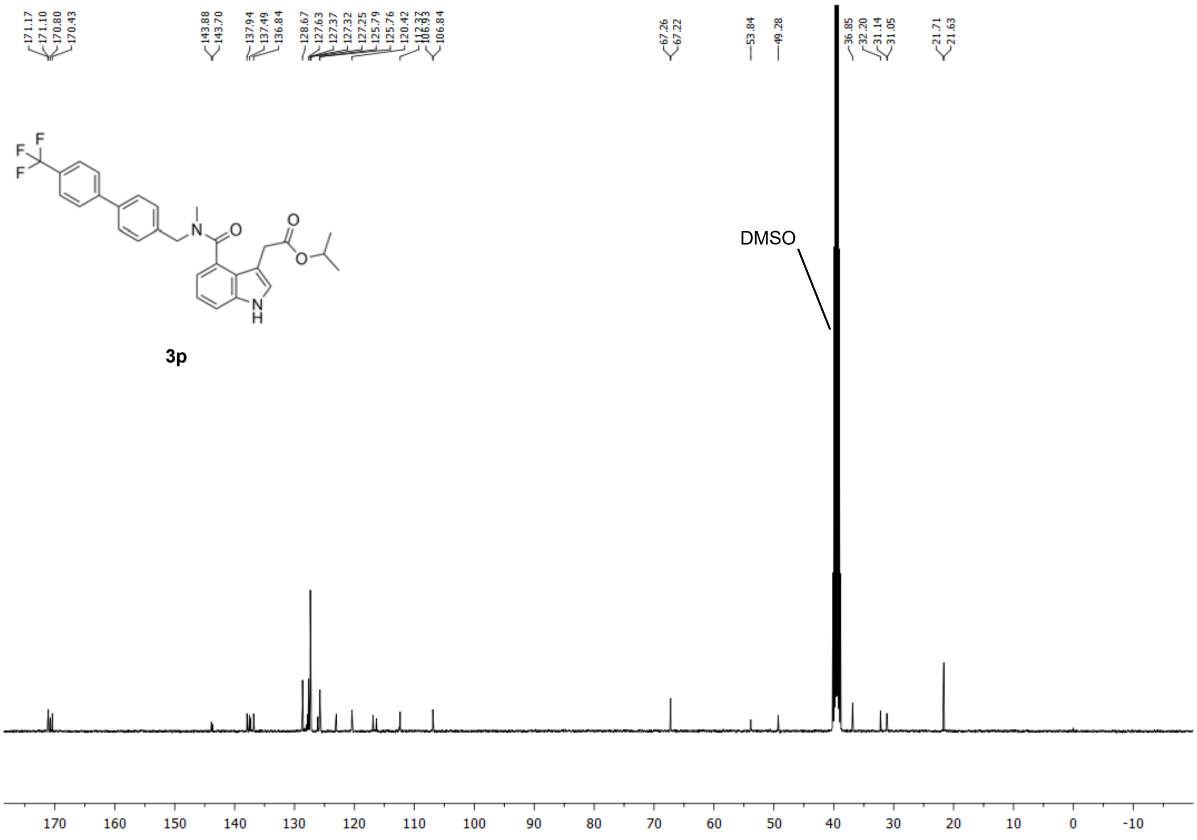
3p
DMSO

HPLC chromatograms of 2p,r and 3a-p

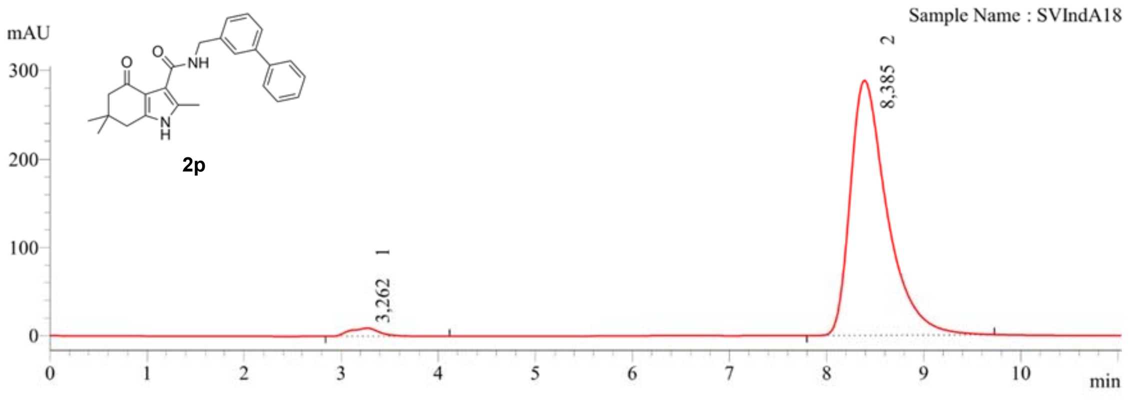

Peak Table

PDA Ch1 254nm

Peak#	Ret. Time	Area	Area%	Height
1	3,262	195545	2,513	9180
2	8,385	7585356	97,487	288044
Total		7780901	100,000	297223

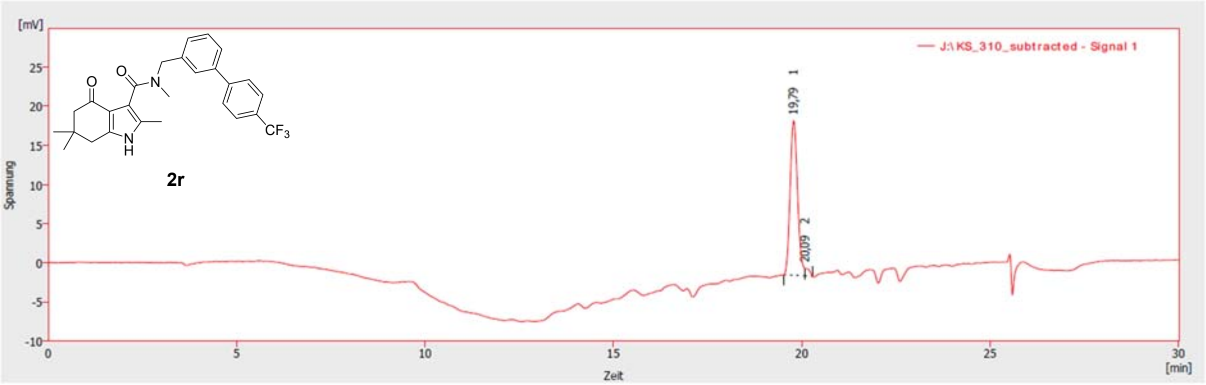

Ergebnistabelle (Nicht kal. - J:|KS_310_subtracted - Signal 1)

	Retentionsz. [min]	Fläche [mV.sek]	Höhe [mV]	Fläche [%]	Höhe [%]	W05 [min]	Substanzname
1	19,785	270,588	19,772	97,2	95,5	0,21	
2	20,088	7,802	0,924	2,8	4,5	0,14	
	Gesamt	278,390	20,695	100,0	100,0		

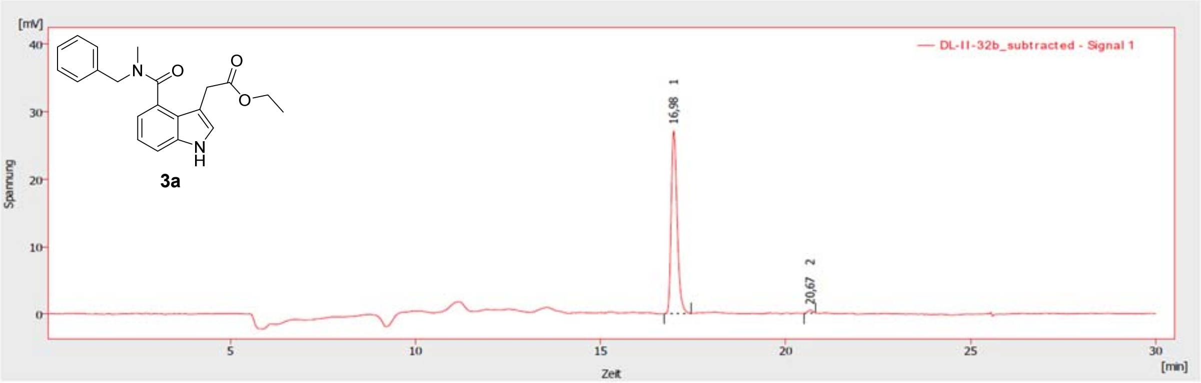

Ergebnistabelle (Nicht kal. - DL-II-32b_subtracted - Signal 1)

	Retentionsz. [min]	Fläche [mV.sek]	Höhe [mV]	Fläche [%]	Höhe [%]	W05 [min]	Substanzname
1	16,980	304,570	27,245	98,6	98,3	0,17	
2	20,675	4,225	0,474	1,4	1,7	0,14	
	Gesamt	308,794	27,719	100,0	100,0		

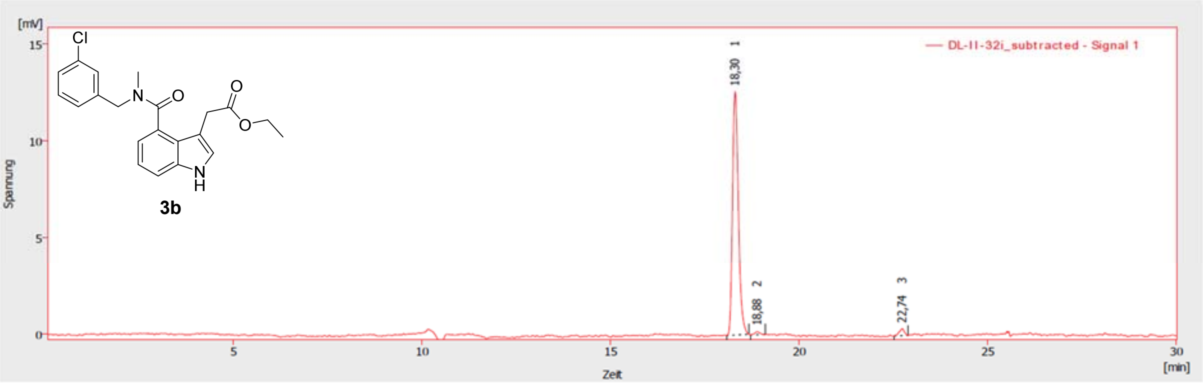

Ergebnistabelle (Nicht kal. - DL-II-32i_subtracted - Signal 1)

	Retentionsz. [min]	Fläche [mV.sek]	Höhe [mV]	Fläche [%]	Höhe [%]	W05 [min]	Substanzname
1	18,300	135,922	12,563	96,5	96,0	0,17	
2	18,884	1,556	0,161	1,1	1,2	0,14	
3	22,736	3,412	0,363	2,4	2,8	0,15	
	Gesamt	140,890	13,088	100,0	100,0		

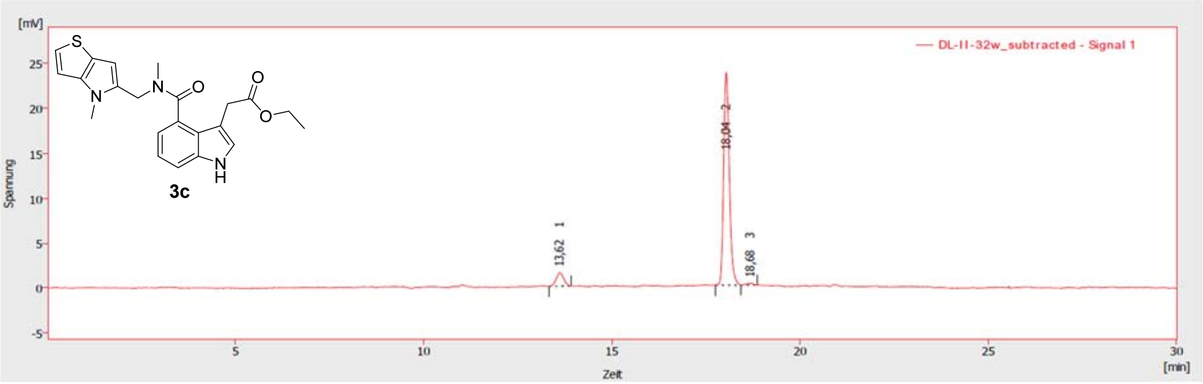

Ergebnistabelle (Nicht kal. - DL-II-32w_subtracted - Signal 1)

	Retentionsz. [min]	Fläche [mV.sek]	Höhe [mV]	Fläche [%]	Höhe [%]	W05 [min]	Substanzname
1	13,616	20,665	1,487	7,5	5,9	0,22	
2	18,039	250,637	23,666	91,4	93,3	0,16	
3	18,676	2,775	0,222	1,0	0,9	0,19	
	Gesamt	274,077	25,375	100,0	100,0		

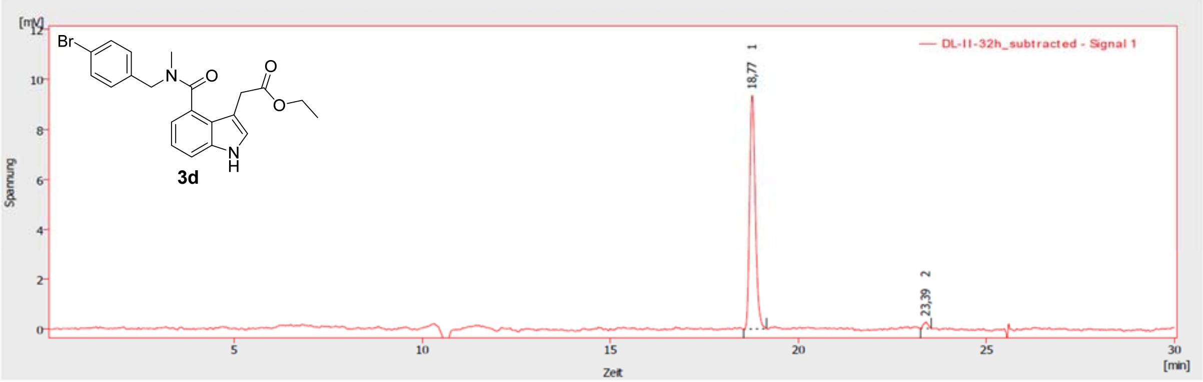

Ergebnistabelle (Nicht kal. - DL-II-32h_subtracted - Signal 1)

	Retentionsz. [min]	Fläche [mV.sek]	Höhe [mV]	Fläche [%]	Höhe [%]	W05 [min]	Substanzname
1	18,775	99,414	9,351	97,8	97,3	0,17	
2	23,393	2,261	0,261	2,2	2,7	0,15	
	Gesamt	101,674	9,613	100,0	100,0		

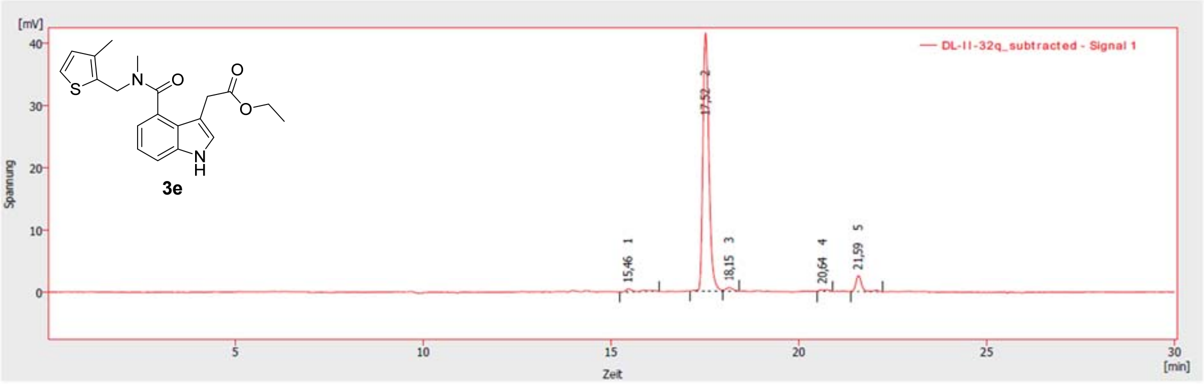

3e

Ergebnistabelle (Nicht kal. - DL-II-32q_subtracted - Signal 1)

	Retentionsz. [min]	Fläche [mV.sek]	Höhe [mV]	Fläche [%]	Höhe [%]	W05 [min]	Substanzname
1	15,457	8,871	0,503	1,7	1,1	0,15	
2	17,519	461,927	41,464	90,5	91,3	0,17	
3	18,151	6,742	0,542	1,3	1,2	0,19	
4	20,640	4,037	0,258	0,8	0,6	0,29	
5	21,588	28,954	2,632	5,7	5,8	0,15	
	Gesamt	510,531	45,399	100,0	100,0		

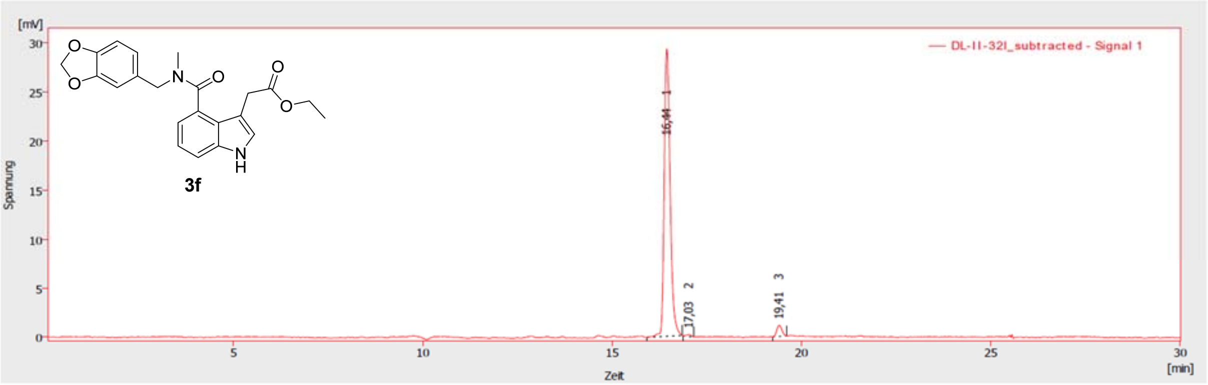

3f

Ergebnistabelle (Nicht kal. - DL-II-32l_subtracted - Signal 1)

	Retentionsz. [min]	Fläche [mV.sek]	Höhe [mV]	Fläche [%]	Höhe [%]	W05 [min]	Substanzname
1	16,444	317,933	29,276	96,3	95,7	0,17	
2	17,025	1,725	0,198	0,5	0,6	0,18	
3	19,411	10,630	1,123	3,2	3,7	0,15	
	Gesamt	330,287	30,597	100,0	100,0		

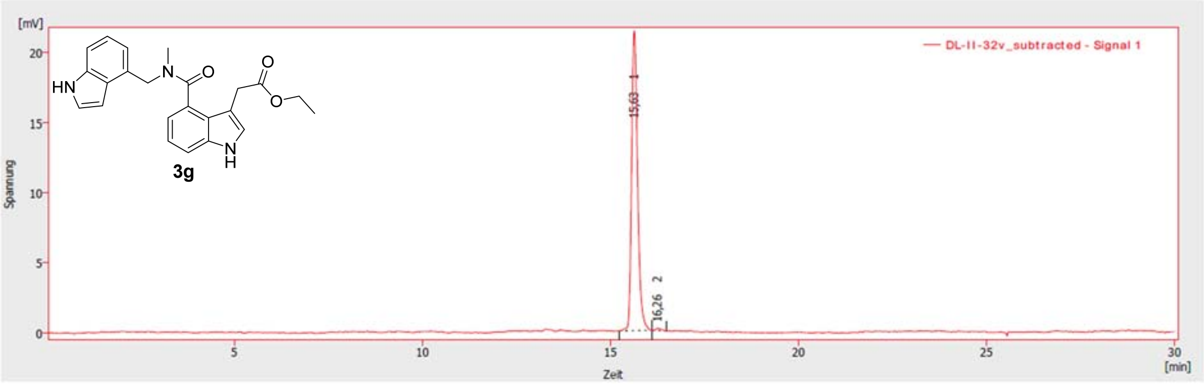

3g

Ergebnistabelle (Nicht kal. - DL-II-32v_subtracted - Signal 1)

	Retentionsz. [min]	Fläche [mV.sek]	Höhe [mV]	Fläche [%]	Höhe [%]	W05 [min]	Substanzname
1	15,635	236,112	21,357	99,3	99,2	0,17	
2	16,256	1,715	0,175	0,7	0,8	0,14	
	Gesamt	237,827	21,532	100,0	100,0		

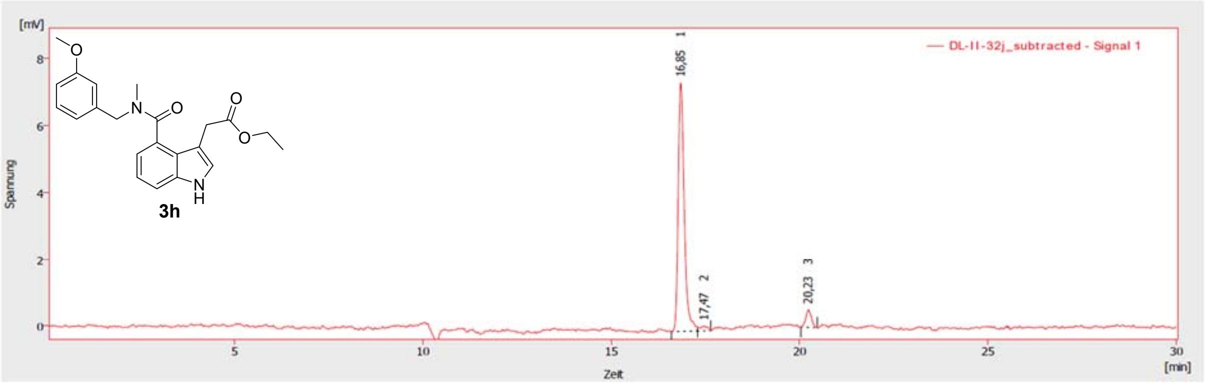

Ergebnistabelle (Nicht kal. - DL-II-32j_subtracted - Signal 1)

	Retentionsz. [min]	Fläche [mV.sek]	Höhe [mV]	Fläche [%]	Höhe [%]	W05 [min]	Substanzname
1	16,845	83,305	7,432	91,6	91,7	0,17	
2	17,471	2,132	0,142	2,3	1,8	0,31	
3	20,231	5,464	0,529	6,0	6,5	0,16	
	Gesamt	90,901	8,103	100,0	100,0		

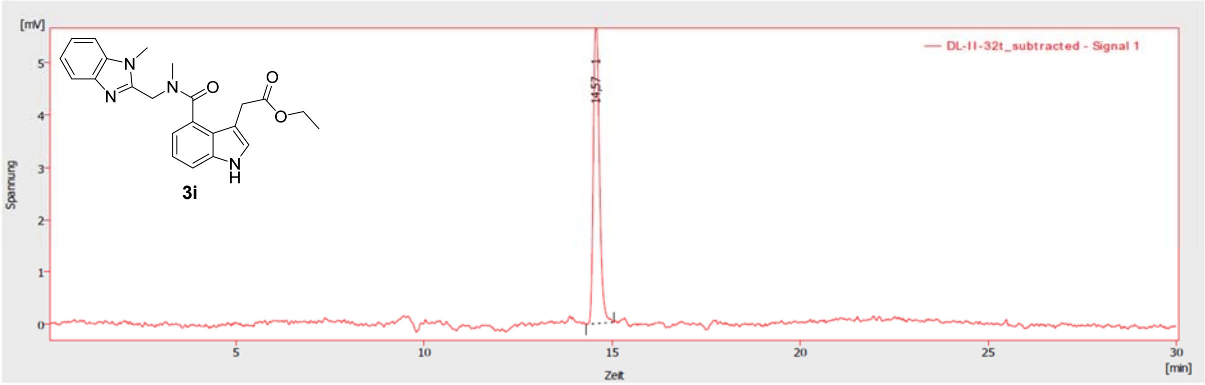

Ergebnistabelle (Nicht kal. - DL-II-32t_subtracted - Signal 1)

	Retentionsz. [min]	Fläche [mV.sek]	Höhe [mV]	Fläche [%]	Höhe [%]	W05 [min]	Substanzname
1	14,571	65,302	5,904	100,0	100,0	0,17	
	Gesamt	65,302	5,904	100,0	100,0		

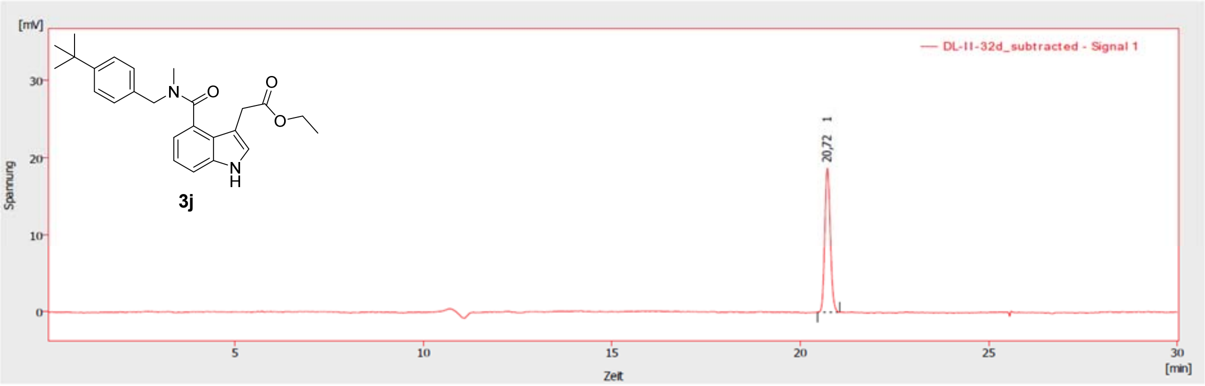

Ergebnistabelle (Nicht kal. - DL-II-32d_subtracted - Signal 1)

	Retentionsz. [min]	Fläche [mV.sek]	Höhe [mV]	Fläche [%]	Höhe [%]	W05 [min]	Substanzname
1	20,719	192,428	18,708	100,0	100,0	0,16	
	Gesamt	192,428	18,708	100,0	100,0		

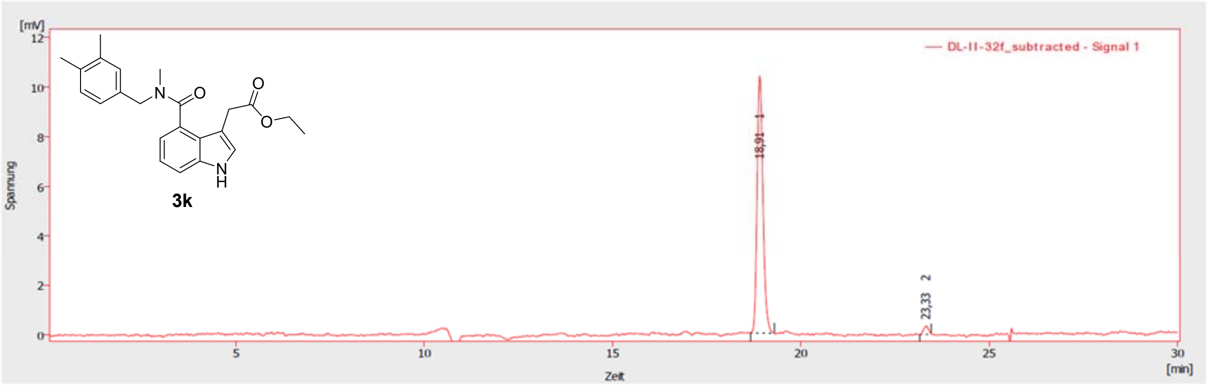

Ergebnistabelle (Nicht kal. - DL-II-32f_subtracted - Signal 1)

	Retentionsz. [min]	Fläche [mV.sek]	Höhe [mV]	Fläche [%]	Höhe [%]	W05 [min]	Substanzname
1	18,908	112,614	10,369	97,3	96,8	0,17	
2	23,331	3,081	0,340	2,7	3,2	0,15	
	Gesamt	115,695	10,708	100,0	100,0		

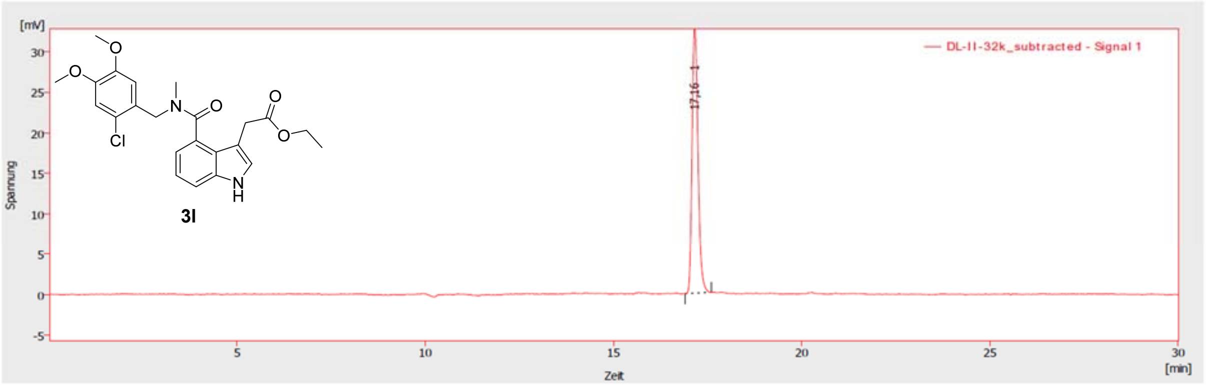

Ergebnistabelle (Nicht kal. - DL-II-32k_subtracted - Signal 1)

	Retentionsz. [min]	Fläche [mV.sek]	Höhe [mV]	Fläche [%]	Höhe [%]	W05 [min]	Substanzname
1	17,160	359,559	32,939	100,0	100,0	0,17	
	Gesamt	359,559	32,939	100,0	100,0		

Sample Name : CG130.1

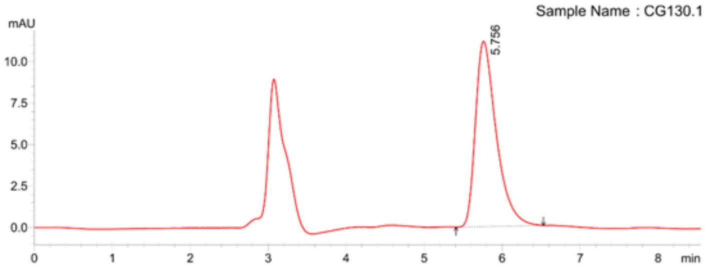

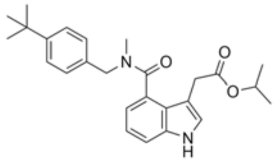

3m

PeakTable

PDA Ch1 254nm 4nm

Peak#	Ret. Time	Area	Height	Area %	Height %
1	5.756	207960	11185	100.000	100.000
Total		207960	11185	100.000	100.000

MS Chromatogram

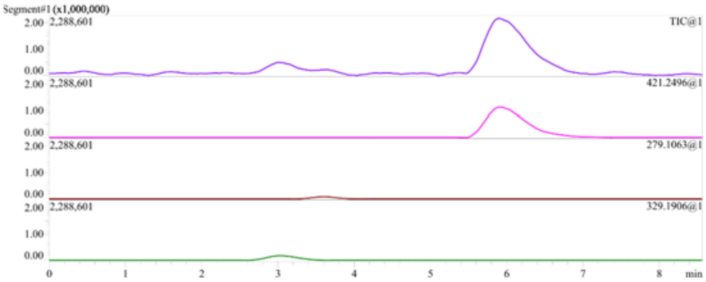

Peak 1 refers to plasticizers dibutylphthalate (DBP, 279 m/z) and di-*n*-pentylphthalate (DNPP, 329 m/z) as minor contaminants in these analytical runs.

3m purity is >95% (^{1}H NMR)

DBP $[M+H]^+$

DNPP $[M+Na]^+$

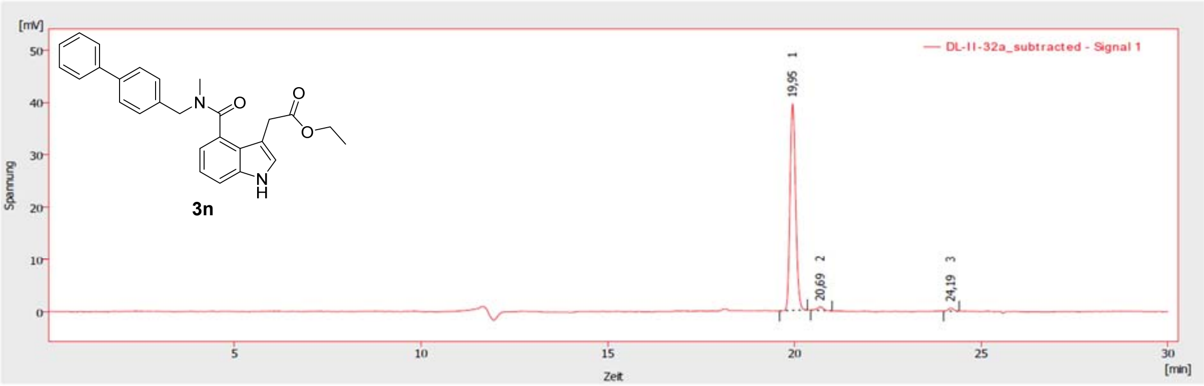

Ergebnistabelle (Nicht kal. - DL-II-32a_subtracted - Signal 1)

	Retentionsz. [min]	Fläche [mV.sek]	Höhe [mV]	Fläche [%]	Höhe [%]	W05 [min]	Substanzname
1	19,951	414,331	39,506	96,7	96,8	0,16	
2	20,695	8,088	0,699	1,9	1,7	0,16	
3	24,187	5,982	0,618	1,4	1,5	0,15	
	Gesamt	428,401	40,823	100,0	100,0		

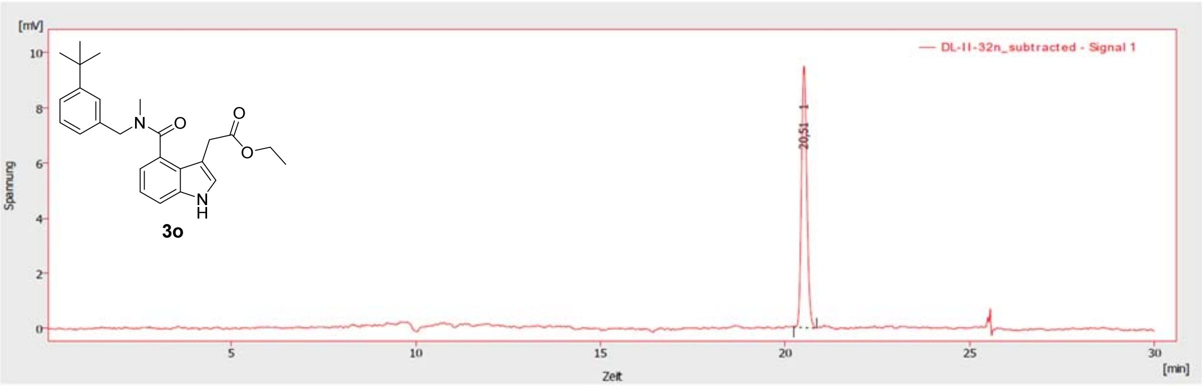

Ergebnistabelle (Nicht kal. - DL-II-32n_subtracted - Signal 1)

	Retentionsz. [min]	Fläche [mV.sek]	Höhe [mV]	Fläche [%]	Höhe [%]	W05 [min]	Substanzname
1	20,512	96,980	9,484	100,0	100,0	0,16	
	Gesamt	96,980	9,484	100,0	100,0		

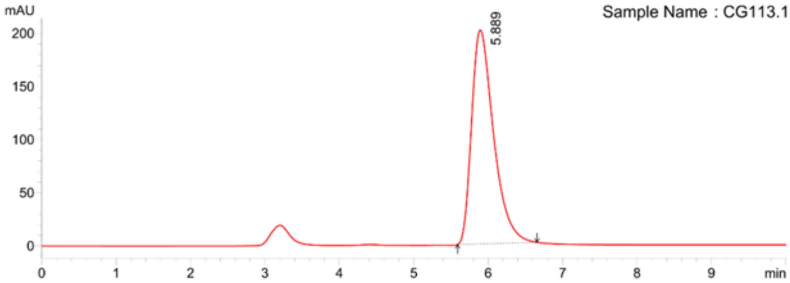

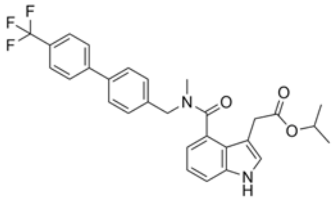

3p

PeakTable

PDA Ch1 254nm 4nm

Peak#	Ret. Time	Area	Height	Area %	Height %
1	5.889	4135448	201262	100.000	100.000
Total		4135448	201262	100.000	100.000

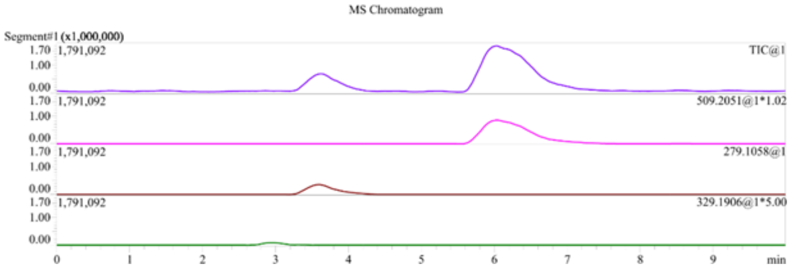

Peak 1 refers to plasticizers dibutylphthalate (DBP, 279 m/z) and di-*n*-pentylphthalate (DNPP, 329 m/z) as minor contaminants in these analytical runs.

3p purity is >95% (^{1}H NMR)

DBP $[M+H]^+$

DNPP $[M+Na]^+$

Single crystal structure analysis of (−)-1b

Data collection was performed with an XtaLAB Synergy, Dualflex, HyPix diffractometer using CuKα-radiation. The structure was solved with SHELXT[1] and refined with SHELXL[2] using Least Squares minimisation. All non-hydrogen atoms were refined anisotropic. The C-H H-atoms were located in difference map but were positioned with idealized geometry (methyl H-atoms allowed to rotate but not to tip) and were refined isotropic with $U_{iso}(H) = 1.2\ U_{eq}(C)$ (1.5 for methyl H-atoms) using a riding model. The N-H H-atoms were also located in difference map and refined isotropic with restraints (DFIX). Two methyl groups are disordered and were refined using a split model with restraints (SADI). The crystal is pseudo-merohedrally twinned with lattice parameters close to that expected for an orthorhombic crystal system, but the structure analysis clearly shows, that the compound crystallizes monoclinic. Therefore, a twin refinement was performed, leading to a BASF parameter of 0.629 (1).

The absolute configuration was determined and is in agreement with the selected setting (Flack X parameter 0.000(5) by classical fit to all intensities and -0.008(2) from 13077 selected quotients (Parsons' method)). The asymmetric unit consists of six crystallographically independent molecules that differ in their conformation. CCDC-2299119 contain the supplementary crystallographic data for this paper.
These data can be obtained free charge from the Cambridge Crystallographic Data Centre via http://www.ccdc.cam.ac.uk/data_request/cif.

Supplemental References

[1] SHELXT Sheldrick, G.M. (2015). Acta Cryst. A71, 3-8.

[2] SHELXL Sheldrick, G.M. (2015). Acta Cryst. C71, 3-8.
